# In-vitro model for bacterial growth inhibition of compartmentalized infection treated by an ultra-high concentration of antibiotics

**DOI:** 10.1371/journal.pone.0252724

**Published:** 2021-06-08

**Authors:** Moris Topaz, Abed Athamna, Itamar Ashkenazi, Baruch Shpitz, Sarit Freimann

**Affiliations:** 1 Clinical Microbiology Laboratory, Hillel Yaffe Medical Center, Hadera, Israel; 2 Bruce Rappaport School of Medicine, Technion, Haifa, Israel; 3 Sackler School of Medicine, Tel-Aviv University, Tel Aviv, Israel; University of Hong Kong, HONG KONG

## Abstract

**Background:**

*Pseudomonas aeruginosa (P*. *aeruginosa)*, *Escherichia coli (E*. *coli)*, and *Staphylococcus aureus (S*. *aureus)* are common pathogens encountered in infected cardiovascular-implantable electronic device (CIED). Continuous, in-situ targeted, ultra-high concentration antibiotic (CITA) treatment is a novel antibiotic treatment approach for localized infections. CITA provides sufficient local antibiotic concentrations to heavily infected cavities while avoiding systemic toxicity.

**Aim:**

*In-vitro* confirmation of the efficacy of the CITA treatment approach in simulated compartmentalized infections.

**Materials and methods:**

A rapid automated bacterial culture analyzing system) Uro4 HB&L^™^ (was applied to compare the efficacy of selected antibiotics at a standard minimal inhibitory concentration (1MIC), 4MIC, and CITA at 10^3^MIC, for growth inhibition of high bacterial loads (10^6^ colony-forming-units/ml) of ATCC strains of *P*. *aeruginosa*, *E*. *coli*, and *S*. *aureus*.

**Results:**

The addition of gentamicin and amikacin at 1MIC concentrations only temporarily inhibited the exponential growth of *E*. *coli* and *P*. *aeruginosa*. 4MIC level extended the delay of exponential bacterial growth. Increasing concentrations of vancomycin similarly temporarily delayed *S*. *aureus* growth. All tested antibiotics at CITA of 10^3^MIC totally inhibited the exponential growth of the tested bacteria through 72 hours of exposure. (P<0.001).

**Conclusion:**

In this in-vitro model, CITA at 10^3^MIC effectively inhibited exponential bacterial growth of high loads of *P*. *aeruginosa*, *E*. *coli*, and *S*. *aureus*. This model offers preliminary laboratory support for the benefit of the in-situ antibiotic treatment, providing ultra-high concentrations directly at the compartmentalized infection site, not achievable by the conventional intravenous and oral routes.

## Introduction

The conventional intravenous and oral routes of antibiotic delivery are limited as a single treatment modality of infected cardiovascular implantable electronic devices (CIEDs) and other prosthetic biofilm-related infections such as prosthetic joint infection [1, 2]. This limitation is mainly due to the inadequate effect of minimal inhibitory concentration (MIC) of antibiotic levels to eradicate high concentrations of bacteria and biofilm growth at the infection sites, establishing the wide acceptance of the need for complete removal of the infected CIEDs [3, 4]. The need for complete extraction of CIEDs is associated with significant morbidity and potentially life-threatening complications [5, 6].

The systemic toxicity of the various antibiotic agents limit local levels at the infection site to below those effective in treating heavily infected cavities and the minimum biofilm eradication concentration (MBEC) for local biofilm growth [7]. Ultra-high levels in the range of 10^2^–10^3^ are required to eradicate high bacterial loads and biofilm [8–10]. It is accepted that exposure to sub-inhibitory levels of one antibiotic can select for high-level tolerance to multiple and dissimilar antibiotics [11].

The continuous, in-situ targeted, ultra-high concentration antibiotic (CITA) treatment method delivers antibiotics directly to the infection site at concentrations in the range of 10^2^–10^3^-fold higher than the respected standard serum MIC (Clinical and Laboratory Standards Institute) [12]. The CITA treatment is not dependent on tissue vascularization and achieves concentrations that are orders of magnitude higher than systemic administration would be able to deliver safely.

The rationale of local antibiotic delivery is to enhance local antibiotic soft tissue concentration and eradicate the remaining biofilm and prevent recolonization. Staphylococcus aureus (*S*. *aureus*), Pseudomonas aeruginosa (*P*. *aeruginosa*) and Escherichia coli (*E*. *coli*) are the most prevalent soft tissue pathogens with the first two are known to form biofilms in soft tissues infection associated with the presence of foreign bodies [2, 3, 13].

We have been using the CITA method for more than ten years to treat infected CIEDs with relatively high salvage rate [14, 15] and infected, complex wounds, including early and immediate closure of combat injuries [16]. The recommended daily dose is administered over 24 hours by continuous drip, directly into the compartmentalized infection site, instead of intravenously, generating ultra-high local antibiotic concentrations at the infection site. CITA at 10^2^-10^3^MIC of gentamicin and vancomycin were proven safe, with stable desired serum levels and a high salvage rate of CIEDs [15].

In this study, we evaluated the efficacy of the 10^3^MIC CITA method in growth inhibition of high bacterial loads (10^6^ CFU/ml) in an in-vitro conceptualized model. The conventional antibiotic susceptibility testing (AST) assays (e.g. The disk defusion test) are designed for the determination of the susceptibility of bacteria to varying antibiotic concentrations [17]. Most current AST assays correspond to bacterial sensitivities at the range of 1MIC antibiotics, yet not applicable for antibiotics at the range of 10^2^–10^3^ MIC. Alternative laboratory tests and tooling are required to evaluate the efficacy of the CITA treatment method. A fast, automated bacterial culture Uro4 HB&L^™^ analyzing system (HB&L) was introduced by Alifax (Padova, Italy) for bacteriuria, bacteremia, and biological fluid screening [18]. The device scans the samples and translates light scattering signals into bacterial growth curves within a short time frame (a few hours to 24 hours).

The main objective of this study was to determine in-vitro the efficacy of selected antibiotics, at concentrations of 10^3^MIC, in suppressing bacterial growth of pathogens commonly encountered in localized infected wounds at high bacterial loads, 10^6^ colony-forming units (CFU/ml). Bacterial growth rates without antibiotics (0MIC), bacterial growth rates at antibiotic concentrations representing the standard dosage for intravenous or oral routes (1MIC), and bacterial growth rates at 4MIC served as controls.

## Materials and methods

This was an in-vitro model using standard HB&L vials with growth medium at 37°C that were inoculated with high concentrations (10^6^ CFU/ml) of *P*. *aeruginosa*, *E*. *coli*, and *S*. *aureus* in order to simulate localized infected wounds. The inocula were then exposed to various concentrations of appropriate antibiotics in order to determine the effect of concentration on the rate and time to exponential bacterial growth (EBG).

### Microorganism concentration analysis by the HB&L system

Bacterial growth inhibition in the presence of antibiotics was determined using the HB&L system. The HB&L system registers deviation of light passing through a microorganism sample growing in a light-transparent liquid. The technology utilizes a repeated polarized, collimated, focused light beam oriented through the sample in 5-min intervals. The number, mass, shape, and dimensions of microorganisms impact the refractive index of the sample. The signal is then processed by detectors placed at two different angles (30° and 90°). The light scattering technology detects the change in bacteria concentration within a few hours of initial bacterial growth. The signals are processed by the software, which monitors the turbidity of the solution generated by the growth of bacteria during the time frame and converts the light signals to bacterial CFU/ml. A magnetic stirrer bar homogenizes the vial content before each reading, and the growth of microorganisms is monitored in real-time by displaying growth curves on the computer screen. The time of the EBG is derived from the growth curve, or the list of optical density data reads.

### Antibiotics and preparation of microorganism

Antibiotic solutions of gentamicin (VETPROM AD, Bulgaria), amikacin (Anfarm Hellas S.A., Attika, Greece), and vancomycin (Laboratorio Ramon Sala. S.L. Barcelona, Spain) were diluted in 0.9% w/v sodium chloride solution (normal saline; B.Braun, Germany) to the desired 1MIC, 4MIC and ultra-high concentrations (10^3^MIC) for each antibiotic at the respected standard MIC concentrations for each bacterial strain [12].

### Experimental details

**Fig 1:**
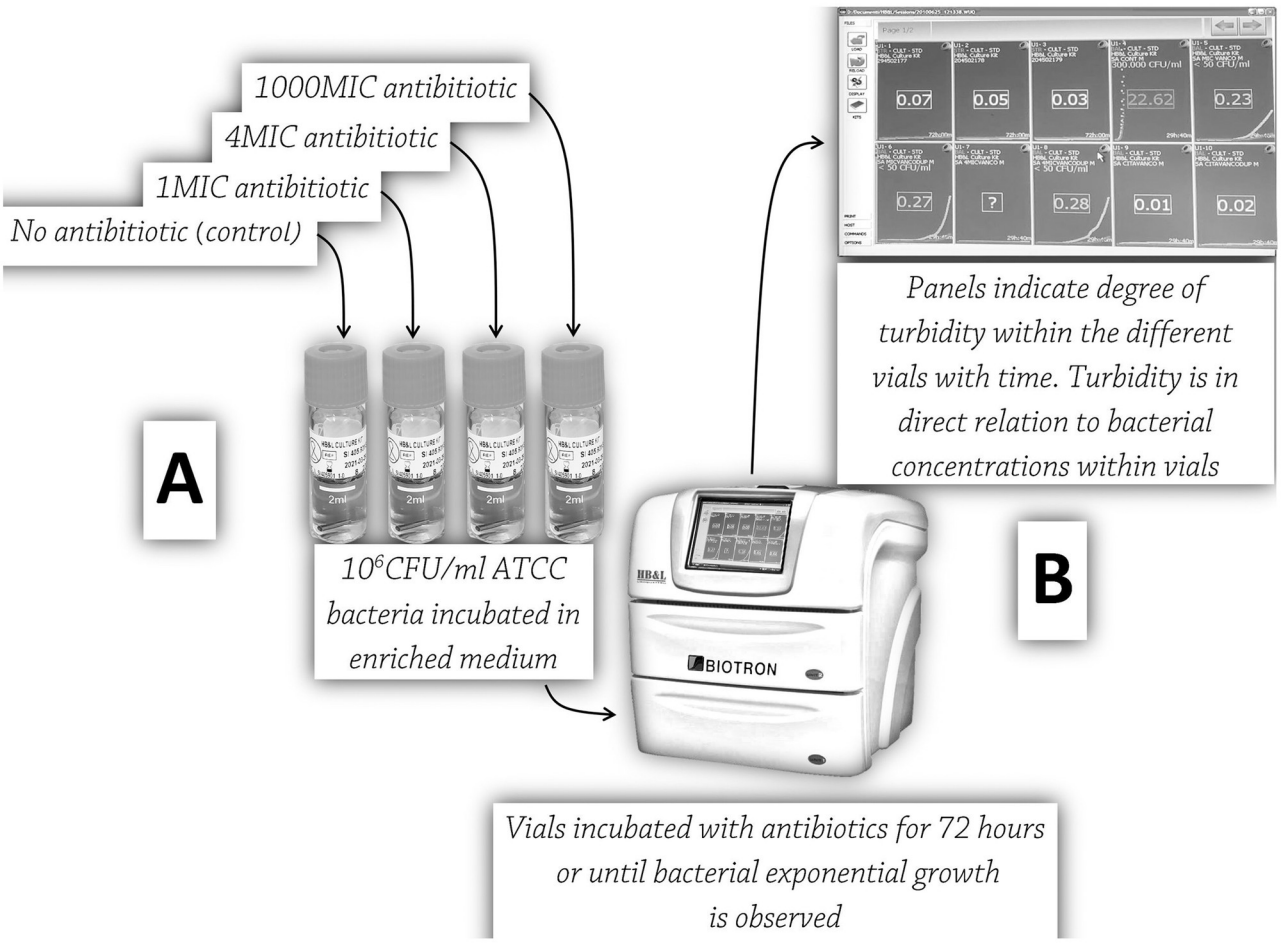
Study design scheme: (A). Vials containing ATCC bacteria at a concentration of 10^6^ colony forming units (CFU/ml) were incubated in enriched medium and different antibiotic concentrations; (B). Turbidity measured by the HB&L system within the vial indicating the time of bacterial exponential growth.

The study design is schematically presented in Fig 1. In an experimental set, 10^6^ CFU/ml of bacteria were incubated with 0MIC, 1MIC, 4MIC, and 10^3^MIC antibiotic solution. The time of EBG was recorded by the HB&L system. The experiments were done in duplicates except for 0MIC. Three representative bacterial strains; *P*. *aeruginosa* (ATCC^®^ 27853^™^), *E*. *coli* (ATCC^®^ 25922^™^ and 35218^™^) and *S*. *aureus* (ATCC^®^ 25913^™^ and 25923^™^) were investigated. Three to five colonies of overnight grown cultures on blood agar at 35°C were suspended in sterile normal saline. Standardized organism suspensions were adjusted to 0.5 McFarland (~10^8^CFU/ml) and diluted (1:10) in sterile normal saline solution to obtain an inoculum of bacterial concentration of 10^7^ CFU/ml. A 0.1ml suspension of the inoculum was then added to 2 ml standard HB&L-enriched medium vials (final inoculum, 0.5 10^6^ CFU/ml). A 0.6ml solution of each antibiotic, at final concentrations of 1MIC gentamicin at (4mg/L) for *P*. *aeruginosa* and *E*. *coli*, amikacin at (16mg/L) for *P*. *aeruginosa* and *E*. *coli* and vancomycin at MIC (2mg/L) for *S*. *aureus*, was added to the experimental vial. 4MIC and 10^3^MIC were prepared, respectively. A 0.6ml of normal saline was added to the 0MIC controls. The vials were then placed in the incubation chamber of the HB&L device, at 37°C, with stirring. Samples were continuously monitored by the HB&L for 72 hours to determine EBG. All experimental test samples, together with controls, were prepared and monitored in parallel by the HB&L.

### Data analysis

EBG of ATCC strains of *E*. *coli* and *P*. *aeruginosa* at a concentration of 10^6^ CFU/ml were evaluated in standard vials containing 0, 1, 4, and 10^3^MIC of either gentamicin or amikacin, respectively. EBG of ATCC strains of *S*. *aureus* at 10^6^ CFU/ml was evaluated in standard vials containing 0, 1, 4, and 10^3^MIC of vancomycin. The timing of EBG was established from the list of optical density data readings. EBG was determined if two consecutive absorbance readings increased by >10% over 50minutes. Vials with cultures expressing EBG were counted as an event. Since this study aimed to determine *in-vitro* the efficacy of selected antibiotics at 1MIC and 10^3^MIC in suppressing EBG, this was evaluated by comparing EBG prevalence (rates) at 72 hours and time-to-event analysis between cultures. Differences in EBG rates in samples exposed to antibiotic concentrations of 1MIC and 10^3^MIC at 72 hours were analyzed with Fisher exact probability test. Time-to-event curves were constructed for each of the bacterial strains and their corresponding antibiotic treatments. The log-rank test was used to test the differences between the time-to-event curves in cultures exposed to 1MIC and 10^3^MIC.

To further evaluate the sensitivity of bacterial growth inhibition to antibiotic treatment, the differences in the timing of EBG were compared between the various antibiotic concentrations for each of the strains. Only cultures exhibiting EBG within 72 hours were included in this analysis. Variations in timing between the different antibiotic concentrations: 0MIC, 1MIC, 4MIC, and 10^3^MIC, were analyzed with either the Mann-Whitney test or the Kruskal-Wallis test. Data was analyzed with the aid of a dedicated software program (GraphPad Prism version 6.00 for Windows, GraphPad Software, La Jolla California USA). Proportions were approximated to the nearest decimals and P values to the nearest thousandth.

## Results

One hundred and twenty-five cultures were set up: *P*. *aeruginosa* incubated with gentamicin (n = 26); *P*. *aeruginosa* incubated with amikacin (n = 18); *E*. *coli* incubated with gentamicin (n = 28); *E*. *coli* incubated with amikacin (n = 20); and *S*. *aureus* incubated with vancomycin (n = 33). In six (4.8%) cultures, there were technical errors leading to premature termination without achieving either one of the endpoints set for this experiment (EBG, no EBG at 72 hours). The premature termination was caused by device technical failure or inappropriate interpretation of the growth pattern graph as EBG. Of these, one *S*. *aureus* culture incubated with 1MIC vancomycin and three *S*. *aureus* cultures incubated with 4MIC of vancomycin demonstrated non-EBG growth pattern as defined for this study. Two other *S*. *aureus* cultures incubated with 10^3^MIC of vancomycin showed no growth at 48 hours when the experiment was prematurely terminated. Since these six cultures did not achieve either one of the two endpoints set for this experiment, these were excluded from the contingency analysis, but were included in the time-to-event analysis.

Table 1 presents the effect of the various antibiotic concentrations on growth inhibition of the tested bacteria in 119 cultures achieving either EBG or no EBG at 72 hours. Bacteria not exposed to antibiotics (0MIC) developed EBG within few hours. Most of the bacteria exposed to 1MIC (32 of 33 experiments) developed EBG, usually within 24 hours. None of the 36 tested bacteria demonstrated EBG at antibiotic concentrations of 10^3^MIC.

**Table 1:** Proportion and timing of cultures exhibiting exponential bacterial growth (EBG).

Experiment	Cultures with EBG /Total	P value*	Median timing of EBG (IQR)	P value**
All				
0 MIC	19/19	<0.001	1.7 (1.7,1.7)	<0.001
1 MIC	32/33		16.7 (11.7,32.2)	
4 MIC	12/31		33.8 (22.6,50.4)	
1000 MIC	0/36			
*P*. *aeruginosa* with gentamicin				
0 MIC	4/4	<0.001	2.0 (1.7,2.4)	0.003
1 MIC	6/6		32.5 (15.5,58.1)	
4 MIC	2/8		62.0 (51.9,72.0)	
1000 MIC	0/8			
*P*. *aeruginosa* with amikacin				
0 MIC	3/3	0.015	1.7 (1.7,3.2)	0.057
1 MIC	4/5		29.5 (22.3,40.1)	
4 MIC	0/4			
1000 MIC	0/6			
*E*. *coli* with gentamicin				
0 MIC	4/4	<0.001	1.7 (1.7,1.7)	<0.001
1 MIC	8/8		13.6 (10.9,16.3)	
4 MIC	4/8		24.5 (22.6,37.2)	
1000 MIC	0/8			
*E*. *coli* with amikacin				
0 MIC	3/3	0.002	1.7 (1.7,1.7)	0.002
1 MIC	5/5		10.9 (9.7,12.4)	
4 MIC	2/6		22.9 (19.3,26.4)	
1000 MIC	0/6			
*S*. *aureus* with vancomycin				
0 MIC	5/5	<0.001	1.7 (1.7,2.0)	<0.001
1 MIC	9/9		23.3 (14.8,39.5)	
4 MIC	4/5		44.1 (24.6,62.2)	
1000 MIC	0/8			

* P values for differences in the proportion of bacterial culture growth between 1 MIC and 1000 MIC;

** P values for differences in the timing of bacterial culture growth; EBG–exponential bacterial growth.

Table 1 also includes information concerning the median timing to EBG if such an event was recorded within the 72 hours allotted for this experiment. Overall, median times increased when cultures were exposed to higher MIC. Interpretation of these results must take into account that cultures not resulting in EBG were not included in this analysis, and these were usually those exposed to 4MIC and 10^3^MIC. Since only a few data points were available for the evaluation of *P*. *aeruginosa* exposed to amikacin, differences in timing of EBG observed between cultures exposed to 0MIC and 1MIC did not reach statistical significance (p = 0.057).

**Fig 2:**
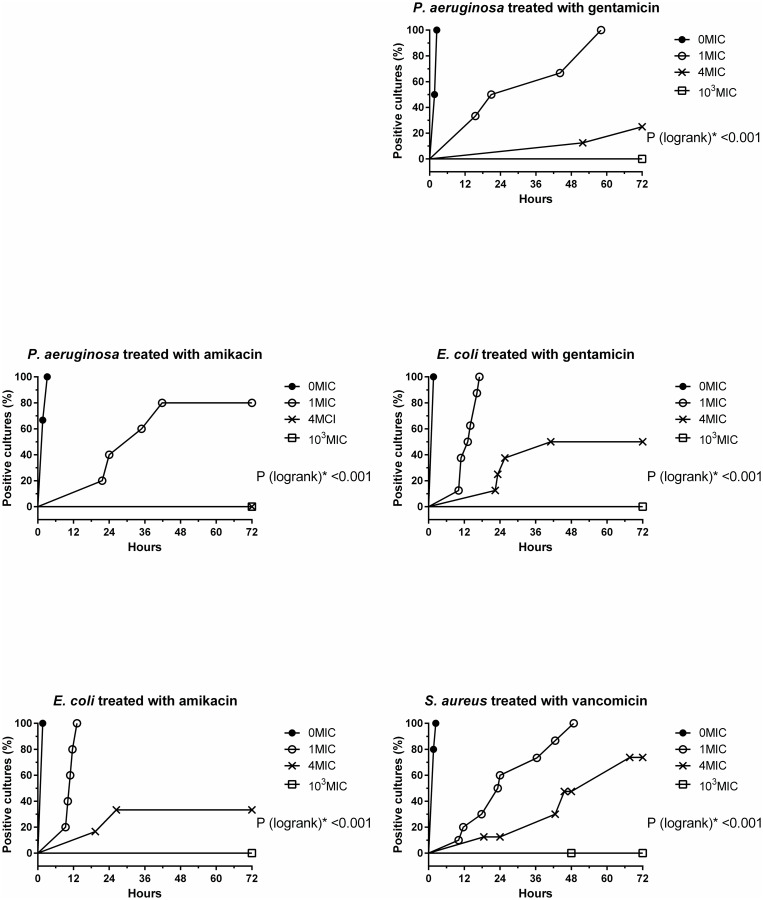
Percent of positive cultures following exposure to different antibiotic concentrations. *P (logrank) comparison between 1MIC and 10^3^MIC.

**Fig 3:**
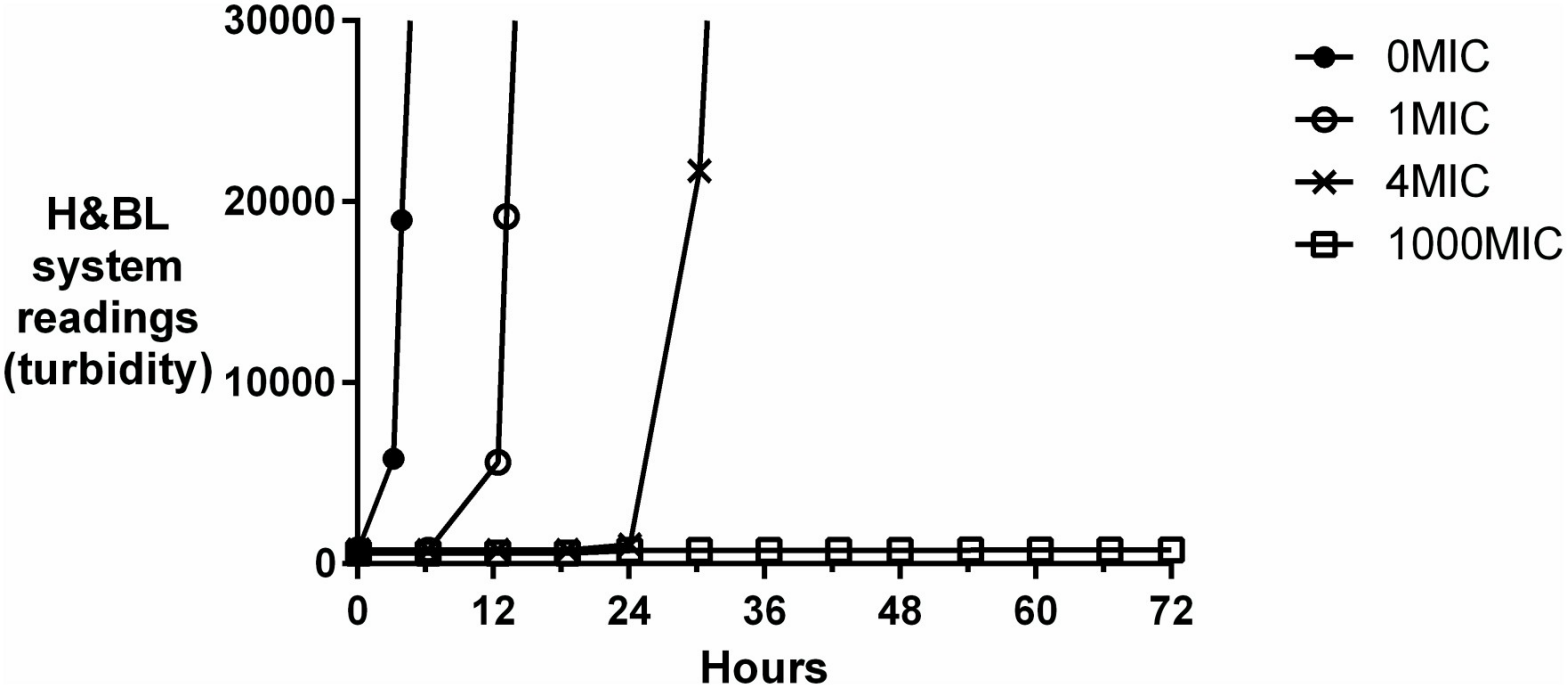
HB&L system readings over time in a single experiment set in which *E*. *coli* was exposed to increasing MICs of gentamicin, resulting in increased delay in exponential bacterial growth in 1MIC and 4MIC, while no growth was demonstrated at 10^3^MIC.

Fig 2 presents the time-to-event curves constructed for each of the bacterial strains and their corresponding antibiotic treatments provided at different MIC levels. Both EBG rates and latency period till EBG were related to the MIC level of the corresponding antibiotic. For example, when *P*. *aeruginosa* was cultured with gentamicin at 1 MIC, this delayed EBG onset compared to 0 MIC. However, all cultures eventually developed EBG. Increasing the gentamicin to 4MIC both decreased the rate of EBG and further delayed its onset. Gentamicin at 10^3^MIC, totally inhibited EBG in this experiment. Similar results were observed in the rest of the experiments. Fig 3 demonstrates a single experiment in which *E*. *coli* was exposed to different MICs of gentamicin. In this specific experiment, onset of EBG was delayed in inocula exposed to 1MIC and 4MIC compared to 0MIC. In the inocula exposed to 10^3^MIC, no EBG was observed.

## Discussion

In this in-vitro model, we show that 1MIC of gentamicin and amikacin only temporarily inhibited high bacterial loads of *E*. *coli* and *P*. *aeruginosa*, with a similar temporary effect demonstrated for treatment of *S*. *aureus* by vancomycin. CITA at 10^3^MIC of gentamicin and amikacin totally inhibited the growth of high bacterial loads of *E*. *coli* and *P*. *aeruginosa* throughout the entire 72 hours of the incubation period. CITA at 10^3^MIC of vancomycin had the same effect on *S*. *aureus*.

We applied CITA in the range of 10^2^–10^3^ MIC, as a simulation of our clinical experience treating infected CIEDs with expected biofilm bacterial growth over the hardware (as was observed in our clinical work). The minimum biofilm eradication concentration (MBEC) is within the 10^2^-10^3^MIC range. Our objective was to evaluate the maximum antibiotic dose, which would be both effective and safe in case of rapid drug absorption from the wound into the systemic circulation. It is for this reason that in the clinical scenario, we chose the recommended daily intravenous doses. These provide 10^2^-10^3^MIC of antibiotic concentration within the wound. Reducing the concentration may expose the pathogens to sub-inhibitory levels of antibiotics allowing the emergence of tolerance/resistant strains.

Increasing local antibiotic concentrations was found to be directly related, not only to decreased EBG occurrence but also to the increased latency period till EBG. Our findings reemphasize the need for high antibiotic concentration at the infection site for bacterial growth inhibition of high bacterial loads. The mechanism of EBG latency and inhibition with increased antibiotic concentrations should be further explored.

The maximal systemic antibiotic administration dose by either an intravenous or oral route is conventionally limited to systemic levels in the order of 1MIC depending on the antibiotic and clinical considerations (e.g., impaired renal function, organ toxicity). When high concentrations of antibiotics at the infection site are required, both the intravenous and the oral routes may be restricted since systemic toxic levels will be exceeded. Unlike the intravenous and oral antibiotic administration routes, the CITA method allows for the reversal of the concentration gradient, providing localized, 10^2^-10^3^MIC antibiotic levels at the infected cavity and 1MIC range serum levels, avoiding systemic toxicity [15].

Localized infections associated with implantable devices, osteomyelitis, and heavily infected wounds are at times life-threatening conditions, imposing a substantial risk for the patients and are associated with significant medical and financial burden on the global health systems [1, 19, 20]. The marked increased use of various permanent implantable devices, frequently in elderly patients with associated comorbidities, has been correlated with an increasing number of localized device infections [21]. Coronary artery bypass surgery complicated by deep sternal wound infection frequently involves osteomyelitis of the sternal bone [22]. Elderly and paraplegic patients are at higher risk of developing deep tissue pressure injuries (i.e., pressure ulcers) that are frequently complicated by osteomyelitis [23, 24]. As confirmed by our findings of the limited inhibition effect of 1MIC antibiotic levels, the efficacy of the traditional intravenous and oral antibiotic administration is often limited in these conditions due to the relatively low antibiotic concentrations that can be attained at the infection site. The high localized loads of bacteria, the incidence of osteomyelitis, foreign bodies (e.g., implantable devices) with biofilm growth, limit the efficacy of conventional, oral, or intravenous antibiotic administration. Thus treatment of these localized infections involves wide debridement, frequent need for removal of the implanted devices, and complex reconstructive surgery together with long periods of systemic antibiotic therapy.

**Fig 4:**
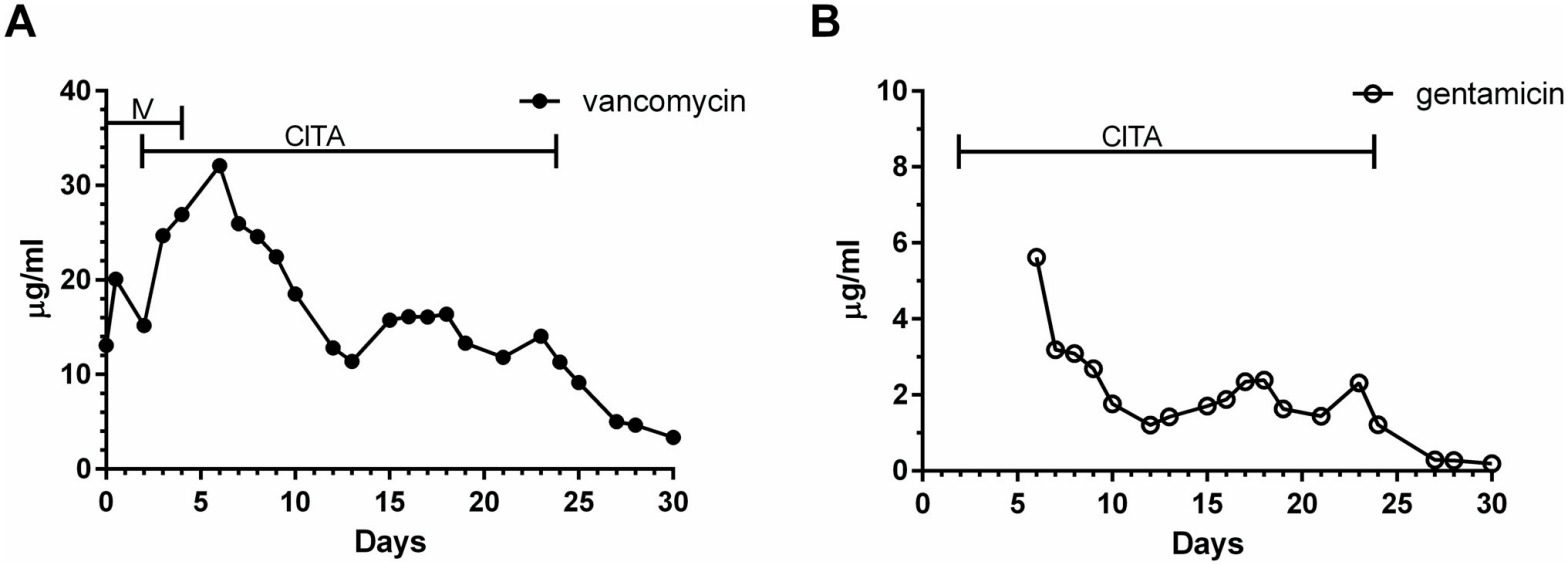
A patient with an infected knee following knee replacement was initially treated with intravenous vancomycin. CITA treatment started on the day of surgery (day 2), and intravenous therapy was stopped on day 4. CITA treatment was stopped on the 24th day. Cultures demonstrated methicillin-resistant *S*. *aureus* and *P*. *aeruginosa*.

Various methods of local antibiotic administration have been reported. Most literature published to date deals with prevention [25, 26]. Some studies published recently describe local antibiotic administration in treating established infections [27, 28]. In the early in-vivo experimental report of the clinical implementation of the CITA treatment method for localized infection of CIEDs, the antibiotics were provided at the infection site, proved effective, with a CIED salvage rate of over 80% [14]. We have recently updated our experience, further showing that CITA at 10^2^–10^3^MIC was safe and effective [15]. The senior author has applied the CITA method with similar results for compartmentalized deep sternal wound infection, deep tissue pressure injuries, heavily infected localized amputation wounds, and implanted joints infection (unpublished data). Serum levels of antibiotics in a representative clinical case treated with the CITA administration method are demonstrated in Fig 4. This patient presented with an infection following total knee replacement. Antibiotic serum levels of vancomycin (A) and gentamicin (B) are presented. Effective and safe vancomycin and gentamicin serum levels could be attained while achieving, ultra-high, 10^2^-10^3^MIC at the intraarticular cavity. A similar treatment pattern was observed in our patients treated for all the above indications of compartmentalized infections by CITA with vancomycin and gentamicin irrespective to tissue vascularity. Our current in-vitro findings provide early laboratory proof-of-concept justifying this clinical approach of introducing ultra-high concentrations of antibiotics to the infection site, enabling the inhibition of bacterial growth of even high levels of bacteria and localized biofilm.

Certain limitations should be considered when extrapolating the results of this study to clinical treatment. Differences exist between a compartmentalized infection in clinical situations and the environment within a vial containing a growth medium, as used in this experiment. Differences may include: pH, oxygen partial pressures, presence of proteins, enzymes, and the presence of foreign materials. All may potentially reduce the efficiency of antibiotics in preventing EBG. However, whatever these differences may be, the antimicrobial efficacy will still be related to the concentration of antibiotics achieved within the infected cavity. It is expected that substantially higher concentrations within the infected cavity achieved via CITA will perform more effectively than lower doses achieved by the conventional intravenous or the oral routes.

In the clinical CITA treatment, antibiotics are continuously administered, while in the *invitro* experiment, the antibiotics were added to the vial once. Continuous administration of antibiotics at 10^3^MIC could further delay EBG compared to a one-dose regimen, through the local buildup of antibiotic concentrations, with slow defusion release to the surrounding tissues. For example, continuous administration of vancomycin at the site of infection leads to drug accumulation. This was observed in clinical cases, where serum levels of vancomycin could still be measured 5–7 days after CITA was terminated (Fig 4).

The study was designed to evaluate EBG within 72 hours under different concentrations of antibiotics. Since inhibition of EBG does not directly imply bacterial eradication, theoretically, withdrawal of antibiotics following cessation of treatment could lead to renewed bacterial growth. Our experience with the successful treatment of multiple patients with infected CIEDs implies that in the majority of cases, complete bacterial killing can be achieved [14, 15].

Since the volume of the vials is constant, we were able to provide the antibiotics at predefined concentrations. In the clinical setting, dead space volumes vary, ranging from a few millilitres in CIEDs to 10–20 milliliters in sternal cavities. The relative effectivity of the CITA treatment is limited in the high volume compartments (e.g., abdominal cavity). Higher volume compartments require increased doses in order to attain local 10^2^–10^3^ MIC antibiotic levels. This involves administering antibiotics beyond the recommended daily dose with risk of inflicting systemic drug toxicity.

Ultra-high concentrations of antibiotics may potentially inhibit or alter wound healing. Our clinical experience demonstrates no adverse effect of 10^3^MIC of the applied in-situ antibiotics on wound healing. We already referenced our clinical experience in the treatment of over 80 infected CIEDs with no evidence of wound healing impairment. Another scenario includes patients with combat injuries of the extremity terated by CITA with immediate and early wound closure [16]. Clinical application of additional antibiotics in CITA treatment should be categorically evaluated. Previous application of the antibiotic by intramuscular applications, can serve as a reference for its safe use.

Though not the main objective of this experiment, the results of this study indicate that the H&BL system allows for the rapid determination of ineffective empirical antibiotic treatment irrespective of the identification of the drug-resistant pathogen causing the infection. Early adjustment of an appropriate antibiotic treatment thus can be achieved before pathogen identification and sensitivities are determined. Determination of drug sensitivity and the optimal antibiotic therapy for various microorganisms by the conventional, standard plate culture analysis are relatively slow and are not standardized for the 10^2^–10^3^ MIC CITA concentrations, which requires alternative laboratory tests and tooling. The H&BL system allows for detecting bacterial growth in a translucent culture medium within a relatively short period of time. The time latency for EBG can serve as an effective, early indicator for the sensitivity of the infective bacteria to a specific antibiotic. The same principle may apply for early detection of bacterial resistance to empirical treatment provided by the traditional intravenous and oral routes. Further studies to determine the efficacy of additional antibiotic agents, wider range of antibiotic concentrations, combination antibiotics effect, and the adequate exposure time required to reach both bacteriostatic and bacteriocidal effects is recommended. In addition, further evaluation of the value of the CITA approach in treating additional bacteria, polymicrobial infections and biofilm growth, are advocated.

## Conclusion

This research demonstrated the growth inhibition of different bacteria species by various antibiotics by means of in-situ administration of 10^2^-10^3^MIC levels in an *in-vitro* model simulating localized infection with high bacterial loads. These findings provide initial laboratory support and a proof-of-concept for the application of CITA as a novel approach for antibiotic treatment of compartmentalized bacterial infections.

## Supporting information

S1 File Experimental data set. (DOCX)
