# Supplementary material for: In-vitro model for bacterial growth inhibition of compartmentalized infection treated by an ultra-high concentration of antibiotics
Source: PLoS One. 2021 Jun 8;16(6):e0252724. doi: 10.1371/journal.pone.0252724 (PMC8186763; doi:10.1371/journal.pone.0252724)
Supplement: S1 File — (DOCX) [file pone.0252724.s001.docx]

Pseudomonas Garamycin control 1

| Time |  |
| --- | --- |
| 0h:05m | 520 |
| 0h:50m | 537 |
| 1h:40m | 643 |
| 2h:25m | 1138 |
| 3h:10m | 2732 |
| 3h:55m | 3662 |
| 4h:45m | 5058 |
| 5h:30m | 7575 |
| 6h:15m | 10615 |
| 7h:00m | 14859 |
| 7h:50m | 27810 |
| 8h:35m | 30538 |
| 9h:20m | 30897 |
| 10h:05m | 30898 |
| 10h:55m | 30901 |
| 11h:40m | 30902 |
| 12h:25m | 30902 |
| 13h:10m | 30903 |
| 14h:00m | 30903 |
| 14h:45m | 30903 |
| 15h:30m | 30903 |
| 16h:15m | 30904 |
| 17h:05m | 30905 |
| 17h:50m | 30904 |
| 18h:35m | 30903 |
| 19h:20m | 30905 |
| 20h:10m | 30904 |
| 20h:55m | 30906 |
| 21h:40m | 30905 |
| 22h:25m | 30908 |
| 23h:15m | 30903 |
| 24h:00m | 30977 |
| 24h:05m |  |
| 24h:50m |  |
| 25h:40m |  |
| 26h:25m |  |
| 27h:10m |  |
| 27h:55m |  |
| 28h:45m |  |
| 29h:30m |  |
| 30h:15m |  |
| 31h:00m |  |
| 31h:50m |  |
| 32h:35m |  |
| 33h:20m |  |
| 34h:05m |  |
| 34h:55m |  |
| 35h:40m |  |
| 36h:25m |  |
| 37h:10m |  |
| 38h:00m |  |
| 38h:45m |  |
| 39h:30m |  |
| 40h:15m |  |
| 41h:05m |  |
| 41h:50m |  |
| 42h:35m |  |
| 43h:20m |  |
| 44h:10m |  |
| 44h:55m |  |
| 45h:40m |  |
| 46h:25m |  |
| 47h:15m |  |
| 48h:00m |  |
| 48h:05m |  |
| 48h:50m |  |
| 49h:40m |  |
| 50h:25m |  |
| 51h:10m |  |
| 51h:55m |  |
| 52h:45m |  |
| 53h:30m |  |
| 54h:15m |  |
| 55h:00m |  |
| 55h:50m |  |
| 56h:35m |  |
| 57h:20m |  |
| 58h:05m |  |
| 58h:55m |  |
| 59h:40m |  |
| 60h:25m |  |
| 61h:10m |  |
| 62h:00m |  |
| 62h:45m |  |
| 63h:30m |  |
| 64h:15m |  |
| 65h:05m |  |
| 65h:50m |  |
| 66h:35m |  |
| 67h:20m |  |
| 68h:10m |  |
| 68h:55m |  |
| 69h:40m |  |
| 70h:25m |  |
| 71h:15m |  |
| 72h:00m |  |

Pseudomonas Garamycin control 2

| Time |  |
| --- | --- |
| 0h:05m | 991 |
| 0h:50m | 1001 |
| 1h:40m | 1109 |
| 2h:25m | 1571 |
| 3h:10m | 3238 |
| 3h:55m | 4157 |
| 4h:45m | 5651 |
| 5h:30m | 8142 |
| 6h:15m | 11553 |
| 7h:00m | 13669 |
| 7h:50m | 24317 |
| 8h:35m | 30897 |
| 9h:20m | 30985 |
| 10h:05m | 30983 |
| 10h:55m | 30982 |
| 11h:40m | 30979 |
| 12h:25m | 30977 |
| 13h:10m | 30976 |
| 14h:00m | 30971 |
| 14h:45m | 30971 |
| 15h:30m | 30968 |
| 16h:15m | 30968 |
| 17h:05m | 30967 |
| 17h:50m | 30967 |
| 18h:35m | 30961 |
| 19h:20m | 30959 |
| 20h:10m | 30987 |
| 20h:55m | 30988 |
| 21h:40m | 30990 |
| 22h:25m | 30990 |
| 23h:15m | 30991 |
| 24h:00m | 30988 |
| 24h:05m |  |
| 24h:50m |  |
| 25h:40m |  |
| 26h:25m |  |
| 27h:10m |  |
| 27h:55m |  |
| 28h:45m |  |
| 29h:30m |  |
| 30h:15m |  |
| 31h:00m |  |
| 31h:50m |  |
| 32h:35m |  |
| 33h:20m |  |
| 34h:05m |  |
| 34h:55m |  |
| 35h:40m |  |
| 36h:25m |  |
| 37h:10m |  |
| 38h:00m |  |
| 38h:45m |  |
| 39h:30m |  |
| 40h:15m |  |
| 41h:05m |  |
| 41h:50m |  |
| 42h:35m |  |
| 43h:20m |  |
| 44h:10m |  |
| 44h:55m |  |
| 45h:40m |  |
| 46h:25m |  |
| 47h:15m |  |
| 48h:00m |  |
| 48h:05m |  |
| 48h:50m |  |
| 49h:40m |  |
| 50h:25m |  |
| 51h:10m |  |
| 51h:55m |  |
| 52h:45m |  |
| 53h:30m |  |
| 54h:15m |  |
| 55h:00m |  |
| 55h:50m |  |
| 56h:35m |  |
| 57h:20m |  |
| 58h:05m |  |
| 58h:55m |  |
| 59h:40m |  |
| 60h:25m |  |
| 61h:10m |  |
| 62h:00m |  |
| 62h:45m |  |
| 63h:30m |  |
| 64h:15m |  |
| 65h:05m |  |
| 65h:50m |  |
| 66h:35m |  |
| 67h:20m |  |
| 68h:10m |  |
| 68h:55m |  |
| 69h:40m |  |
| 70h:25m |  |
| 71h:15m |  |
| 72h:00m |  |

Pseudomonas Garamycin control 3

| 00:05 | 501 |
| --- | --- |
| 02:25 | 934 |
| 04:45 | 4383 |
| 07:05 | 14224 |
| 09:20 | 30912 |
| 11:40 | 30988 |
| 14:00 | 30991 |
| 16:20 | 30991 |
| 18:40 | 30991 |
| 21:00 | 30992 |
| 23:15 | 30991 |
| 25:35 | 30987 |
| 27:55 | 30990 |
| 30:15 | 30988 |
| 32:35 | 30986 |
| 34:55 | 30988 |
| 37:10 | 30988 |
| 39:30 | 30990 |
| 41:50 | 30990 |
| 44:10 | 30991 |
| 46:30 | 30990 |
| 48:50 | 30990 |
| 51:05 | 30990 |
| 53:25 | 30990 |
| 55:45 | 30986 |
| 58:05 | 30906 |
| 60:25 | 30903 |
| 62:45 | 30902 |
| 65:00 | 30903 |
| 67:20 | 30903 |
| 69:40 | 30904 |
| 72:00 | 30904 |

Pseudomonas Garamycin control 4

| 00:05 | 742 |
| --- | --- |
| 02:25 | 1141 |
| 04:45 | 4907 |
| 07:05 | 13303 |
| 09:20 | 30988 |
| 11:40 | 30972 |
| 14:00 | 30974 |
| 16:20 | 30975 |
| 18:40 | 30973 |
| 21:00 | 30970 |
| 23:15 | 30961 |
| 25:35 | 30959 |
| 27:55 | 30909 |
| 30:15 | 30909 |
| 32:35 | 27969 |
| 34:55 | 26346 |
| 37:10 | 24950 |
| 39:30 | 24024 |
| 41:50 | 23233 |
| 44:10 | 22605 |
| 46:30 | 22036 |
| 48:50 | 21366 |
| 51:05 | 20618 |
| 53:25 | 19892 |
| 55:45 | 19498 |
| 58:05 | 20157 |
| 60:25 | 20588 |
| 62:45 | 21429 |
| 65:00 | 22006 |
| 67:20 | 22684 |
| 69:40 | 23434 |
| 72:00 | 24680 |

Pseudomonas Garamycin 1MIC 1

| 0h:05m | 730 |
| --- | --- |
| 0h:50m | 734 |
| 1h:40m | 734 |
| 2h:25m | 733 |
| 3h:10m | 731 |
| 3h:55m | 730 |
| 4h:45m | 726 |
| 5h:30m | 726 |
| 6h:15m | 723 |
| 7h:00m | 722 |
| 7h:50m | 720 |
| 8h:35m | 720 |
| 9h:20m | 720 |
| 10h:05m | 719 |
| 10h:55m | 719 |
| 11h:40m | 722 |
| 12h:25m | 726 |
| 13h:10m | 736 |
| 14h:00m | 759 |
| 14h:45m | 807 |
| 15h:30m | 933 |
| 16h:15m | 1251 |
| 17h:05m | 1983 |
| 17h:50m | 3429 |
| 18h:35m | 4008 |
| 19h:20m | 4755 |
| 20h:10m | 5708 |
| 20h:55m | 6880 |
| 21h:40m | 7920 |
| 22h:25m | 20409 |
| 23h:15m | 26545 |
| 24h:00m | 28563 |
| 24h:05m |  |
| 24h:50m |  |
| 25h:40m |  |
| 26h:25m |  |
| 27h:10m |  |
| 27h:55m |  |
| 28h:45m |  |
| 29h:30m |  |
| 30h:15m |  |
| 31h:00m |  |
| 31h:50m |  |
| 32h:35m |  |
| 33h:20m |  |
| 34h:05m |  |
| 34h:55m |  |
| 35h:40m |  |
| 36h:25m |  |
| 37h:10m |  |
| 38h:00m |  |
| 38h:45m |  |
| 39h:30m |  |
| 40h:15m |  |
| 41h:05m |  |
| 41h:50m |  |
| 42h:35m |  |
| 43h:20m |  |
| 44h:10m |  |
| 44h:55m |  |
| 45h:40m |  |
| 46h:25m |  |
| 47h:15m |  |
| 48h:00m |  |
| 48h:05m |  |
| 48h:50m |  |
| 49h:40m |  |
| 50h:25m |  |
| 51h:10m |  |
| 51h:55m |  |
| 52h:45m |  |
| 53h:30m |  |
| 54h:15m |  |
| 55h:00m |  |
| 55h:50m |  |
| 56h:35m |  |
| 57h:20m |  |
| 58h:05m |  |
| 58h:55m |  |
| 59h:40m |  |
| 60h:25m |  |
| 61h:10m |  |
| 62h:00m |  |
| 62h:45m |  |
| 63h:30m |  |
| 64h:15m |  |
| 65h:05m |  |
| 65h:50m |  |
| 66h:35m |  |
| 67h:20m |  |
| 68h:10m |  |
| 68h:55m |  |
| 69h:40m |  |
| 70h:25m |  |
| 71h:15m |  |
| 72h:00m |  |

Pseudomonas Garamycin 1MIC 2

| 0h:05m | 789 |
| --- | --- |
| 0h:50m | 783 |
| 1h:40m | 778 |
| 2h:25m | 780 |
| 3h:10m | 779 |
| 3h:55m | 776 |
| 4h:45m | 771 |
| 5h:30m | 769 |
| 6h:15m | 768 |
| 7h:00m | 766 |
| 7h:50m | 765 |
| 8h:35m | 768 |
| 9h:20m | 771 |
| 10h:05m | 780 |
| 10h:55m | 791 |
| 11h:40m | 803 |
| 12h:25m | 821 |
| 13h:10m | 846 |
| 14h:00m | 883 |
| 14h:45m | 953 |
| 15h:30m | 1115 |
| 16h:15m | 1464 |
| 17h:05m | 2534 |
| 17h:50m | 3783 |
| 18h:35m | 4387 |
| 19h:20m | 5340 |
| 20h:10m | 6708 |
| 20h:55m | 8320 |
| 21h:40m | 15177 |
| 22h:25m | 25908 |
| 23h:15m | 28417 |
| 24h:00m | 30893 |
| 24h:05m |  |
| 24h:50m |  |
| 25h:40m |  |
| 26h:25m |  |
| 27h:10m |  |
| 27h:55m |  |
| 28h:45m |  |
| 29h:30m |  |
| 30h:15m |  |
| 31h:00m |  |
| 31h:50m |  |
| 32h:35m |  |
| 33h:20m |  |
| 34h:05m |  |
| 34h:55m |  |
| 35h:40m |  |
| 36h:25m |  |
| 37h:10m |  |
| 38h:00m |  |
| 38h:45m |  |
| 39h:30m |  |
| 40h:15m |  |
| 41h:05m |  |
| 41h:50m |  |
| 42h:35m |  |
| 43h:20m |  |
| 44h:10m |  |
| 44h:55m |  |
| 45h:40m |  |
| 46h:25m |  |
| 47h:15m |  |
| 48h:00m |  |
| 48h:05m |  |
| 48h:50m |  |
| 49h:40m |  |
| 50h:25m |  |
| 51h:10m |  |
| 51h:55m |  |
| 52h:45m |  |
| 53h:30m |  |
| 54h:15m |  |
| 55h:00m |  |
| 55h:50m |  |
| 56h:35m |  |
| 57h:20m |  |
| 58h:05m |  |
| 58h:55m |  |
| 59h:40m |  |
| 60h:25m |  |
| 61h:10m |  |
| 62h:00m |  |
| 62h:45m |  |
| 63h:30m |  |
| 64h:15m |  |
| 65h:05m |  |
| 65h:50m |  |
| 66h:35m |  |
| 67h:20m |  |
| 68h:10m |  |
| 68h:55m |  |
| 69h:40m |  |
| 70h:25m |  |
| 71h:15m |  |
| 72h:00m |  |

Pseudomonas Garamycin 1MIC 3

| 00:05 | 524 |
| --- | --- |
| 02:25 | 536 |
| 04:45 | 543 |
| 07:05 | 544 |
| 09:20 | 547 |
| 11:40 | 548 |
| 14:00 | 549 |
| 16:20 | 551 |
| 18:40 | 548 |
| 21:00 | 551 |
| 23:15 | 550 |
| 25:35 | 552 |
| 27:55 | 553 |
| 30:15 | 555 |
| 32:35 | 557 |
| 34:55 | 558 |
| 37:10 | 558 |
| 39:30 | 559 |
| 41:50 | 562 |
| 44:10 | 561 |
| 46:30 | 564 |
| 48:50 | 569 |
| 51:05 | 575 |
| 53:25 | 596 |
| 55:45 | 688 |
| 58:05 | 1118 |
| 60:25 | 2523 |
| 62:45 | 3444 |
| 65:00 | 14413 |
| 67:20 | 21491 |
| 69:40 | 22837 |
| 72:00 | 24086 |

Pseudomonas Garamycin 1MIC 4

| 00:05 | 457 |
| --- | --- |
| 02:25 | 455 |
| 04:45 | 467 |
| 07:05 | 468 |
| 09:20 | 465 |
| 11:40 | 464 |
| 14:00 | 472 |
| 16:20 | 478 |
| 18:40 | 523 |
| 21:00 | 866 |
| 23:15 | 2532 |
| 25:35 | 3890 |
| 27:55 | 12246 |
| 30:15 | 26755 |
| 32:35 | 30658 |
| 34:55 | 30903 |
| 37:10 | 30906 |
| 39:30 | 30908 |
| 41:50 | 30977 |
| 44:10 | 30986 |
| 46:30 | 30987 |
| 48:50 | 30986 |
| 51:05 | 30979 |
| 53:25 | 30976 |
| 55:45 | 30974 |
| 58:05 | 30971 |
| 60:25 | 30972 |
| 62:45 | 30973 |
| 65:00 | 30975 |
| 67:20 | 30968 |
| 69:40 | 30966 |
| 72:00 | 30970 |

Pseudomonas Garamycin 1MIC 5

| 00:05 | 354 |
| --- | --- |
| 02:25 | 345 |
| 04:45 | 344 |
| 07:05 | 343 |
| 09:20 | 340 |
| 11:40 | 342 |
| 14:00 | 341 |
| 16:20 | 343 |
| 18:40 | 348 |
| 21:00 | 364 |
| 23:15 | 385 |
| 25:35 | 397 |
| 27:55 | 405 |
| 30:15 | 417 |
| 32:35 | 437 |
| 34:55 | 461 |
| 37:10 | 484 |
| 39:30 | 519 |
| 41:50 | 592 |
| 44:10 | 1096 |
| 46:30 | 2854 |
| 48:50 | 7353 |
| 51:05 | 19205 |
| 53:25 | 22192 |
| 55:45 | 22667 |
| 58:05 | 23040 |
| 60:25 | 23230 |
| 62:45 | 23622 |
| 65:00 | 24167 |
| 67:20 | 23849 |
| 69:40 | 23830 |
| 72:00 | 23843 |

Pseudomonas Garamycin 1MIC 6

| 00:05 | 351 |
| --- | --- |
| 02:25 | 340 |
| 04:45 | 337 |
| 07:05 | 336 |
| 09:20 | 334 |
| 11:40 | 334 |
| 14:00 | 334 |
| 16:20 | 340 |
| 18:40 | 351 |
| 21:00 | 360 |
| 23:15 | 366 |
| 25:35 | 371 |
| 27:55 | 378 |
| 30:15 | 383 |
| 32:35 | 387 |
| 34:55 | 395 |
| 37:10 | 405 |
| 39:30 | 440 |
| 41:50 | 485 |
| 44:10 | 589 |
| 46:30 | 667 |
| 48:50 | 753 |
| 51:05 | 844 |
| 53:25 | 962 |
| 55:45 | 1177 |
| 58:05 | 1765 |
| 60:25 | 2958 |
| 62:45 | 3761 |
| 65:00 | 9675 |
| 67:20 | 16170 |
| 69:40 | 18262 |
| 72:00 | 18505 |

Pseudomonas Garamycin 4MIC 1

| 0h:05m | 667 |
| --- | --- |
| 0h:50m | 665 |
| 1h:40m | 665 |
| 2h:25m | 662 |
| 3h:10m | 663 |
| 3h:55m | 658 |
| 4h:45m | 657 |
| 5h:30m | 656 |
| 6h:15m | 655 |
| 7h:00m | 654 |
| 7h:50m | 655 |
| 8h:35m | 653 |
| 9h:20m | 653 |
| 10h:05m | 653 |
| 10h:55m | 650 |
| 11h:40m | 651 |
| 12h:25m | 652 |
| 13h:10m | 654 |
| 14h:00m | 655 |
| 14h:45m | 655 |
| 15h:30m | 653 |
| 16h:15m | 655 |
| 17h:05m | 657 |
| 17h:50m | 656 |
| 18h:35m | 654 |
| 19h:20m | 658 |
| 20h:10m | 657 |
| 20h:55m | 659 |
| 21h:40m | 660 |
| 22h:25m | 659 |
| 23h:15m | 661 |
| 24h:00m | 665 |
| 24h:05m | 551 |
| 24h:50m | 554 |
| 25h:40m | 557 |
| 26h:25m | 554 |
| 27h:10m | 556 |
| 27h:55m | 560 |
| 28h:45m | 561 |
| 29h:30m | 560 |
| 30h:15m | 560 |
| 31h:00m | 562 |
| 31h:50m | 563 |
| 32h:35m | 563 |
| 33h:20m | 562 |
| 34h:05m | 562 |
| 34h:55m | 562 |
| 35h:40m | 562 |
| 36h:25m | 560 |
| 37h:10m | 562 |
| 38h:00m | 566 |
| 38h:45m | 568 |
| 39h:30m | 567 |
| 40h:15m | 570 |
| 41h:05m | 571 |
| 41h:50m | 568 |
| 42h:35m | 571 |
| 43h:20m | 571 |
| 44h:10m | 573 |
| 44h:55m | 574 |
| 45h:40m | 579 |
| 46h:25m | 581 |
| 47h:15m | 585 |
| 48h:00m | 590 |
| 48h:05m | 906 |
| 48h:50m | 949 |
| 49h:40m | 1010 |
| 50h:25m | 1078 |
| 51h:10m | 1161 |
| 51h:55m | 1280 |
| 52h:45m | 1471 |
| 53h:30m | 1776 |
| 54h:15m | 2529 |
| 55h:00m | 2895 |
| 55h:50m | 3278 |
| 56h:35m | 3716 |
| 57h:20m | 4389 |
| 58h:05m | 5454 |
| 58h:55m | 10859 |
| 59h:40m | 15717 |
| 60h:25m | 17351 |
| 61h:10m | 18716 |
| 62h:00m | 19026 |
| 62h:45m | 20312 |
| 63h:30m | 20678 |
| 64h:15m | 20798 |
| 65h:05m | 20945 |
| 65h:50m | 21021 |
| 66h:35m | 21027 |
| 67h:20m | 21109 |
| 68h:10m | 21196 |
| 68h:55m | 21639 |
| 69h:40m | 21420 |
| 70h:25m | 21312 |
| 71h:15m | 21640 |
| 72h:00m | 21707 |

Pseudomonas Garamycin 4MIC 2

| 0h:05m | 816 |
| --- | --- |
| 0h:50m | 812 |
| 1h:40m | 810 |
| 2h:25m | 809 |
| 3h:10m | 810 |
| 3h:55m | 812 |
| 4h:45m | 807 |
| 5h:30m | 806 |
| 6h:15m | 808 |
| 7h:00m | 803 |
| 7h:50m | 804 |
| 8h:35m | 803 |
| 9h:20m | 801 |
| 10h:05m | 801 |
| 10h:55m | 801 |
| 11h:40m | 798 |
| 12h:25m | 800 |
| 13h:10m | 798 |
| 14h:00m | 799 |
| 14h:45m | 798 |
| 15h:30m | 797 |
| 16h:15m | 800 |
| 17h:05m | 799 |
| 17h:50m | 803 |
| 18h:35m | 803 |
| 19h:20m | 806 |
| 20h:10m | 807 |
| 20h:55m | 811 |
| 21h:40m | 814 |
| 22h:25m | 816 |
| 23h:15m | 817 |
| 24h:00m | 822 |
| 24h:05m | 615 |
| 24h:50m | 621 |
| 25h:40m | 624 |
| 26h:25m | 623 |
| 27h:10m | 625 |
| 27h:55m | 625 |
| 28h:45m | 624 |
| 29h:30m | 627 |
| 30h:15m | 627 |
| 31h:00m | 627 |
| 31h:50m | 627 |
| 32h:35m | 628 |
| 33h:20m | 627 |
| 34h:05m | 630 |
| 34h:55m | 627 |
| 35h:40m | 627 |
| 36h:25m | 628 |
| 37h:10m | 629 |
| 38h:00m | 632 |
| 38h:45m | 634 |
| 39h:30m | 633 |
| 40h:15m | 636 |
| 41h:05m | 636 |
| 41h:50m | 635 |
| 42h:35m | 638 |
| 43h:20m | 638 |
| 44h:10m | 637 |
| 44h:55m | 639 |
| 45h:40m | 640 |
| 46h:25m | 643 |
| 47h:15m | 639 |
| 48h:00m | 642 |
| 48h:05m | 905 |
| 48h:50m | 910 |
| 49h:40m | 934 |
| 50h:25m | 939 |
| 51h:10m | 945 |
| 51h:55m | 942 |
| 52h:45m | 949 |
| 53h:30m | 954 |
| 54h:15m | 946 |
| 55h:00m | 943 |
| 55h:50m | 950 |
| 56h:35m | 951 |
| 57h:20m | 949 |
| 58h:05m | 952 |
| 58h:55m | 955 |
| 59h:40m | 953 |
| 60h:25m | 959 |
| 61h:10m | 963 |
| 62h:00m | 965 |
| 62h:45m | 968 |
| 63h:30m | 973 |
| 64h:15m | 988 |
| 65h:05m | 1001 |
| 65h:50m | 1018 |
| 66h:35m | 1028 |
| 67h:20m | 1051 |
| 68h:10m | 1073 |
| 68h:55m | 1103 |
| 69h:40m | 1148 |
| 70h:25m | 1183 |
| 71h:15m | 1249 |
| 72h:00m | 1309 |

Pseudomonas Garamycin 4MIC 3

| 0h:05m | 854 |
| --- | --- |
| 0h:50m | 849 |
| 1h:40m | 852 |
| 2h:25m | 853 |
| 3h:10m | 844 |
| 3h:55m | 846 |
| 4h:45m | 854 |
| 5h:30m | 841 |
| 6h:15m | 854 |
| 7h:00m | 844 |
| 7h:50m | 853 |
| 8h:35m | 834 |
| 9h:20m | 839 |
| 10h:05m | 838 |
| 10h:55m | 831 |
| 11h:40m | 831 |
| 12h:25m | 830 |
| 13h:10m | 831 |
| 14h:00m | 835 |
| 14h:45m | 832 |
| 15h:30m | 842 |
| 16h:15m | 836 |
| 17h:05m | 841 |
| 17h:50m | 841 |
| 18h:35m | 838 |
| 19h:20m | 845 |
| 20h:10m | 842 |
| 20h:55m | 855 |
| 21h:40m | 850 |
| 22h:25m | 843 |
| 23h:15m | 850 |
| 24h:00m | 861 |
| 24h:05m | 817 |
| 24h:50m | 834 |
| 25h:40m | 820 |
| 26h:25m | 816 |
| 27h:10m | 826 |
| 27h:55m | 828 |
| 28h:45m | 815 |
| 29h:30m | 815 |
| 30h:15m | 811 |
| 31h:00m | 807 |
| 31h:50m | 813 |
| 32h:35m | 807 |
| 33h:20m | 813 |
| 34h:05m | 813 |
| 34h:55m | 807 |
| 35h:40m | 815 |
| 36h:25m | 816 |
| 37h:10m | 798 |
| 38h:00m | 825 |
| 38h:45m | 813 |
| 39h:30m | 811 |
| 40h:15m | 825 |
| 41h:05m | 818 |
| 41h:50m | 835 |
| 42h:35m | 815 |
| 43h:20m | 828 |
| 44h:10m | 824 |
| 44h:55m | 825 |
| 45h:40m | 849 |
| 46h:25m | 846 |
| 47h:15m | 869 |
| 48h:00m | 864 |
| 48h:05m | 763 |
| 48h:50m | 750 |
| 49h:40m | 752 |
| 50h:25m | 755 |
| 51h:10m | 774 |
| 51h:55m | 795 |
| 52h:45m | 810 |
| 53h:30m | 810 |
| 54h:15m | 788 |
| 55h:00m | 803 |
| 55h:50m | 807 |
| 56h:35m | 803 |
| 57h:20m | 825 |
| 58h:05m | 834 |
| 58h:55m | 849 |
| 59h:40m | 827 |
| 60h:25m | 842 |
| 61h:10m | 860 |
| 62h:00m | 839 |
| 62h:45m | 848 |
| 63h:30m | 879 |
| 64h:15m | 891 |
| 65h:05m | 906 |
| 65h:50m | 916 |
| 66h:35m | 943 |
| 67h:20m | 928 |
| 68h:10m | 936 |
| 68h:55m | 922 |
| 69h:40m | 958 |
| 70h:25m | 972 |
| 71h:15m | 989 |
| 72h:00m | 986 |

Pseudomonas Garamycin 4MIC 4

| 0h:05m | 1265 |
| --- | --- |
| 0h:50m | 1263 |
| 1h:40m | 1250 |
| 2h:25m | 1242 |
| 3h:10m | 1236 |
| 3h:55m | 1235 |
| 4h:45m | 1232 |
| 5h:30m | 1231 |
| 6h:15m | 1228 |
| 7h:00m | 1232 |
| 7h:50m | 1228 |
| 8h:35m | 1224 |
| 9h:20m | 1223 |
| 10h:05m | 1226 |
| 10h:55m | 1228 |
| 11h:40m | 1227 |
| 12h:25m | 1228 |
| 13h:10m | 1227 |
| 14h:00m | 1229 |
| 14h:45m | 1231 |
| 15h:30m | 1237 |
| 16h:15m | 1252 |
| 17h:05m | 1255 |
| 17h:50m | 1262 |
| 18h:35m | 1274 |
| 19h:20m | 1282 |
| 20h:10m | 1296 |
| 20h:55m | 1295 |
| 21h:40m | 1304 |
| 22h:25m | 1311 |
| 23h:15m | 1317 |
| 24h:00m | 1312 |
| 24h:05m | 954 |
| 24h:50m | 955 |
| 25h:40m | 956 |
| 26h:25m | 958 |
| 27h:10m | 958 |
| 27h:55m | 956 |
| 28h:45m | 959 |
| 29h:30m | 959 |
| 30h:15m | 957 |
| 31h:00m | 957 |
| 31h:50m | 960 |
| 32h:35m | 961 |
| 33h:20m | 955 |
| 34h:05m | 953 |
| 34h:55m | 954 |
| 35h:40m | 959 |
| 36h:25m | 958 |
| 37h:10m | 957 |
| 38h:00m | 960 |
| 38h:45m | 957 |
| 39h:30m | 953 |
| 40h:15m | 955 |
| 41h:05m | 955 |
| 41h:50m | 960 |
| 42h:35m | 956 |
| 43h:20m | 963 |
| 44h:10m | 966 |
| 44h:55m | 964 |
| 45h:40m | 969 |
| 46h:25m | 971 |
| 47h:15m | 971 |
| 48h:00m | 979 |
| 48h:05m | 730 |
| 48h:50m | 731 |
| 49h:40m | 731 |
| 50h:25m | 731 |
| 51h:10m | 734 |
| 51h:55m | 740 |
| 52h:45m | 742 |
| 53h:30m | 738 |
| 54h:15m | 743 |
| 55h:00m | 743 |
| 55h:50m | 745 |
| 56h:35m | 743 |
| 57h:20m | 745 |
| 58h:05m | 745 |
| 58h:55m | 744 |
| 59h:40m | 745 |
| 60h:25m | 750 |
| 61h:10m | 747 |
| 62h:00m | 747 |
| 62h:45m | 748 |
| 63h:30m | 750 |
| 64h:15m | 751 |
| 65h:05m | 753 |
| 65h:50m | 754 |
| 66h:35m | 755 |
| 67h:20m | 751 |
| 68h:10m | 751 |
| 68h:55m | 753 |
| 69h:40m | 754 |
| 70h:25m | 756 |
| 71h:15m | 755 |
| 72h:00m | 758 |

Pseudomonas Garamycin 4MIC 5

| 00:05 | 411 |
| --- | --- |
| 02:25 | 416 |
| 04:45 | 415 |
| 07:05 | 413 |
| 09:20 | 410 |
| 11:40 | 406 |
| 14:00 | 406 |
| 16:20 | 405 |
| 18:40 | 404 |
| 21:00 | 404 |
| 23:15 | 403 |
| 25:35 | 405 |
| 27:55 | 406 |
| 30:15 | 410 |
| 32:35 | 411 |
| 34:55 | 412 |
| 37:10 | 412 |
| 39:30 | 413 |
| 41:50 | 413 |
| 44:10 | 415 |
| 46:30 | 415 |
| 48:50 | 416 |
| 51:05 | 418 |
| 53:25 | 420 |
| 55:45 | 423 |
| 58:05 | 425 |
| 60:25 | 427 |
| 62:45 | 432 |
| 65:00 | 433 |
| 67:20 | 436 |
| 69:40 | 441 |
| 72:00 | 444 |

Pseudomonas Garamycin 4MIC 6

| 00:05 | 353 |
| --- | --- |
| 02:25 | 343 |
| 04:45 | 345 |
| 07:05 | 346 |
| 09:20 | 345 |
| 11:40 | 345 |
| 14:00 | 345 |
| 16:20 | 344 |
| 18:40 | 349 |
| 21:00 | 351 |
| 23:15 | 359 |
| 25:35 | 360 |
| 27:55 | 366 |
| 30:15 | 371 |
| 32:35 | 376 |
| 34:55 | 378 |
| 37:10 | 380 |
| 39:30 | 383 |
| 41:50 | 385 |
| 44:10 | 386 |
| 46:30 | 390 |
| 48:50 | 396 |
| 51:05 | 403 |
| 53:25 | 413 |
| 55:45 | 421 |
| 58:05 | 430 |
| 60:25 | 454 |
| 62:45 | 480 |
| 65:00 | 528 |
| 67:20 | 599 |
| 69:40 | 691 |
| 72:00 | 862 |

Pseudomonas Garamycin 4MIC 7

| 00:05 | 306 |
| --- | --- |
| 02:25 | 304 |
| 04:45 | 300 |
| 07:05 | 298 |
| 09:20 | 293 |
| 11:40 | 290 |
| 14:00 | 289 |
| 16:20 | 290 |
| 18:40 | 289 |
| 21:00 | 289 |
| 23:15 | 289 |
| 25:35 | 290 |
| 27:55 | 290 |
| 30:15 | 290 |
| 32:35 | 290 |
| 34:55 | 290 |
| 37:10 | 292 |
| 39:30 | 291 |
| 41:50 | 293 |
| 44:10 | 291 |
| 46:30 | 294 |
| 48:50 | 297 |
| 51:05 | 299 |
| 53:25 | 302 |
| 55:45 | 305 |
| 58:05 | 310 |
| 60:25 | 313 |
| 62:45 | 320 |
| 65:00 | 339 |
| 67:20 | 349 |
| 69:40 | 358 |
| 72:00 | 367 |

Pseudomonas Garamycin 4MIC 8

| 00:05 | 357 |
| --- | --- |
| 02:25 | 331 |
| 04:45 | 328 |
| 07:05 | 327 |
| 09:20 | 326 |
| 11:40 | 324 |
| 14:00 | 324 |
| 16:20 | 320 |
| 18:40 | 322 |
| 21:00 | 322 |
| 23:15 | 325 |
| 25:35 | 325 |
| 27:55 | 327 |
| 30:15 | 328 |
| 32:35 | 326 |
| 34:55 | 328 |
| 37:10 | 328 |
| 39:30 | 329 |
| 41:50 | 330 |
| 44:10 | 328 |
| 46:30 | 329 |
| 48:50 | 330 |
| 51:05 | 330 |
| 53:25 | 331 |
| 55:45 | 332 |
| 58:05 | 333 |
| 60:25 | 332 |
| 62:45 | 333 |
| 65:00 | 333 |
| 67:20 | 335 |
| 69:40 | 336 |
| 72:00 | 339 |

Pseudomonas Garamycin 1000MIC 1

| 0h:05m | 610 |
| --- | --- |
| 0h:50m | 620 |
| 1h:40m | 616 |
| 2h:25m | 616 |
| 3h:10m | 617 |
| 3h:55m | 619 |
| 4h:45m | 620 |
| 5h:30m | 622 |
| 6h:15m | 625 |
| 7h:00m | 620 |
| 7h:50m | 623 |
| 8h:35m | 622 |
| 9h:20m | 630 |
| 10h:05m | 627 |
| 10h:55m | 626 |
| 11h:40m | 629 |
| 12h:25m | 624 |
| 13h:10m | 626 |
| 14h:00m | 623 |
| 14h:45m | 628 |
| 15h:30m | 625 |
| 16h:15m | 625 |
| 17h:05m | 627 |
| 17h:50m | 629 |
| 18h:35m | 627 |
| 19h:20m | 627 |
| 20h:10m | 630 |
| 20h:55m | 635 |
| 21h:40m | 630 |
| 22h:25m | 627 |
| 23h:15m | 631 |
| 24h:00m | 630 |
| 24h:05m | 623 |
| 24h:50m | 634 |
| 25h:40m | 638 |
| 26h:25m | 636 |
| 27h:10m | 642 |
| 27h:55m | 641 |
| 28h:45m | 644 |
| 29h:30m | 645 |
| 30h:15m | 645 |
| 31h:00m | 646 |
| 31h:50m | 646 |
| 32h:35m | 644 |
| 33h:20m | 648 |
| 34h:05m | 648 |
| 34h:55m | 648 |
| 35h:40m | 646 |
| 36h:25m | 647 |
| 37h:10m | 650 |
| 38h:00m | 649 |
| 38h:45m | 652 |
| 39h:30m | 653 |
| 40h:15m | 653 |
| 41h:05m | 658 |
| 41h:50m | 654 |
| 42h:35m | 659 |
| 43h:20m | 657 |
| 44h:10m | 657 |
| 44h:55m | 655 |
| 45h:40m | 655 |
| 46h:25m | 656 |
| 47h:15m | 658 |
| 48h:00m | 661 |
| 48h:05m | 776 |
| 48h:50m | 775 |
| 49h:40m | 773 |
| 50h:25m | 772 |
| 51h:10m | 771 |
| 51h:55m | 772 |
| 52h:45m | 772 |
| 53h:30m | 770 |
| 54h:15m | 775 |
| 55h:00m | 779 |
| 55h:50m | 774 |
| 56h:35m | 773 |
| 57h:20m | 775 |
| 58h:05m | 775 |
| 58h:55m | 774 |
| 59h:40m | 775 |
| 60h:25m | 779 |
| 61h:10m | 775 |
| 62h:00m | 777 |
| 62h:45m | 775 |
| 63h:30m | 775 |
| 64h:15m | 777 |
| 65h:05m | 776 |
| 65h:50m | 774 |
| 66h:35m | 776 |
| 67h:20m | 777 |
| 68h:10m | 773 |
| 68h:55m | 774 |
| 69h:40m | 777 |
| 70h:25m | 775 |
| 71h:15m | 772 |
| 72h:00m | 774 |

Pseudomonas Garamycin 1000MIC 2

| 0h:05m | 814 |
| --- | --- |
| 0h:50m | 813 |
| 1h:40m | 811 |
| 2h:25m | 808 |
| 3h:10m | 810 |
| 3h:55m | 806 |
| 4h:45m | 811 |
| 5h:30m | 806 |
| 6h:15m | 810 |
| 7h:00m | 808 |
| 7h:50m | 809 |
| 8h:35m | 810 |
| 9h:20m | 813 |
| 10h:05m | 813 |
| 10h:55m | 812 |
| 11h:40m | 815 |
| 12h:25m | 815 |
| 13h:10m | 814 |
| 14h:00m | 816 |
| 14h:45m | 814 |
| 15h:30m | 815 |
| 16h:15m | 814 |
| 17h:05m | 816 |
| 17h:50m | 815 |
| 18h:35m | 818 |
| 19h:20m | 817 |
| 20h:10m | 820 |
| 20h:55m | 820 |
| 21h:40m | 815 |
| 22h:25m | 816 |
| 23h:15m | 816 |
| 24h:00m | 819 |
| 24h:05m | 819 |
| 24h:50m | 821 |
| 25h:40m | 822 |
| 26h:25m | 824 |
| 27h:10m | 821 |
| 27h:55m | 821 |
| 28h:45m | 825 |
| 29h:30m | 823 |
| 30h:15m | 825 |
| 31h:00m | 826 |
| 31h:50m | 825 |
| 32h:35m | 829 |
| 33h:20m | 827 |
| 34h:05m | 827 |
| 34h:55m | 825 |
| 35h:40m | 823 |
| 36h:25m | 824 |
| 37h:10m | 822 |
| 38h:00m | 828 |
| 38h:45m | 824 |
| 39h:30m | 826 |
| 40h:15m | 824 |
| 41h:05m | 824 |
| 41h:50m | 824 |
| 42h:35m | 820 |
| 43h:20m | 820 |
| 44h:10m | 821 |
| 44h:55m | 824 |
| 45h:40m | 824 |
| 46h:25m | 825 |
| 47h:15m | 823 |
| 48h:00m | 829 |
| 48h:05m | 946 |
| 48h:50m | 952 |
| 49h:40m | 951 |
| 50h:25m | 950 |
| 51h:10m | 948 |
| 51h:55m | 951 |
| 52h:45m | 953 |
| 53h:30m | 952 |
| 54h:15m | 953 |
| 55h:00m | 952 |
| 55h:50m | 950 |
| 56h:35m | 953 |
| 57h:20m | 949 |
| 58h:05m | 953 |
| 58h:55m | 949 |
| 59h:40m | 954 |
| 60h:25m | 954 |
| 61h:10m | 955 |
| 62h:00m | 956 |
| 62h:45m | 952 |
| 63h:30m | 952 |
| 64h:15m | 956 |
| 65h:05m | 953 |
| 65h:50m | 951 |
| 66h:35m | 951 |
| 67h:20m | 951 |
| 68h:10m | 949 |
| 68h:55m | 951 |
| 69h:40m | 952 |
| 70h:25m | 951 |
| 71h:15m | 953 |
| 72h:00m | 954 |

Pseudomonas Garamycin 1000MIC 3

| 0h:05m | 675 |
| --- | --- |
| 0h:50m | 682 |
| 1h:40m | 682 |
| 2h:25m | 677 |
| 3h:10m | 680 |
| 3h:55m | 683 |
| 4h:45m | 681 |
| 5h:30m | 682 |
| 6h:15m | 683 |
| 7h:00m | 683 |
| 7h:50m | 682 |
| 8h:35m | 678 |
| 9h:20m | 681 |
| 10h:05m | 679 |
| 10h:55m | 681 |
| 11h:40m | 681 |
| 12h:25m | 680 |
| 13h:10m | 679 |
| 14h:00m | 677 |
| 14h:45m | 680 |
| 15h:30m | 680 |
| 16h:15m | 680 |
| 17h:05m | 679 |
| 17h:50m | 679 |
| 18h:35m | 677 |
| 19h:20m | 676 |
| 20h:10m | 678 |
| 20h:55m | 674 |
| 21h:40m | 675 |
| 22h:25m | 675 |
| 23h:15m | 677 |
| 24h:00m | 681 |
| 24h:05m | 566 |
| 24h:50m | 563 |
| 25h:40m | 564 |
| 26h:25m | 563 |
| 27h:10m | 566 |
| 27h:55m | 565 |
| 28h:45m | 561 |
| 29h:30m | 563 |
| 30h:15m | 560 |
| 31h:00m | 562 |
| 31h:50m | 560 |
| 32h:35m | 560 |
| 33h:20m | 562 |
| 34h:05m | 558 |
| 34h:55m | 560 |
| 35h:40m | 560 |
| 36h:25m | 562 |
| 37h:10m | 559 |
| 38h:00m | 560 |
| 38h:45m | 563 |
| 39h:30m | 561 |
| 40h:15m | 563 |
| 41h:05m | 557 |
| 41h:50m | 561 |
| 42h:35m | 560 |
| 43h:20m | 560 |
| 44h:10m | 559 |
| 44h:55m | 559 |
| 45h:40m | 560 |
| 46h:25m | 558 |
| 47h:15m | 557 |
| 48h:00m | 558 |
| 48h:05m | 557 |
| 48h:50m | 555 |
| 49h:40m | 555 |
| 50h:25m | 554 |
| 51h:10m | 557 |
| 51h:55m | 557 |
| 52h:45m | 557 |
| 53h:30m | 557 |
| 54h:15m | 559 |
| 55h:00m | 559 |
| 55h:50m | 559 |
| 56h:35m | 558 |
| 57h:20m | 558 |
| 58h:05m | 555 |
| 58h:55m | 557 |
| 59h:40m | 555 |
| 60h:25m | 557 |
| 61h:10m | 557 |
| 62h:00m | 555 |
| 62h:45m | 555 |
| 63h:30m | 557 |
| 64h:15m | 555 |
| 65h:05m | 558 |
| 65h:50m | 560 |
| 66h:35m | 559 |
| 67h:20m | 559 |
| 68h:10m | 558 |
| 68h:55m | 558 |
| 69h:40m | 558 |
| 70h:25m | 556 |
| 71h:15m | 558 |
| 72h:00m | 560 |

Pseudomonas Garamycin 1000MIC 4

| 0h:05m | 697 |
| --- | --- |
| 0h:50m | 694 |
| 1h:40m | 693 |
| 2h:25m | 695 |
| 3h:10m | 694 |
| 3h:55m | 695 |
| 4h:45m | 695 |
| 5h:30m | 695 |
| 6h:15m | 695 |
| 7h:00m | 695 |
| 7h:50m | 693 |
| 8h:35m | 693 |
| 9h:20m | 694 |
| 10h:05m | 694 |
| 10h:55m | 695 |
| 11h:40m | 696 |
| 12h:25m | 695 |
| 13h:10m | 694 |
| 14h:00m | 696 |
| 14h:45m | 694 |
| 15h:30m | 697 |
| 16h:15m | 698 |
| 17h:05m | 699 |
| 17h:50m | 697 |
| 18h:35m | 697 |
| 19h:20m | 698 |
| 20h:10m | 697 |
| 20h:55m | 696 |
| 21h:40m | 697 |
| 22h:25m | 696 |
| 23h:15m | 698 |
| 24h:00m | 698 |
| 24h:05m | 705 |
| 24h:50m | 704 |
| 25h:40m | 707 |
| 26h:25m | 708 |
| 27h:10m | 707 |
| 27h:55m | 707 |
| 28h:45m | 706 |
| 29h:30m | 707 |
| 30h:15m | 707 |
| 31h:00m | 707 |
| 31h:50m | 708 |
| 32h:35m | 706 |
| 33h:20m | 707 |
| 34h:05m | 708 |
| 34h:55m | 706 |
| 35h:40m | 708 |
| 36h:25m | 707 |
| 37h:10m | 708 |
| 38h:00m | 708 |
| 38h:45m | 710 |
| 39h:30m | 710 |
| 40h:15m | 710 |
| 41h:05m | 707 |
| 41h:50m | 708 |
| 42h:35m | 708 |
| 43h:20m | 707 |
| 44h:10m | 711 |
| 44h:55m | 707 |
| 45h:40m | 707 |
| 46h:25m | 710 |
| 47h:15m | 709 |
| 48h:00m | 708 |
| 48h:05m | 729 |
| 48h:50m | 734 |
| 49h:40m | 729 |
| 50h:25m | 730 |
| 51h:10m | 730 |
| 51h:55m | 730 |
| 52h:45m | 730 |
| 53h:30m | 732 |
| 54h:15m | 730 |
| 55h:00m | 732 |
| 55h:50m | 732 |
| 56h:35m | 733 |
| 57h:20m | 733 |
| 58h:05m | 732 |
| 58h:55m | 734 |
| 59h:40m | 733 |
| 60h:25m | 732 |
| 61h:10m | 736 |
| 62h:00m | 733 |
| 62h:45m | 737 |
| 63h:30m | 737 |
| 64h:15m | 734 |
| 65h:05m | 736 |
| 65h:50m | 738 |
| 66h:35m | 737 |
| 67h:20m | 737 |
| 68h:10m | 735 |
| 68h:55m | 737 |
| 69h:40m | 737 |
| 70h:25m | 736 |
| 71h:15m | 738 |
| 72h:00m | 737 |

Pseudomonas Garamycin 1000MIC 5

| 00:05 | 406 |
| --- | --- |
| 02:25 | 412 |
| 04:45 | 419 |
| 07:05 | 426 |
| 09:20 | 430 |
| 11:40 | 433 |
| 14:00 | 437 |
| 16:20 | 437 |
| 18:40 | 436 |
| 21:00 | 440 |
| 23:15 | 443 |
| 25:35 | 446 |
| 27:55 | 447 |
| 30:15 | 453 |
| 32:35 | 456 |
| 34:55 | 459 |
| 37:10 | 459 |
| 39:30 | 463 |
| 41:50 | 465 |
| 44:10 | 467 |
| 46:30 | 468 |
| 48:50 | 472 |
| 51:05 | 474 |
| 53:25 | 473 |
| 55:45 | 478 |
| 58:05 | 480 |
| 60:25 | 481 |
| 62:45 | 484 |
| 65:00 | 484 |
| 67:20 | 489 |
| 69:40 | 486 |
| 72:00 | 489 |

Pseudomonas Garamycin 1000MIC 6

| 00:05 | 344 |
| --- | --- |
| 02:25 | 356 |
| 04:45 | 360 |
| 07:05 | 366 |
| 09:20 | 369 |
| 11:40 | 375 |
| 14:00 | 376 |
| 16:20 | 377 |
| 18:40 | 377 |
| 21:00 | 382 |
| 23:15 | 386 |
| 25:35 | 393 |
| 27:55 | 399 |
| 30:15 | 404 |
| 32:35 | 410 |
| 34:55 | 415 |
| 37:10 | 418 |
| 39:30 | 424 |
| 41:50 | 425 |
| 44:10 | 427 |
| 46:30 | 433 |
| 48:50 | 438 |
| 51:05 | 437 |
| 53:25 | 441 |
| 55:45 | 444 |
| 58:05 | 442 |
| 60:25 | 447 |
| 62:45 | 446 |
| 65:00 | 448 |
| 67:20 | 450 |
| 69:40 | 452 |
| 72:00 | 452 |

Pseudomonas Garamycin 1000MIC 7

| 00:05 | 444 |
| --- | --- |
| 02:25 | 446 |
| 04:45 | 449 |
| 07:05 | 449 |
| 09:20 | 451 |
| 11:40 | 456 |
| 14:00 | 458 |
| 16:20 | 464 |
| 18:40 | 468 |
| 21:00 | 471 |
| 23:15 | 477 |
| 25:35 | 476 |
| 27:55 | 479 |
| 30:15 | 486 |
| 32:35 | 486 |
| 34:55 | 488 |
| 37:10 | 491 |
| 39:30 | 494 |
| 41:50 | 493 |
| 44:10 | 498 |
| 46:30 | 498 |
| 48:50 | 497 |
| 51:05 | 500 |
| 53:25 | 503 |
| 55:45 | 500 |
| 58:05 | 504 |
| 60:25 | 505 |
| 62:45 | 504 |
| 65:00 | 502 |
| 67:20 | 505 |
| 69:40 | 504 |
| 72:00 | 502 |

Pseudomonas Garamycin 1000MIC 8

| 00:05 | 427 |
| --- | --- |
| 02:25 | 422 |
| 04:45 | 422 |
| 07:05 | 425 |
| 09:20 | 425 |
| 11:40 | 429 |
| 14:00 | 433 |
| 16:20 | 434 |
| 18:40 | 436 |
| 21:00 | 437 |
| 23:15 | 439 |
| 25:35 | 439 |
| 27:55 | 443 |
| 30:15 | 446 |
| 32:35 | 449 |
| 34:55 | 450 |
| 37:10 | 451 |
| 39:30 | 457 |
| 41:50 | 463 |
| 44:10 | 466 |
| 46:30 | 470 |
| 48:50 | 470 |
| 51:05 | 475 |
| 53:25 | 478 |
| 55:45 | 478 |
| 58:05 | 480 |
| 60:25 | 482 |
| 62:45 | 484 |
| 65:00 | 485 |
| 67:20 | 484 |
| 69:40 | 485 |
| 72:00 | 486 |

Pseudomonas Amikacin control 1

| 0h:05m | 520 |
| --- | --- |
| 0h:50m | 537 |
| 1h:40m | 643 |
| 2h:25m | 1138 |
| 3h:10m | 2732 |
| 3h:55m | 3662 |
| 4h:45m | 5058 |
| 5h:30m | 7575 |
| 6h:15m | 10615 |
| 7h:00m | 14859 |
| 7h:50m | 27810 |
| 8h:35m | 30538 |
| 9h:20m | 30897 |
| 10h:05m | 30898 |
| 10h:55m | 30901 |
| 11h:40m | 30902 |
| 12h:25m | 30902 |
| 13h:10m | 30903 |
| 14h:00m | 30903 |
| 14h:45m | 30903 |
| 15h:30m | 30903 |
| 16h:15m | 30904 |
| 17h:05m | 30905 |
| 17h:50m | 30904 |
| 18h:35m | 30903 |
| 19h:20m | 30905 |
| 20h:10m | 30904 |
| 20h:55m | 30906 |
| 21h:40m | 30905 |
| 22h:25m | 30908 |
| 23h:15m | 30903 |
| 24h:00m | 30977 |
| 24h:05m |  |
| 24h:50m |  |
| 25h:40m |  |
| 26h:25m |  |
| 27h:10m |  |
| 27h:55m |  |
| 28h:45m |  |
| 29h:30m |  |
| 30h:15m |  |
| 31h:00m |  |
| 31h:50m |  |
| 32h:35m |  |
| 33h:20m |  |
| 34h:05m |  |
| 34h:55m |  |
| 35h:40m |  |
| 36h:25m |  |
| 37h:10m |  |
| 38h:00m |  |
| 38h:45m |  |
| 39h:30m |  |
| 40h:15m |  |
| 41h:05m |  |
| 41h:50m |  |
| 42h:35m |  |
| 43h:20m |  |
| 44h:10m |  |
| 44h:55m |  |
| 45h:40m |  |
| 46h:25m |  |
| 47h:15m |  |
| 48h:00m |  |
| 48h:05m |  |
| 48h:50m |  |
| 49h:40m |  |
| 50h:25m |  |
| 51h:10m |  |
| 51h:55m |  |
| 52h:45m |  |
| 53h:30m |  |
| 54h:15m |  |
| 55h:00m |  |
| 55h:50m |  |
| 56h:35m |  |
| 57h:20m |  |
| 58h:05m |  |
| 58h:55m |  |
| 59h:40m |  |
| 60h:25m |  |
| 61h:10m |  |
| 62h:00m |  |
| 62h:45m |  |
| 63h:30m |  |
| 64h:15m |  |
| 65h:05m |  |
| 65h:50m |  |
| 66h:35m |  |
| 67h:20m |  |
| 68h:10m |  |
| 68h:55m |  |
| 69h:40m |  |
| 70h:25m |  |
| 71h:15m |  |
| 72h:00m |  |

Pseudomonas Amikacin control 2

| 0h:05m | 812 |
| --- | --- |
| 0h:50m | 791 |
| 1h:40m | 821 |
| 2h:25m | 901 |
| 3h:10m | 1141 |
| 3h:55m | 1439 |
| 4h:45m | 2125 |
| 5h:30m | 3427 |
| 6h:15m | 4364 |
| 7h:00m | 4970 |
| 7h:50m | 6041 |
| 8h:35m | 7608 |
| 9h:20m | 9577 |
| 10h:05m | 12009 |
| 10h:55m | 15955 |
| 11h:40m | 20231 |
| 12h:25m | 22142 |
| 13h:10m | 29491 |
| 14h:00m | 30982 |
| 14h:45m | 30968 |
| 15h:30m | 30963 |
| 16h:15m | 30958 |
| 17h:05m | 30958 |
| 17h:50m | 30955 |
| 18h:35m | 30952 |
| 19h:20m | 30954 |
| 20h:10m | 30955 |
| 20h:55m | 30954 |
| 21h:40m | 30951 |
| 22h:25m | 30952 |
| 23h:15m | 30953 |
| 24h:00m | 30954 |
| 24h:05m |  |
| 24h:50m |  |
| 25h:40m |  |
| 26h:25m |  |
| 27h:10m |  |
| 27h:55m |  |
| 28h:45m |  |
| 29h:30m |  |
| 30h:15m |  |
| 31h:00m |  |
| 31h:50m |  |
| 32h:35m |  |
| 33h:20m |  |
| 34h:05m |  |
| 34h:55m |  |
| 35h:40m |  |
| 36h:25m |  |
| 37h:10m |  |
| 38h:00m |  |
| 38h:45m |  |
| 39h:30m |  |
| 40h:15m |  |
| 41h:05m |  |
| 41h:50m |  |
| 42h:35m |  |
| 43h:20m |  |
| 44h:10m |  |
| 44h:55m |  |
| 45h:40m |  |
| 46h:25m |  |
| 47h:15m |  |
| 48h:00m |  |
| 48h:05m |  |
| 48h:50m |  |
| 49h:40m |  |
| 50h:25m |  |
| 51h:10m |  |
| 51h:55m |  |
| 52h:45m |  |
| 53h:30m |  |
| 54h:15m |  |
| 55h:00m |  |
| 55h:50m |  |
| 56h:35m |  |
| 57h:20m |  |
| 58h:05m |  |
| 58h:55m |  |
| 59h:40m |  |
| 60h:25m |  |
| 61h:10m |  |
| 62h:00m |  |
| 62h:45m |  |
| 63h:30m |  |
| 64h:15m |  |
| 65h:05m |  |
| 65h:50m |  |
| 66h:35m |  |
| 67h:20m |  |
| 68h:10m |  |
| 68h:55m |  |
| 69h:40m |  |
| 70h:25m |  |
| 71h:15m |  |
| 72h:00m |  |

Pseudomonas Amikacin control 3

| 0h:05m | 991 |
| --- | --- |
| 0h:50m | 1001 |
| 1h:40m | 1109 |
| 2h:25m | 1571 |
| 3h:10m | 3238 |
| 3h:55m | 4157 |
| 4h:45m | 5651 |
| 5h:30m | 8142 |
| 6h:15m | 11553 |
| 7h:00m | 13669 |
| 7h:50m | 24317 |
| 8h:35m | 30897 |
| 9h:20m | 30985 |
| 10h:05m | 30983 |
| 10h:55m | 30982 |
| 11h:40m | 30979 |
| 12h:25m | 30977 |
| 13h:10m | 30976 |
| 14h:00m | 30971 |
| 14h:45m | 30971 |
| 15h:30m | 30968 |
| 16h:15m | 30968 |
| 17h:05m | 30967 |
| 17h:50m | 30967 |
| 18h:35m | 30961 |
| 19h:20m | 30959 |
| 20h:10m | 30987 |
| 20h:55m | 30988 |
| 21h:40m | 30990 |
| 22h:25m | 30990 |
| 23h:15m | 30991 |
| 24h:00m | 30988 |
| 24h:05m |  |
| 24h:50m |  |
| 25h:40m |  |
| 26h:25m |  |
| 27h:10m |  |
| 27h:55m |  |
| 28h:45m |  |
| 29h:30m |  |
| 30h:15m |  |
| 31h:00m |  |
| 31h:50m |  |
| 32h:35m |  |
| 33h:20m |  |
| 34h:05m |  |
| 34h:55m |  |
| 35h:40m |  |
| 36h:25m |  |
| 37h:10m |  |
| 38h:00m |  |
| 38h:45m |  |
| 39h:30m |  |
| 40h:15m |  |
| 41h:05m |  |
| 41h:50m |  |
| 42h:35m |  |
| 43h:20m |  |
| 44h:10m |  |
| 44h:55m |  |
| 45h:40m |  |
| 46h:25m |  |
| 47h:15m |  |
| 48h:00m |  |
| 48h:05m |  |
| 48h:50m |  |
| 49h:40m |  |
| 50h:25m |  |
| 51h:10m |  |
| 51h:55m |  |
| 52h:45m |  |
| 53h:30m |  |
| 54h:15m |  |
| 55h:00m |  |
| 55h:50m |  |
| 56h:35m |  |
| 57h:20m |  |
| 58h:05m |  |
| 58h:55m |  |
| 59h:40m |  |
| 60h:25m |  |
| 61h:10m |  |
| 62h:00m |  |
| 62h:45m |  |
| 63h:30m |  |
| 64h:15m |  |
| 65h:05m |  |
| 65h:50m |  |
| 66h:35m |  |
| 67h:20m |  |
| 68h:10m |  |
| 68h:55m |  |
| 69h:40m |  |
| 70h:25m |  |
| 71h:15m |  |
| 72h:00m |  |

Pseudomonas Amikacin 1MIC 1

| 0h:05m | 736 |
| --- | --- |
| 0h:50m | 730 |
| 1h:40m | 734 |
| 2h:25m | 734 |
| 3h:10m | 732 |
| 3h:55m | 729 |
| 4h:45m | 729 |
| 5h:30m | 722 |
| 6h:15m | 730 |
| 7h:00m | 725 |
| 7h:50m | 725 |
| 8h:35m | 724 |
| 9h:20m | 722 |
| 10h:05m | 724 |
| 10h:55m | 728 |
| 11h:40m | 724 |
| 12h:25m | 722 |
| 13h:10m | 723 |
| 14h:00m | 725 |
| 14h:45m | 722 |
| 15h:30m | 725 |
| 16h:15m | 730 |
| 17h:05m | 733 |
| 17h:50m | 737 |
| 18h:35m | 742 |
| 19h:20m | 745 |
| 20h:10m | 748 |
| 20h:55m | 745 |
| 21h:40m | 752 |
| 22h:25m | 761 |
| 23h:15m | 762 |
| 24h:00m | 762 |
| 24h:05m | 633 |
| 24h:50m | 632 |
| 25h:40m | 635 |
| 26h:25m | 640 |
| 27h:10m | 635 |
| 27h:55m | 638 |
| 28h:45m | 638 |
| 29h:30m | 636 |
| 30h:15m | 637 |
| 31h:00m | 642 |
| 31h:50m | 645 |
| 32h:35m | 652 |
| 33h:20m | 656 |
| 34h:05m | 659 |
| 34h:55m | 672 |
| 35h:40m | 686 |
| 36h:25m | 701 |
| 37h:10m | 714 |
| 38h:00m | 743 |
| 38h:45m | 774 |
| 39h:30m | 816 |
| 40h:15m | 881 |
| 41h:05m | 963 |
| 41h:50m | 1137 |
| 42h:35m | 1260 |
| 43h:20m | 1465 |
| 44h:10m | 1851 |
| 44h:55m | 2424 |
| 45h:40m | 2640 |
| 46h:25m | 2874 |
| 47h:15m | 3048 |
| 48h:00m | 3291 |
| 48h:05m |  |
| 48h:50m |  |
| 49h:40m |  |
| 50h:25m |  |
| 51h:10m |  |
| 51h:55m |  |
| 52h:45m |  |
| 53h:30m |  |
| 54h:15m |  |
| 55h:00m |  |
| 55h:50m |  |
| 56h:35m |  |
| 57h:20m |  |
| 58h:05m |  |
| 58h:55m |  |
| 59h:40m |  |
| 60h:25m |  |
| 61h:10m |  |
| 62h:00m |  |
| 62h:45m |  |
| 63h:30m |  |
| 64h:15m |  |
| 65h:05m |  |
| 65h:50m |  |
| 66h:35m |  |
| 67h:20m |  |
| 68h:10m |  |
| 68h:55m |  |
| 69h:40m |  |
| 70h:25m |  |
| 71h:15m |  |
| 72h:00m |  |

Pseudomonas Amikacin 1MIC 2

| 0h:05m | 716 |
| --- | --- |
| 0h:50m | 717 |
| 1h:40m | 719 |
| 2h:25m | 720 |
| 3h:10m | 717 |
| 3h:55m | 719 |
| 4h:45m | 717 |
| 5h:30m | 717 |
| 6h:15m | 711 |
| 7h:00m | 712 |
| 7h:50m | 711 |
| 8h:35m | 709 |
| 9h:20m | 709 |
| 10h:05m | 711 |
| 10h:55m | 715 |
| 11h:40m | 717 |
| 12h:25m | 721 |
| 13h:10m | 726 |
| 14h:00m | 728 |
| 14h:45m | 731 |
| 15h:30m | 734 |
| 16h:15m | 736 |
| 17h:05m | 738 |
| 17h:50m | 739 |
| 18h:35m | 741 |
| 19h:20m | 744 |
| 20h:10m | 748 |
| 20h:55m | 746 |
| 21h:40m | 744 |
| 22h:25m | 749 |
| 23h:15m | 751 |
| 24h:00m | 751 |
| 24h:05m | 771 |
| 24h:50m | 795 |
| 25h:40m | 827 |
| 26h:25m | 857 |
| 27h:10m | 879 |
| 27h:55m | 914 |
| 28h:45m | 973 |
| 29h:30m | 1069 |
| 30h:15m | 1240 |
| 31h:00m | 1672 |
| 31h:50m | 1874 |
| 32h:35m | 2059 |
| 33h:20m | 2286 |
| 34h:05m | 2499 |
| 34h:55m | 2774 |
| 35h:40m | 5016 |
| 36h:25m | 9616 |
| 37h:10m | 11733 |
| 38h:00m | 13017 |
| 38h:45m | 14341 |
| 39h:30m | 14921 |
| 40h:15m | 15339 |
| 41h:05m | 15498 |
| 41h:50m | 15533 |
| 42h:35m | 15715 |
| 43h:20m | 16064 |
| 44h:10m | 16169 |
| 44h:55m | 16332 |
| 45h:40m | 16151 |
| 46h:25m | 16281 |
| 47h:15m | 16160 |
| 48h:00m | 16319 |
| 48h:05m |  |
| 48h:50m |  |
| 49h:40m |  |
| 50h:25m |  |
| 51h:10m |  |
| 51h:55m |  |
| 52h:45m |  |
| 53h:30m |  |
| 54h:15m |  |
| 55h:00m |  |
| 55h:50m |  |
| 56h:35m |  |
| 57h:20m |  |
| 58h:05m |  |
| 58h:55m |  |
| 59h:40m |  |
| 60h:25m |  |
| 61h:10m |  |
| 62h:00m |  |
| 62h:45m |  |
| 63h:30m |  |
| 64h:15m |  |
| 65h:05m |  |
| 65h:50m |  |
| 66h:35m |  |
| 67h:20m |  |
| 68h:10m |  |
| 68h:55m |  |
| 69h:40m |  |
| 70h:25m |  |
| 71h:15m |  |
| 72h:00m |  |

Pseudomonas Amikacin 1MIC 3

| 0h:05m | 712 |
| --- | --- |
| 0h:50m | 703 |
| 1h:40m | 732 |
| 2h:25m | 784 |
| 3h:10m | 843 |
| 3h:55m | 901 |
| 4h:45m | 933 |
| 5h:30m | 942 |
| 6h:15m | 941 |
| 7h:00m | 933 |
| 7h:50m | 925 |
| 8h:35m | 916 |
| 9h:20m | 909 |
| 10h:05m | 913 |
| 10h:55m | 918 |
| 11h:40m | 928 |
| 12h:25m | 948 |
| 13h:10m | 974 |
| 14h:00m | 1011 |
| 14h:45m | 1062 |
| 15h:30m | 1099 |
| 16h:15m | 1138 |
| 17h:05m | 1203 |
| 17h:50m | 1247 |
| 18h:35m | 1307 |
| 19h:20m | 1369 |
| 20h:10m | 1430 |
| 20h:55m | 1497 |
| 21h:40m | 1568 |
| 22h:25m | 1659 |
| 23h:15m | 1793 |
| 24h:00m | 1977 |
| 24h:05m |  |
| 24h:50m |  |
| 25h:40m |  |
| 26h:25m |  |
| 27h:10m |  |
| 27h:55m |  |
| 28h:45m |  |
| 29h:30m |  |
| 30h:15m |  |
| 31h:00m |  |
| 31h:50m |  |
| 32h:35m |  |
| 33h:20m |  |
| 34h:05m |  |
| 34h:55m |  |
| 35h:40m |  |
| 36h:25m |  |
| 37h:10m |  |
| 38h:00m |  |
| 38h:45m |  |
| 39h:30m |  |
| 40h:15m |  |
| 41h:05m |  |
| 41h:50m |  |
| 42h:35m |  |
| 43h:20m |  |
| 44h:10m |  |
| 44h:55m |  |
| 45h:40m |  |
| 46h:25m |  |
| 47h:15m |  |
| 48h:00m |  |
| 48h:05m |  |
| 48h:50m |  |
| 49h:40m |  |
| 50h:25m |  |
| 51h:10m |  |
| 51h:55m |  |
| 52h:45m |  |
| 53h:30m |  |
| 54h:15m |  |
| 55h:00m |  |
| 55h:50m |  |
| 56h:35m |  |
| 57h:20m |  |
| 58h:05m |  |
| 58h:55m |  |
| 59h:40m |  |
| 60h:25m |  |
| 61h:10m |  |
| 62h:00m |  |
| 62h:45m |  |
| 63h:30m |  |
| 64h:15m |  |
| 65h:05m |  |
| 65h:50m |  |
| 66h:35m |  |
| 67h:20m |  |
| 68h:10m |  |
| 68h:55m |  |
| 69h:40m |  |
| 70h:25m |  |
| 71h:15m |  |
| 72h:00m |  |

Pseudomonas Amikacin 1MIC 4

| 0h:05m | 614 |
| --- | --- |
| 0h:50m | 623 |
| 1h:40m | 626 |
| 2h:25m | 627 |
| 3h:10m | 626 |
| 3h:55m | 623 |
| 4h:45m | 623 |
| 5h:30m | 621 |
| 6h:15m | 620 |
| 7h:00m | 622 |
| 7h:50m | 619 |
| 8h:35m | 619 |
| 9h:20m | 621 |
| 10h:05m | 620 |
| 10h:55m | 620 |
| 11h:40m | 620 |
| 12h:25m | 625 |
| 13h:10m | 629 |
| 14h:00m | 638 |
| 14h:45m | 652 |
| 15h:30m | 663 |
| 16h:15m | 679 |
| 17h:05m | 696 |
| 17h:50m | 717 |
| 18h:35m | 742 |
| 19h:20m | 775 |
| 20h:10m | 826 |
| 20h:55m | 900 |
| 21h:40m | 1015 |
| 22h:25m | 1179 |
| 23h:15m | 1455 |
| 24h:00m | 1922 |
| 24h:05m |  |
| 24h:50m |  |
| 25h:40m |  |
| 26h:25m |  |
| 27h:10m |  |
| 27h:55m |  |
| 28h:45m |  |
| 29h:30m |  |
| 30h:15m |  |
| 31h:00m |  |
| 31h:50m |  |
| 32h:35m |  |
| 33h:20m |  |
| 34h:05m |  |
| 34h:55m |  |
| 35h:40m |  |
| 36h:25m |  |
| 37h:10m |  |
| 38h:00m |  |
| 38h:45m |  |
| 39h:30m |  |
| 40h:15m |  |
| 41h:05m |  |
| 41h:50m |  |
| 42h:35m |  |
| 43h:20m |  |
| 44h:10m |  |
| 44h:55m |  |
| 45h:40m |  |
| 46h:25m |  |
| 47h:15m |  |
| 48h:00m |  |
| 48h:05m |  |
| 48h:50m |  |
| 49h:40m |  |
| 50h:25m |  |
| 51h:10m |  |
| 51h:55m |  |
| 52h:45m |  |
| 53h:30m |  |
| 54h:15m |  |
| 55h:00m |  |
| 55h:50m |  |
| 56h:35m |  |
| 57h:20m |  |
| 58h:05m |  |
| 58h:55m |  |
| 59h:40m |  |
| 60h:25m |  |
| 61h:10m |  |
| 62h:00m |  |
| 62h:45m |  |
| 63h:30m |  |
| 64h:15m |  |
| 65h:05m |  |
| 65h:50m |  |
| 66h:35m |  |
| 67h:20m |  |
| 68h:10m |  |
| 68h:55m |  |
| 69h:40m |  |
| 70h:25m |  |
| 71h:15m |  |
| 72h:00m |  |

Pseudomonas Amikacin 1MIC 5

| 0h:05m | 825 |
| --- | --- |
| 0h:50m | 819 |
| 1h:40m | 816 |
| 2h:25m | 818 |
| 3h:10m | 816 |
| 3h:55m | 815 |
| 4h:45m | 812 |
| 5h:30m | 808 |
| 6h:15m | 805 |
| 7h:00m | 802 |
| 7h:50m | 803 |
| 8h:35m | 798 |
| 9h:20m | 797 |
| 10h:05m | 796 |
| 10h:55m | 796 |
| 11h:40m | 796 |
| 12h:25m | 797 |
| 13h:10m | 794 |
| 14h:00m | 796 |
| 14h:45m | 799 |
| 15h:30m | 798 |
| 16h:15m | 800 |
| 17h:05m | 801 |
| 17h:50m | 803 |
| 18h:35m | 801 |
| 19h:20m | 801 |
| 20h:10m | 803 |
| 20h:55m | 800 |
| 21h:40m | 800 |
| 22h:25m | 800 |
| 23h:15m | 802 |
| 24h:00m | 811 |
| 24h:05m | 774 |
| 24h:50m | 781 |
| 25h:40m | 783 |
| 26h:25m | 781 |
| 27h:10m | 787 |
| 27h:55m | 785 |
| 28h:45m | 786 |
| 29h:30m | 788 |
| 30h:15m | 787 |
| 31h:00m | 788 |
| 31h:50m | 790 |
| 32h:35m | 790 |
| 33h:20m | 791 |
| 34h:05m | 796 |
| 34h:55m | 793 |
| 35h:40m | 797 |
| 36h:25m | 799 |
| 37h:10m | 799 |
| 38h:00m | 802 |
| 38h:45m | 803 |
| 39h:30m | 805 |
| 40h:15m | 806 |
| 41h:05m | 805 |
| 41h:50m | 809 |
| 42h:35m | 812 |
| 43h:20m | 815 |
| 44h:10m | 821 |
| 44h:55m | 831 |
| 45h:40m | 838 |
| 46h:25m | 847 |
| 47h:15m | 858 |
| 48h:00m | 873 |
| 48h:05m | 898 |
| 48h:50m | 907 |
| 49h:40m | 923 |
| 50h:25m | 934 |
| 51h:10m | 952 |
| 51h:55m | 974 |
| 52h:45m | 989 |
| 53h:30m | 1017 |
| 54h:15m | 1032 |
| 55h:00m | 1055 |
| 55h:50m | 1071 |
| 56h:35m | 1094 |
| 57h:20m | 1117 |
| 58h:05m | 1134 |
| 58h:55m | 1162 |
| 59h:40m | 1200 |
| 60h:25m | 1230 |
| 61h:10m | 1273 |
| 62h:00m | 1322 |
| 62h:45m | 1357 |
| 63h:30m | 1406 |
| 64h:15m | 1467 |
| 65h:05m | 1537 |
| 65h:50m | 1606 |
| 66h:35m | 1733 |
| 67h:20m | 1819 |
| 68h:10m | 1900 |
| 68h:55m | 1988 |
| 69h:40m | 2045 |
| 70h:25m | 2105 |
| 71h:15m | 2160 |
| 72h:00m | 2234 |

Pseudomonas Amikacin 4MIC 1

| 0h:05m | 693 |
| --- | --- |
| 0h:50m | 699 |
| 1h:40m | 699 |
| 2h:25m | 702 |
| 3h:10m | 695 |
| 3h:55m | 701 |
| 4h:45m | 698 |
| 5h:30m | 696 |
| 6h:15m | 699 |
| 7h:00m | 696 |
| 7h:50m | 698 |
| 8h:35m | 695 |
| 9h:20m | 697 |
| 10h:05m | 697 |
| 10h:55m | 693 |
| 11h:40m | 694 |
| 12h:25m | 697 |
| 13h:10m | 697 |
| 14h:00m | 697 |
| 14h:45m | 695 |
| 15h:30m | 696 |
| 16h:15m | 696 |
| 17h:05m | 697 |
| 17h:50m | 696 |
| 18h:35m | 696 |
| 19h:20m | 695 |
| 20h:10m | 698 |
| 20h:55m | 697 |
| 21h:40m | 699 |
| 22h:25m | 697 |
| 23h:15m | 698 |
| 24h:00m | 701 |
| 24h:05m | 572 |
| 24h:50m | 571 |
| 25h:40m | 570 |
| 26h:25m | 571 |
| 27h:10m | 568 |
| 27h:55m | 570 |
| 28h:45m | 568 |
| 29h:30m | 569 |
| 30h:15m | 570 |
| 31h:00m | 571 |
| 31h:50m | 572 |
| 32h:35m | 574 |
| 33h:20m | 572 |
| 34h:05m | 570 |
| 34h:55m | 572 |
| 35h:40m | 572 |
| 36h:25m | 574 |
| 37h:10m | 570 |
| 38h:00m | 568 |
| 38h:45m | 567 |
| 39h:30m | 571 |
| 40h:15m | 570 |
| 41h:05m | 566 |
| 41h:50m | 570 |
| 42h:35m | 572 |
| 43h:20m | 570 |
| 44h:10m | 570 |
| 44h:55m | 571 |
| 45h:40m | 570 |
| 46h:25m | 572 |
| 47h:15m | 573 |
| 48h:00m | 571 |
| 48h:05m | 578 |
| 48h:50m | 580 |
| 49h:40m | 581 |
| 50h:25m | 579 |
| 51h:10m | 581 |
| 51h:55m | 579 |
| 52h:45m | 580 |
| 53h:30m | 580 |
| 54h:15m | 580 |
| 55h:00m | 579 |
| 55h:50m | 581 |
| 56h:35m | 580 |
| 57h:20m | 582 |
| 58h:05m | 581 |
| 58h:55m | 582 |
| 59h:40m | 580 |
| 60h:25m | 582 |
| 61h:10m | 582 |
| 62h:00m | 582 |
| 62h:45m | 582 |
| 63h:30m | 581 |
| 64h:15m | 580 |
| 65h:05m | 577 |
| 65h:50m | 581 |
| 66h:35m | 581 |
| 67h:20m | 581 |
| 68h:10m | 581 |
| 68h:55m | 580 |
| 69h:40m | 579 |
| 70h:25m | 581 |
| 71h:15m | 582 |
| 72h:00m | 576 |

Pseudomonas Amikacin 4MIC 2

| 0h:05m | 761 |
| --- | --- |
| 0h:50m | 759 |
| 1h:40m | 760 |
| 2h:25m | 767 |
| 3h:10m | 765 |
| 3h:55m | 763 |
| 4h:45m | 762 |
| 5h:30m | 759 |
| 6h:15m | 757 |
| 7h:00m | 759 |
| 7h:50m | 757 |
| 8h:35m | 756 |
| 9h:20m | 758 |
| 10h:05m | 758 |
| 10h:55m | 755 |
| 11h:40m | 755 |
| 12h:25m | 756 |
| 13h:10m | 758 |
| 14h:00m | 761 |
| 14h:45m | 765 |
| 15h:30m | 765 |
| 16h:15m | 766 |
| 17h:05m | 769 |
| 17h:50m | 771 |
| 18h:35m | 776 |
| 19h:20m | 779 |
| 20h:10m | 781 |
| 20h:55m | 779 |
| 21h:40m | 783 |
| 22h:25m | 783 |
| 23h:15m | 784 |
| 24h:00m | 787 |
| 24h:05m | 734 |
| 24h:50m | 731 |
| 25h:40m | 732 |
| 26h:25m | 732 |
| 27h:10m | 732 |
| 27h:55m | 733 |
| 28h:45m | 734 |
| 29h:30m | 735 |
| 30h:15m | 735 |
| 31h:00m | 734 |
| 31h:50m | 735 |
| 32h:35m | 734 |
| 33h:20m | 735 |
| 34h:05m | 735 |
| 34h:55m | 735 |
| 35h:40m | 735 |
| 36h:25m | 732 |
| 37h:10m | 735 |
| 38h:00m | 740 |
| 38h:45m | 740 |
| 39h:30m | 738 |
| 40h:15m | 738 |
| 41h:05m | 738 |
| 41h:50m | 734 |
| 42h:35m | 738 |
| 43h:20m | 740 |
| 44h:10m | 737 |
| 44h:55m | 738 |
| 45h:40m | 736 |
| 46h:25m | 736 |
| 47h:15m | 735 |
| 48h:00m | 738 |
| 48h:05m | 1020 |
| 48h:50m | 1022 |
| 49h:40m | 1024 |
| 50h:25m | 1020 |
| 51h:10m | 1015 |
| 51h:55m | 1018 |
| 52h:45m | 1016 |
| 53h:30m | 1019 |
| 54h:15m | 1014 |
| 55h:00m | 1015 |
| 55h:50m | 1013 |
| 56h:35m | 1013 |
| 57h:20m | 1012 |
| 58h:05m | 1019 |
| 58h:55m | 1016 |
| 59h:40m | 1017 |
| 60h:25m | 1015 |
| 61h:10m | 1015 |
| 62h:00m | 1014 |
| 62h:45m | 1016 |
| 63h:30m | 1016 |
| 64h:15m | 1014 |
| 65h:05m | 1017 |
| 65h:50m | 1016 |
| 66h:35m | 1019 |
| 67h:20m | 1022 |
| 68h:10m | 1023 |
| 68h:55m | 1029 |
| 69h:40m | 1032 |
| 70h:25m | 1040 |
| 71h:15m | 1048 |
| 72h:00m | 1065 |

Pseudomonas Amikacin 4MIC 3

| 0h:05m | 881 |
| --- | --- |
| 0h:50m | 881 |
| 1h:40m | 876 |
| 2h:25m | 875 |
| 3h:10m | 869 |
| 3h:55m | 868 |
| 4h:45m | 863 |
| 5h:30m | 861 |
| 6h:15m | 858 |
| 7h:00m | 861 |
| 7h:50m | 857 |
| 8h:35m | 858 |
| 9h:20m | 855 |
| 10h:05m | 857 |
| 10h:55m | 857 |
| 11h:40m | 859 |
| 12h:25m | 853 |
| 13h:10m | 856 |
| 14h:00m | 858 |
| 14h:45m | 861 |
| 15h:30m | 861 |
| 16h:15m | 865 |
| 17h:05m | 867 |
| 17h:50m | 870 |
| 18h:35m | 872 |
| 19h:20m | 872 |
| 20h:10m | 877 |
| 20h:55m | 877 |
| 21h:40m | 880 |
| 22h:25m | 879 |
| 23h:15m | 882 |
| 24h:00m | 879 |
| 24h:05m | 900 |
| 24h:50m | 906 |
| 25h:40m | 905 |
| 26h:25m | 905 |
| 27h:10m | 907 |
| 27h:55m | 905 |
| 28h:45m | 908 |
| 29h:30m | 907 |
| 30h:15m | 908 |
| 31h:00m | 910 |
| 31h:50m | 912 |
| 32h:35m | 915 |
| 33h:20m | 916 |
| 34h:05m | 917 |
| 34h:55m | 917 |
| 35h:40m | 918 |
| 36h:25m | 921 |
| 37h:10m | 922 |
| 38h:00m | 922 |
| 38h:45m | 920 |
| 39h:30m | 925 |
| 40h:15m | 926 |
| 41h:05m | 928 |
| 41h:50m | 926 |
| 42h:35m | 927 |
| 43h:20m | 933 |
| 44h:10m | 934 |
| 44h:55m | 936 |
| 45h:40m | 938 |
| 46h:25m | 940 |
| 47h:15m | 943 |
| 48h:00m | 939 |
| 48h:05m | 603 |
| 48h:50m | 604 |
| 49h:40m | 601 |
| 50h:25m | 603 |
| 51h:10m | 605 |
| 51h:55m | 606 |
| 52h:45m | 609 |
| 53h:30m | 610 |
| 54h:15m | 611 |
| 55h:00m | 611 |
| 55h:50m | 607 |
| 56h:35m | 605 |
| 57h:20m | 604 |
| 58h:05m | 605 |
| 58h:55m | 606 |
| 59h:40m | 606 |
| 60h:25m | 605 |
| 61h:10m | 604 |
| 62h:00m | 606 |
| 62h:45m | 606 |
| 63h:30m | 604 |
| 64h:15m | 605 |
| 65h:05m | 606 |
| 65h:50m | 608 |
| 66h:35m | 608 |
| 67h:20m | 605 |
| 68h:10m | 605 |
| 68h:55m | 604 |
| 69h:40m | 605 |
| 70h:25m | 606 |
| 71h:15m | 605 |
| 72h:00m | 604 |

Pseudomonas Amikacin 4MIC 4

| 0h:05m | 1272 |
| --- | --- |
| 0h:50m | 1254 |
| 1h:40m | 1252 |
| 2h:25m | 1259 |
| 3h:10m | 1255 |
| 3h:55m | 1254 |
| 4h:45m | 1253 |
| 5h:30m | 1245 |
| 6h:15m | 1245 |
| 7h:00m | 1244 |
| 7h:50m | 1243 |
| 8h:35m | 1241 |
| 9h:20m | 1240 |
| 10h:05m | 1238 |
| 10h:55m | 1239 |
| 11h:40m | 1239 |
| 12h:25m | 1240 |
| 13h:10m | 1240 |
| 14h:00m | 1241 |
| 14h:45m | 1242 |
| 15h:30m | 1241 |
| 16h:15m | 1243 |
| 17h:05m | 1241 |
| 17h:50m | 1237 |
| 18h:35m | 1240 |
| 19h:20m | 1237 |
| 20h:10m | 1236 |
| 20h:55m | 1235 |
| 21h:40m | 1234 |
| 22h:25m | 1238 |
| 23h:15m | 1234 |
| 24h:00m | 1236 |
| 24h:05m | 642 |
| 24h:50m | 649 |
| 25h:40m | 653 |
| 26h:25m | 654 |
| 27h:10m | 657 |
| 27h:55m | 654 |
| 28h:45m | 658 |
| 29h:30m | 657 |
| 30h:15m | 659 |
| 31h:00m | 658 |
| 31h:50m | 660 |
| 32h:35m | 661 |
| 33h:20m | 660 |
| 34h:05m | 664 |
| 34h:55m | 663 |
| 35h:40m | 666 |
| 36h:25m | 663 |
| 37h:10m | 666 |
| 38h:00m | 666 |
| 38h:45m | 666 |
| 39h:30m | 666 |
| 40h:15m | 665 |
| 41h:05m | 666 |
| 41h:50m | 668 |
| 42h:35m | 668 |
| 43h:20m | 670 |
| 44h:10m | 669 |
| 44h:55m | 674 |
| 45h:40m | 673 |
| 46h:25m | 671 |
| 47h:15m | 673 |
| 48h:00m | 672 |
| 48h:05m | 630 |
| 48h:50m | 630 |
| 49h:40m | 627 |
| 50h:25m | 629 |
| 51h:10m | 631 |
| 51h:55m | 631 |
| 52h:45m | 631 |
| 53h:30m | 633 |
| 54h:15m | 631 |
| 55h:00m | 632 |
| 55h:50m | 631 |
| 56h:35m | 631 |
| 57h:20m | 632 |
| 58h:05m | 630 |
| 58h:55m | 630 |
| 59h:40m | 630 |
| 60h:25m | 629 |
| 61h:10m | 629 |
| 62h:00m | 630 |
| 62h:45m | 629 |
| 63h:30m | 629 |
| 64h:15m | 629 |
| 65h:05m | 629 |
| 65h:50m | 629 |
| 66h:35m | 630 |
| 67h:20m | 632 |
| 68h:10m | 630 |
| 68h:55m | 629 |
| 69h:40m | 630 |
| 70h:25m | 632 |
| 71h:15m | 632 |
| 72h:00m | 630 |

Pseudomonas Amikacin 1000MIC 1

| 0h:05m | 651 |
| --- | --- |
| 0h:50m | ----- |
| 1h:40m | 653 |
| 2h:25m | 662 |
| 3h:10m | 656 |
| 3h:55m | 657 |
| 4h:45m | 658 |
| 5h:30m | 664 |
| 6h:15m | 665 |
| 7h:00m | 668 |
| 7h:50m | 671 |
| 8h:35m | 671 |
| 9h:20m | 670 |
| 10h:05m | 671 |
| 10h:55m | 672 |
| 11h:40m | 670 |
| 12h:25m | 667 |
| 13h:10m | 669 |
| 14h:00m | 670 |
| 14h:45m | 670 |
| 15h:30m | 670 |
| 16h:15m | 674 |
| 17h:05m | 669 |
| 17h:50m | 674 |
| 18h:35m | 674 |
| 19h:20m | 676 |
| 20h:10m | 679 |
| 20h:55m | 685 |
| 21h:40m | 683 |
| 22h:25m | 689 |
| 23h:15m | 689 |
| 24h:00m | 693 |
| 24h:05m | 677 |
| 24h:50m | 684 |
| 25h:40m | 691 |
| 26h:25m | 699 |
| 27h:10m | 703 |
| 27h:55m | 709 |
| 28h:45m | 716 |
| 29h:30m | 720 |
| 30h:15m | 720 |
| 31h:00m | 724 |
| 31h:50m | 728 |
| 32h:35m | 729 |
| 33h:20m | 731 |
| 34h:05m | 733 |
| 34h:55m | 739 |
| 35h:40m | 739 |
| 36h:25m | 739 |
| 37h:10m | 742 |
| 38h:00m | 744 |
| 38h:45m | 747 |
| 39h:30m | 746 |
| 40h:15m | 750 |
| 41h:05m | 748 |
| 41h:50m | 747 |
| 42h:35m | 753 |
| 43h:20m | 752 |
| 44h:10m | 755 |
| 44h:55m | 752 |
| 45h:40m | 754 |
| 46h:25m | 754 |
| 47h:15m | 757 |
| 48h:00m | 759 |
| 48h:05m | 845 |
| 48h:50m | 852 |
| 49h:40m | 847 |
| 50h:25m | 848 |
| 51h:10m | 850 |
| 51h:55m | 845 |
| 52h:45m | 850 |
| 53h:30m | 846 |
| 54h:15m | 847 |
| 55h:00m | 847 |
| 55h:50m | 845 |
| 56h:35m | 847 |
| 57h:20m | 848 |
| 58h:05m | 846 |
| 58h:55m | 848 |
| 59h:40m | 849 |
| 60h:25m | 847 |
| 61h:10m | 845 |
| 62h:00m | 846 |
| 62h:45m | 846 |
| 63h:30m | 848 |
| 64h:15m | 843 |
| 65h:05m | 840 |
| 65h:50m | 838 |
| 66h:35m | 845 |
| 67h:20m | 844 |
| 68h:10m | 843 |
| 68h:55m | 845 |
| 69h:40m | 846 |
| 70h:25m | 843 |
| 71h:15m | 838 |
| 72h:00m | 834 |

Pseudomonas Amikacin 1000MIC 2

| 0h:05m | 871 |
| --- | --- |
| 0h:50m | 871 |
| 1h:40m | 875 |
| 2h:25m | 881 |
| 3h:10m | 885 |
| 3h:55m | 887 |
| 4h:45m | 899 |
| 5h:30m | 897 |
| 6h:15m | 899 |
| 7h:00m | 898 |
| 7h:50m | 898 |
| 8h:35m | 895 |
| 9h:20m | 900 |
| 10h:05m | 899 |
| 10h:55m | 896 |
| 11h:40m | 898 |
| 12h:25m | 897 |
| 13h:10m | 897 |
| 14h:00m | 895 |
| 14h:45m | 898 |
| 15h:30m | 898 |
| 16h:15m | 896 |
| 17h:05m | 897 |
| 17h:50m | 895 |
| 18h:35m | 896 |
| 19h:20m | 897 |
| 20h:10m | 897 |
| 20h:55m | 892 |
| 21h:40m | 896 |
| 22h:25m | 894 |
| 23h:15m | 894 |
| 24h:00m | 893 |
| 24h:05m | 698 |
| 24h:50m | 699 |
| 25h:40m | 700 |
| 26h:25m | 703 |
| 27h:10m | 702 |
| 27h:55m | 700 |
| 28h:45m | 701 |
| 29h:30m | 701 |
| 30h:15m | 701 |
| 31h:00m | 701 |
| 31h:50m | 705 |
| 32h:35m | 703 |
| 33h:20m | 703 |
| 34h:05m | 703 |
| 34h:55m | 704 |
| 35h:40m | 701 |
| 36h:25m | 701 |
| 37h:10m | 703 |
| 38h:00m | 703 |
| 38h:45m | 703 |
| 39h:30m | 703 |
| 40h:15m | 703 |
| 41h:05m | 704 |
| 41h:50m | 701 |
| 42h:35m | 702 |
| 43h:20m | 703 |
| 44h:10m | 701 |
| 44h:55m | 702 |
| 45h:40m | 702 |
| 46h:25m | 703 |
| 47h:15m | 703 |
| 48h:00m | 703 |
| 48h:05m | 693 |
| 48h:50m | 691 |
| 49h:40m | 689 |
| 50h:25m | 690 |
| 51h:10m | 690 |
| 51h:55m | 691 |
| 52h:45m | 689 |
| 53h:30m | 689 |
| 54h:15m | 692 |
| 55h:00m | 692 |
| 55h:50m | 691 |
| 56h:35m | 690 |
| 57h:20m | 692 |
| 58h:05m | 692 |
| 58h:55m | 694 |
| 59h:40m | 695 |
| 60h:25m | 694 |
| 61h:10m | 694 |
| 62h:00m | 690 |
| 62h:45m | 693 |
| 63h:30m | 695 |
| 64h:15m | 693 |
| 65h:05m | 694 |
| 65h:50m | 692 |
| 66h:35m | 691 |
| 67h:20m | 693 |
| 68h:10m | 696 |
| 68h:55m | 693 |
| 69h:40m | 693 |
| 70h:25m | 692 |
| 71h:15m | 694 |
| 72h:00m | 693 |

Pseudomonas Amikacin 1000MIC 3

| 0h:05m | 692 |
| --- | --- |
| 0h:50m | 664 |
| 1h:40m | 659 |
| 2h:25m | 661 |
| 3h:10m | 660 |
| 3h:55m | 658 |
| 4h:45m | 661 |
| 5h:30m | 659 |
| 6h:15m | 656 |
| 7h:00m | 654 |
| 7h:50m | 654 |
| 8h:35m | 652 |
| 9h:20m | 652 |
| 10h:05m | 650 |
| 10h:55m | 650 |
| 11h:40m | 651 |
| 12h:25m | 651 |
| 13h:10m | 649 |
| 14h:00m | 651 |
| 14h:45m | 651 |
| 15h:30m | 651 |
| 16h:15m | 651 |
| 17h:05m | 653 |
| 17h:50m | 651 |
| 18h:35m | 652 |
| 19h:20m | 652 |
| 20h:10m | 651 |
| 20h:55m | 649 |
| 21h:40m | 647 |
| 22h:25m | 647 |
| 23h:15m | 647 |
| 24h:00m | 648 |
| 24h:05m |  |
| 24h:50m |  |
| 25h:40m |  |
| 26h:25m |  |
| 27h:10m |  |
| 27h:55m |  |
| 28h:45m |  |
| 29h:30m |  |
| 30h:15m |  |
| 31h:00m |  |
| 31h:50m |  |
| 32h:35m |  |
| 33h:20m |  |
| 34h:05m |  |
| 34h:55m |  |
| 35h:40m |  |
| 36h:25m |  |
| 37h:10m |  |
| 38h:00m |  |
| 38h:45m |  |
| 39h:30m |  |
| 40h:15m |  |
| 41h:05m |  |
| 41h:50m |  |
| 42h:35m |  |
| 43h:20m |  |
| 44h:10m |  |
| 44h:55m |  |
| 45h:40m |  |
| 46h:25m |  |
| 47h:15m |  |
| 48h:00m |  |
| 48h:05m | 768 |
| 48h:50m | 768 |
| 49h:40m | 769 |
| 50h:25m | 770 |
| 51h:10m | 770 |
| 51h:55m | 772 |
| 52h:45m | 771 |
| 53h:30m | 771 |
| 54h:15m | 771 |
| 55h:00m | 771 |
| 55h:50m | 770 |
| 56h:35m | 770 |
| 57h:20m | 769 |
| 58h:05m | 768 |
| 58h:55m | 768 |
| 59h:40m | 767 |
| 60h:25m | 766 |
| 61h:10m | 764 |
| 62h:00m | 766 |
| 62h:45m | 764 |
| 63h:30m | 765 |
| 64h:15m | 763 |
| 65h:05m | 763 |
| 65h:50m | 764 |
| 66h:35m | 762 |
| 67h:20m | 762 |
| 68h:10m | 763 |
| 68h:55m | 763 |
| 69h:40m | 763 |
| 70h:25m | 764 |
| 71h:15m | 765 |
| 72h:00m | 764 |

Pseudomonas Amikacin 1000MIC 4

| 0h:05m | 1079 |
| --- | --- |
| 0h:50m | 1069 |
| 1h:40m | 1071 |
| 2h:25m | 1084 |
| 3h:10m | 1085 |
| 3h:55m | 1079 |
| 4h:45m | 1086 |
| 5h:30m | 1088 |
| 6h:15m | 1082 |
| 7h:00m | 1080 |
| 7h:50m | 1079 |
| 8h:35m | 1076 |
| 9h:20m | 1077 |
| 10h:05m | 1074 |
| 10h:55m | 1075 |
| 11h:40m | 1075 |
| 12h:25m | 1073 |
| 13h:10m | 1074 |
| 14h:00m | 1076 |
| 14h:45m | 1075 |
| 15h:30m | 1077 |
| 16h:15m | 1076 |
| 17h:05m | 1074 |
| 17h:50m | 1074 |
| 18h:35m | 1072 |
| 19h:20m | 1075 |
| 20h:10m | 1077 |
| 20h:55m | 1084 |
| 21h:40m | 1092 |
| 22h:25m | 1117 |
| 23h:15m | 1143 |
| 24h:00m | 1163 |
| 24h:05m |  |
| 24h:50m |  |
| 25h:40m |  |
| 26h:25m |  |
| 27h:10m |  |
| 27h:55m |  |
| 28h:45m |  |
| 29h:30m |  |
| 30h:15m |  |
| 31h:00m |  |
| 31h:50m |  |
| 32h:35m |  |
| 33h:20m |  |
| 34h:05m |  |
| 34h:55m |  |
| 35h:40m |  |
| 36h:25m |  |
| 37h:10m |  |
| 38h:00m |  |
| 38h:45m |  |
| 39h:30m |  |
| 40h:15m |  |
| 41h:05m |  |
| 41h:50m |  |
| 42h:35m |  |
| 43h:20m |  |
| 44h:10m |  |
| 44h:55m |  |
| 45h:40m |  |
| 46h:25m |  |
| 47h:15m |  |
| 48h:00m |  |
| 48h:05m | 766 |
| 48h:50m | 768 |
| 49h:40m | 768 |
| 50h:25m | 768 |
| 51h:10m | 765 |
| 51h:55m | 764 |
| 52h:45m | 764 |
| 53h:30m | 768 |
| 54h:15m | 763 |
| 55h:00m | 767 |
| 55h:50m | 767 |
| 56h:35m | 766 |
| 57h:20m | 768 |
| 58h:05m | 769 |
| 58h:55m | 769 |
| 59h:40m | 767 |
| 60h:25m | 768 |
| 61h:10m | 768 |
| 62h:00m | 768 |
| 62h:45m | 766 |
| 63h:30m | 771 |
| 64h:15m | 768 |
| 65h:05m | 770 |
| 65h:50m | 771 |
| 66h:35m | 775 |
| 67h:20m | 775 |
| 68h:10m | 778 |
| 68h:55m | 776 |
| 69h:40m | 779 |
| 70h:25m | 782 |
| 71h:15m | 784 |
| 72h:00m | 788 |

Pseudomonas Amikacin 1000MIC 5

| 0h:05m | 519 |
| --- | --- |
| 0h:50m | 510 |
| 1h:40m | 508 |
| 2h:25m | 511 |
| 3h:10m | 510 |
| 3h:55m | 507 |
| 4h:45m | 509 |
| 5h:30m | 510 |
| 6h:15m | 512 |
| 7h:00m | 511 |
| 7h:50m | 515 |
| 8h:35m | 514 |
| 9h:20m | 514 |
| 10h:05m | 515 |
| 10h:55m | 514 |
| 11h:40m | 514 |
| 12h:25m | 516 |
| 13h:10m | 516 |
| 14h:00m | 515 |
| 14h:45m | 515 |
| 15h:30m | 516 |
| 16h:15m | 517 |
| 17h:05m | 518 |
| 17h:50m | 519 |
| 18h:35m | 518 |
| 19h:20m | 518 |
| 20h:10m | 522 |
| 20h:55m | 521 |
| 21h:40m | 518 |
| 22h:25m | 518 |
| 23h:15m | 519 |
| 24h:00m | 521 |
| 24h:05m | 568 |
| 24h:50m | 569 |
| 25h:40m | 568 |
| 26h:25m | 570 |
| 27h:10m | 572 |
| 27h:55m | 574 |
| 28h:45m | 573 |
| 29h:30m | 574 |
| 30h:15m | 574 |
| 31h:00m | 576 |
| 31h:50m | 577 |
| 32h:35m | 576 |
| 33h:20m | 579 |
| 34h:05m | 577 |
| 34h:55m | 579 |
| 35h:40m | 578 |
| 36h:25m | 577 |
| 37h:10m | 578 |
| 38h:00m | 580 |
| 38h:45m | 577 |
| 39h:30m | 577 |
| 40h:15m | 576 |
| 41h:05m | 577 |
| 41h:50m | 579 |
| 42h:35m | 578 |
| 43h:20m | 584 |
| 44h:10m | 584 |
| 44h:55m | 585 |
| 45h:40m | 586 |
| 46h:25m | 586 |
| 47h:15m | 586 |
| 48h:00m | 586 |
| 48h:05m | 582 |
| 48h:50m | 582 |
| 49h:40m | 584 |
| 50h:25m | 582 |
| 51h:10m | 583 |
| 51h:55m | 581 |
| 52h:45m | 585 |
| 53h:30m | 584 |
| 54h:15m | 584 |
| 55h:00m | 584 |
| 55h:50m | 582 |
| 56h:35m | 584 |
| 57h:20m | 584 |
| 58h:05m | 584 |
| 58h:55m | 583 |
| 59h:40m | 583 |
| 60h:25m | 584 |
| 61h:10m | 584 |
| 62h:00m | 585 |
| 62h:45m | 585 |
| 63h:30m | 584 |
| 64h:15m | 585 |
| 65h:05m | 581 |
| 65h:50m | 582 |
| 66h:35m | 584 |
| 67h:20m | 584 |
| 68h:10m | 583 |
| 68h:55m | 585 |
| 69h:40m | 584 |
| 70h:25m | 582 |
| 71h:15m | 583 |
| 72h:00m | 586 |

Pseudomonas Amikacin 1000MIC 6

| 0h:05m | 624 |
| --- | --- |
| 0h:50m | 617 |
| 1h:40m | 614 |
| 2h:25m | 614 |
| 3h:10m | 614 |
| 3h:55m | 612 |
| 4h:45m | 615 |
| 5h:30m | 614 |
| 6h:15m | 616 |
| 7h:00m | 613 |
| 7h:50m | 615 |
| 8h:35m | 616 |
| 9h:20m | 616 |
| 10h:05m | 616 |
| 10h:55m | 617 |
| 11h:40m | 615 |
| 12h:25m | 616 |
| 13h:10m | 616 |
| 14h:00m | 613 |
| 14h:45m | 615 |
| 15h:30m | 614 |
| 16h:15m | 614 |
| 17h:05m | 614 |
| 17h:50m | 614 |
| 18h:35m | 615 |
| 19h:20m | 615 |
| 20h:10m | 614 |
| 20h:55m | 613 |
| 21h:40m | 614 |
| 22h:25m | 611 |
| 23h:15m | 614 |
| 24h:00m | 612 |
| 24h:05m | 591 |
| 24h:50m | 594 |
| 25h:40m | 596 |
| 26h:25m | 597 |
| 27h:10m | 596 |
| 27h:55m | 597 |
| 28h:45m | 596 |
| 29h:30m | 598 |
| 30h:15m | 599 |
| 31h:00m | 598 |
| 31h:50m | 600 |
| 32h:35m | 601 |
| 33h:20m | 603 |
| 34h:05m | 603 |
| 34h:55m | 602 |
| 35h:40m | 602 |
| 36h:25m | 604 |
| 37h:10m | 604 |
| 38h:00m | 605 |
| 38h:45m | 604 |
| 39h:30m | 606 |
| 40h:15m | 606 |
| 41h:05m | 605 |
| 41h:50m | 607 |
| 42h:35m | 608 |
| 43h:20m | 607 |
| 44h:10m | 609 |
| 44h:55m | 610 |
| 45h:40m | 610 |
| 46h:25m | 609 |
| 47h:15m | 608 |
| 48h:00m | 609 |
| 48h:05m | 607 |
| 48h:50m | 606 |
| 49h:40m | 605 |
| 50h:25m | 606 |
| 51h:10m | 607 |
| 51h:55m | 607 |
| 52h:45m | 607 |
| 53h:30m | 609 |
| 54h:15m | 607 |
| 55h:00m | 607 |
| 55h:50m | 606 |
| 56h:35m | 605 |
| 57h:20m | 605 |
| 58h:05m | 606 |
| 58h:55m | 605 |
| 59h:40m | 604 |
| 60h:25m | 602 |
| 61h:10m | 602 |
| 62h:00m | 602 |
| 62h:45m | 603 |
| 63h:30m | 603 |
| 64h:15m | 603 |
| 65h:05m | 603 |
| 65h:50m | 601 |
| 66h:35m | 602 |
| 67h:20m | 604 |
| 68h:10m | 604 |
| 68h:55m | 605 |
| 69h:40m | 604 |
| 70h:25m | 603 |
| 71h:15m | 603 |
| 72h:00m | 604 |

E.Coli Garamycin control 1

| 0h:05m | 1128 |
| --- | --- |
| 0h:50m | 1156 |
| 1h:40m | 1344 |
| 2h:25m | 2884 |
| 3h:10m | 17318 |
| 3h:55m | 30964 |
| 4h:45m | 30934 |
| 5h:30m | 30927 |
| 6h:15m | 30926 |
| 7h:00m | 30924 |
| 7h:50m | 30925 |
| 8h:35m | 30924 |
| 9h:20m | 30925 |
| 10h:05m | 30926 |
| 10h:55m | 30926 |
| 11h:40m | 30925 |
| 12h:25m | 30926 |
| 13h:10m | 30926 |
| 14h:00m | 30926 |
| 14h:45m | 30926 |
| 15h:30m | 30926 |
| 16h:15m | 30927 |
| 17h:05m | 30926 |
| 17h:50m | 30927 |
| 18h:35m | 30926 |
| 19h:20m | 30927 |
| 20h:10m | 30929 |
| 20h:55m | 30927 |
| 21h:40m | 30927 |
| 22h:25m | 30926 |
| 23h:15m | 30925 |
| 24h:00m | 30925 |
| 24h:05m |  |
| 24h:50m |  |
| 25h:40m |  |
| 26h:25m |  |
| 27h:10m |  |
| 27h:55m |  |
| 28h:45m |  |
| 29h:30m |  |
| 30h:15m |  |
| 31h:00m |  |
| 31h:50m |  |
| 32h:35m |  |
| 33h:20m |  |
| 34h:05m |  |
| 34h:55m |  |
| 35h:40m |  |
| 36h:25m |  |
| 37h:10m |  |
| 38h:00m |  |
| 38h:45m |  |
| 39h:30m |  |
| 40h:15m |  |
| 41h:05m |  |
| 41h:50m |  |
| 42h:35m |  |
| 43h:20m |  |
| 44h:10m |  |
| 44h:55m |  |
| 45h:40m |  |
| 46h:25m |  |
| 47h:15m |  |
| 48h:00m |  |
| 48h:05m |  |
| 48h:50m |  |
| 49h:40m |  |
| 50h:25m |  |
| 51h:10m |  |
| 51h:55m |  |
| 52h:45m |  |
| 53h:30m |  |
| 54h:15m |  |
| 55h:00m |  |
| 55h:50m |  |
| 56h:35m |  |
| 57h:20m |  |
| 58h:05m |  |
| 58h:55m |  |
| 59h:40m |  |
| 60h:25m |  |
| 61h:10m |  |
| 62h:00m |  |
| 62h:45m |  |
| 63h:30m |  |
| 64h:15m |  |
| 65h:05m |  |
| 65h:50m |  |
| 66h:35m |  |
| 67h:20m |  |
| 68h:10m |  |
| 68h:55m |  |
| 69h:40m |  |
| 70h:25m |  |
| 71h:15m |  |
| 72h:00m |  |

E.Coli Garamycin control 2

| Time | EC Cont E.5 |
| --- | --- |
| 0h:05m | 609 |
| 0h:50m | 622 |
| 1h:40m | 800 |
| 2h:25m | 2209 |
| 3h:10m | 13611 |
| 3h:55m | 30968 |
| 4h:45m | 30933 |
| 5h:30m | 30925 |
| 6h:15m | 30925 |
| 7h:00m | 30927 |
| 7h:50m | 30927 |
| 8h:35m | 30927 |
| 9h:20m | 30926 |
| 10h:05m | 30925 |
| 10h:55m | 30925 |
| 11h:40m | 30925 |
| 12h:25m | 30925 |
| 13h:10m | 30926 |
| 14h:00m | 30925 |
| 14h:45m | 30925 |
| 15h:30m | 30925 |
| 16h:15m | 30926 |
| 17h:05m | 30925 |
| 17h:50m | 30925 |
| 18h:35m | 30925 |
| 19h:20m | 30925 |
| 20h:10m | 30927 |
| 20h:55m | 30926 |
| 21h:40m | 30926 |
| 22h:25m | 30926 |
| 23h:15m | 30926 |
| 24h:00m | 30925 |
| 24h:05m |  |
| 24h:50m |  |
| 25h:40m |  |
| 26h:25m |  |
| 27h:10m |  |
| 27h:55m |  |
| 28h:45m |  |
| 29h:30m |  |
| 30h:15m |  |
| 31h:00m |  |
| 31h:50m |  |
| 32h:35m |  |
| 33h:20m |  |
| 34h:05m |  |
| 34h:55m |  |
| 35h:40m |  |
| 36h:25m |  |
| 37h:10m |  |
| 38h:00m |  |
| 38h:45m |  |
| 39h:30m |  |
| 40h:15m |  |
| 41h:05m |  |
| 41h:50m |  |
| 42h:35m |  |
| 43h:20m |  |
| 44h:10m |  |
| 44h:55m |  |
| 45h:40m |  |
| 46h:25m |  |
| 47h:15m |  |
| 48h:00m |  |
| 48h:05m |  |
| 48h:50m |  |
| 49h:40m |  |
| 50h:25m |  |
| 51h:10m |  |
| 51h:55m |  |
| 52h:45m |  |
| 53h:30m |  |
| 54h:15m |  |
| 55h:00m |  |
| 55h:50m |  |
| 56h:35m |  |
| 57h:20m |  |
| 58h:05m |  |
| 58h:55m |  |
| 59h:40m |  |
| 60h:25m |  |
| 61h:10m |  |
| 62h:00m |  |
| 62h:45m |  |
| 63h:30m |  |
| 64h:15m |  |
| 65h:05m |  |
| 65h:50m |  |
| 66h:35m |  |
| 67h:20m |  |
| 68h:10m |  |
| 68h:55m |  |
| 69h:40m |  |
| 70h:25m |  |
| 71h:15m |  |
| 72h:00m |  |

E.Coli Garamycin control 3

| Time | EC Cont E.4 |
| --- | --- |
| 0h:05m | 860 |
| 0h:50m | 843 |
| 1h:40m | 960 |
| 2h:25m | 1686 |
| 3h:10m | 5806 |
| 3h:55m | 18986 |
| 4h:45m | 30967 |
| 5h:30m | 30936 |
| 6h:15m | 30929 |
| 7h:00m | 30928 |
| 7h:50m | 30928 |
| 8h:35m | 30928 |
| 9h:20m | 30928 |
| 10h:05m | 30929 |
| 10h:55m | 30929 |
| 11h:40m | 30929 |
| 12h:25m | 30928 |
| 13h:10m | 30930 |
| 14h:00m | 30930 |
| 14h:45m | 30930 |
| 15h:30m | 30930 |
| 16h:15m | 30930 |
| 17h:05m | 30930 |
| 17h:50m | 30930 |
| 18h:35m | 30931 |
| 19h:20m | 30929 |
| 20h:10m | 30928 |
| 20h:55m | 30926 |
| 21h:40m | 30926 |
| 22h:25m | 30927 |
| 23h:15m | 30927 |
| 24h:00m | 30926 |
| 24h:05m |  |
| 24h:50m |  |
| 25h:40m |  |
| 26h:25m |  |
| 27h:10m |  |
| 27h:55m |  |
| 28h:45m |  |
| 29h:30m |  |
| 30h:15m |  |
| 31h:00m |  |
| 31h:50m |  |
| 32h:35m |  |
| 33h:20m |  |
| 34h:05m |  |
| 34h:55m |  |
| 35h:40m |  |
| 36h:25m |  |
| 37h:10m |  |
| 38h:00m |  |
| 38h:45m |  |
| 39h:30m |  |
| 40h:15m |  |
| 41h:05m |  |
| 41h:50m |  |
| 42h:35m |  |
| 43h:20m |  |
| 44h:10m |  |
| 44h:55m |  |
| 45h:40m |  |
| 46h:25m |  |
| 47h:15m |  |
| 48h:00m |  |
| 48h:05m |  |
| 48h:50m |  |
| 49h:40m |  |
| 50h:25m |  |
| 51h:10m |  |
| 51h:55m |  |
| 52h:45m |  |
| 53h:30m |  |
| 54h:15m |  |
| 55h:00m |  |
| 55h:50m |  |
| 56h:35m |  |
| 57h:20m |  |
| 58h:05m |  |
| 58h:55m |  |
| 59h:40m |  |
| 60h:25m |  |
| 61h:10m |  |
| 62h:00m |  |
| 62h:45m |  |
| 63h:30m |  |
| 64h:15m |  |
| 65h:05m |  |
| 65h:50m |  |
| 66h:35m |  |
| 67h:20m |  |
| 68h:10m |  |
| 68h:55m |  |
| 69h:40m |  |
| 70h:25m |  |
| 71h:15m |  |
| 72h:00m |  |

E.Coli Garamycin control 4

| Time | EC Cont E.W |
| --- | --- |
| 0h:05m | 346 |
| 0h:50m | 344 |
| 1h:40m | 395 |
| 2h:25m | 702 |
| 3h:10m | 2681 |
| 3h:55m | 11244 |
| 4h:45m | 31022 |
| 5h:30m | 30992 |
| 6h:15m | 30983 |
| 7h:00m | 30980 |
| 7h:50m | 30978 |
| 8h:35m | 30978 |
| 9h:20m | 30978 |
| 10h:05m | 30978 |
| 10h:55m | 30979 |
| 11h:40m | 30979 |
| 12h:25m | 30979 |
| 13h:10m | 30979 |
| 14h:00m | 30979 |
| 14h:45m | 30980 |
| 15h:30m | 30982 |
| 16h:15m | 30980 |
| 17h:05m | 30982 |
| 17h:50m | 30980 |
| 18h:35m | 30980 |
| 19h:20m | 30979 |
| 20h:10m | 30980 |
| 20h:55m | 30979 |
| 21h:40m | 30979 |
| 22h:25m | 30979 |
| 23h:15m | 30979 |
| 24h:00m | 30982 |
| 24h:05m |  |
| 24h:50m |  |
| 25h:40m |  |
| 26h:25m |  |
| 27h:10m |  |
| 27h:55m |  |
| 28h:45m |  |
| 29h:30m |  |
| 30h:15m |  |
| 31h:00m |  |
| 31h:50m |  |
| 32h:35m |  |
| 33h:20m |  |
| 34h:05m |  |
| 34h:55m |  |
| 35h:40m |  |
| 36h:25m |  |
| 37h:10m |  |
| 38h:00m |  |
| 38h:45m |  |
| 39h:30m |  |
| 40h:15m |  |
| 41h:05m |  |
| 41h:50m |  |
| 42h:35m |  |
| 43h:20m |  |
| 44h:10m |  |
| 44h:55m |  |
| 45h:40m |  |
| 46h:25m |  |
| 47h:15m |  |
| 48h:00m |  |
| 48h:05m |  |
| 48h:50m |  |
| 49h:40m |  |
| 50h:25m |  |
| 51h:10m |  |
| 51h:55m |  |
| 52h:45m |  |
| 53h:30m |  |
| 54h:15m |  |
| 55h:00m |  |
| 55h:50m |  |
| 56h:35m |  |
| 57h:20m |  |
| 58h:05m |  |
| 58h:55m |  |
| 59h:40m |  |
| 60h:25m |  |
| 61h:10m |  |
| 62h:00m |  |
| 62h:45m |  |
| 63h:30m |  |
| 64h:15m |  |
| 65h:05m |  |
| 65h:50m |  |
| 66h:35m |  |
| 67h:20m |  |
| 68h:10m |  |
| 68h:55m |  |
| 69h:40m |  |
| 70h:25m |  |
| 71h:15m |  |
| 72h:00m |  |

E.Coli Garamycin 1MIC 1

| Time | EC 1MIC GARA E.3 |
| --- | --- |
| 0h:05m | 776 |
| 0h:50m | 793 |
| 1h:40m | 801 |
| 2h:25m | 802 |
| 3h:10m | 801 |
| 3h:55m | 797 |
| 4h:45m | 794 |
| 5h:30m | 795 |
| 6h:15m | 794 |
| 7h:00m | 794 |
| 7h:50m | 796 |
| 8h:35m | 804 |
| 9h:20m | 817 |
| 10h:05m | 873 |
| 10h:55m | 1042 |
| 11h:40m | 1556 |
| 12h:25m | 3258 |
| 13h:10m | 9329 |
| 14h:00m | 30984 |
| 14h:45m | 30938 |
| 15h:30m | 30928 |
| 16h:15m | 30924 |
| 17h:05m | 30923 |
| 17h:50m | 30924 |
| 18h:35m | 30924 |
| 19h:20m | 30925 |
| 20h:10m | 30926 |
| 20h:55m | 30926 |
| 21h:40m | 30925 |
| 22h:25m | 30924 |
| 23h:15m | 30923 |
| 24h:00m | 30923 |
| 24h:05m |  |
| 24h:50m |  |
| 25h:40m |  |
| 26h:25m |  |
| 27h:10m |  |
| 27h:55m |  |
| 28h:45m |  |
| 29h:30m |  |
| 30h:15m |  |
| 31h:00m |  |
| 31h:50m |  |
| 32h:35m |  |
| 33h:20m |  |
| 34h:05m |  |
| 34h:55m |  |
| 35h:40m |  |
| 36h:25m |  |
| 37h:10m |  |
| 38h:00m |  |
| 38h:45m |  |
| 39h:30m |  |
| 40h:15m |  |
| 41h:05m |  |
| 41h:50m |  |
| 42h:35m |  |
| 43h:20m |  |
| 44h:10m |  |
| 44h:55m |  |
| 45h:40m |  |
| 46h:25m |  |
| 47h:15m |  |
| 48h:00m |  |
| 48h:05m |  |
| 48h:50m |  |
| 49h:40m |  |
| 50h:25m |  |
| 51h:10m |  |
| 51h:55m |  |
| 52h:45m |  |
| 53h:30m |  |
| 54h:15m |  |
| 55h:00m |  |
| 55h:50m |  |
| 56h:35m |  |
| 57h:20m |  |
| 58h:05m |  |
| 58h:55m |  |
| 59h:40m |  |
| 60h:25m |  |
| 61h:10m |  |
| 62h:00m |  |
| 62h:45m |  |
| 63h:30m |  |
| 64h:15m |  |
| 65h:05m |  |
| 65h:50m |  |
| 66h:35m |  |
| 67h:20m |  |
| 68h:10m |  |
| 68h:55m |  |
| 69h:40m |  |
| 70h:25m |  |
| 71h:15m |  |
| 72h:00m |  |

E.Coli Garamycin 1MIC 2

| Time | EC 1MIC GARAE.3 |
| --- | --- |
| 0h:05m | 620 |
| 0h:50m | 623 |
| 1h:40m | 629 |
| 2h:25m | 628 |
| 3h:10m | 626 |
| 3h:55m | 628 |
| 4h:45m | 628 |
| 5h:30m | 628 |
| 6h:15m | 629 |
| 7h:00m | 630 |
| 7h:50m | 633 |
| 8h:35m | 636 |
| 9h:20m | 649 |
| 10h:05m | 681 |
| 10h:55m | 718 |
| 11h:40m | 762 |
| 12h:25m | 802 |
| 13h:10m | 834 |
| 14h:00m | 884 |
| 14h:45m | 936 |
| 15h:30m | 1004 |
| 16h:15m | 1117 |
| 17h:05m | 1372 |
| 17h:50m | 1955 |
| 18h:35m | 3505 |
| 19h:20m | 7722 |
| 20h:10m | 12066 |
| 20h:55m | 8033 |
| 21h:40m | 5758 |
| 22h:25m | 5067 |
| 23h:15m | 5295 |
| 24h:00m | 6074 |
| 24h:05m |  |
| 24h:50m |  |
| 25h:40m |  |
| 26h:25m |  |
| 27h:10m |  |
| 27h:55m |  |
| 28h:45m |  |
| 29h:30m |  |
| 30h:15m |  |
| 31h:00m |  |
| 31h:50m |  |
| 32h:35m |  |
| 33h:20m |  |
| 34h:05m |  |
| 34h:55m |  |
| 35h:40m |  |
| 36h:25m |  |
| 37h:10m |  |
| 38h:00m |  |
| 38h:45m |  |
| 39h:30m |  |
| 40h:15m |  |
| 41h:05m |  |
| 41h:50m |  |
| 42h:35m |  |
| 43h:20m |  |
| 44h:10m |  |
| 44h:55m |  |
| 45h:40m |  |
| 46h:25m |  |
| 47h:15m |  |
| 48h:00m |  |
| 48h:05m |  |
| 48h:50m |  |
| 49h:40m |  |
| 50h:25m |  |
| 51h:10m |  |
| 51h:55m |  |
| 52h:45m |  |
| 53h:30m |  |
| 54h:15m |  |
| 55h:00m |  |
| 55h:50m |  |
| 56h:35m |  |
| 57h:20m |  |
| 58h:05m |  |
| 58h:55m |  |
| 59h:40m |  |
| 60h:25m |  |
| 61h:10m |  |
| 62h:00m |  |
| 62h:45m |  |
| 63h:30m |  |
| 64h:15m |  |
| 65h:05m |  |
| 65h:50m |  |
| 66h:35m |  |
| 67h:20m |  |
| 68h:10m |  |
| 68h:55m |  |
| 69h:40m |  |
| 70h:25m |  |
| 71h:15m |  |
| 72h:00m |  |

E.Coli Garamycin 1MIC 3

| Time | EC 1MIC GARAE.5 |
| --- | --- |
| 0h:05m | 576 |
| 0h:50m | 571 |
| 1h:40m | 569 |
| 2h:25m | 571 |
| 3h:10m | 569 |
| 3h:55m | 572 |
| 4h:45m | 572 |
| 5h:30m | 574 |
| 6h:15m | 572 |
| 7h:00m | 571 |
| 7h:50m | 573 |
| 8h:35m | 575 |
| 9h:20m | 575 |
| 10h:05m | 581 |
| 10h:55m | 588 |
| 11h:40m | 606 |
| 12h:25m | 642 |
| 13h:10m | 728 |
| 14h:00m | 977 |
| 14h:45m | 1587 |
| 15h:30m | 3156 |
| 16h:15m | 7737 |
| 17h:05m | 19092 |
| 17h:50m | 30956 |
| 18h:35m | 30933 |
| 19h:20m | 30927 |
| 20h:10m | 30926 |
| 20h:55m | 30927 |
| 21h:40m | 30926 |
| 22h:25m | 30927 |
| 23h:15m | 30928 |
| 24h:00m | 30927 |
| 24h:05m |  |
| 24h:50m |  |
| 25h:40m |  |
| 26h:25m |  |
| 27h:10m |  |
| 27h:55m |  |
| 28h:45m |  |
| 29h:30m |  |
| 30h:15m |  |
| 31h:00m |  |
| 31h:50m |  |
| 32h:35m |  |
| 33h:20m |  |
| 34h:05m |  |
| 34h:55m |  |
| 35h:40m |  |
| 36h:25m |  |
| 37h:10m |  |
| 38h:00m |  |
| 38h:45m |  |
| 39h:30m |  |
| 40h:15m |  |
| 41h:05m |  |
| 41h:50m |  |
| 42h:35m |  |
| 43h:20m |  |
| 44h:10m |  |
| 44h:55m |  |
| 45h:40m |  |
| 46h:25m |  |
| 47h:15m |  |
| 48h:00m |  |
| 48h:05m |  |
| 48h:50m |  |
| 49h:40m |  |
| 50h:25m |  |
| 51h:10m |  |
| 51h:55m |  |
| 52h:45m |  |
| 53h:30m |  |
| 54h:15m |  |
| 55h:00m |  |
| 55h:50m |  |
| 56h:35m |  |
| 57h:20m |  |
| 58h:05m |  |
| 58h:55m |  |
| 59h:40m |  |
| 60h:25m |  |
| 61h:10m |  |
| 62h:00m |  |
| 62h:45m |  |
| 63h:30m |  |
| 64h:15m |  |
| 65h:05m |  |
| 65h:50m |  |
| 66h:35m |  |
| 67h:20m |  |
| 68h:10m |  |
| 68h:55m |  |
| 69h:40m |  |
| 70h:25m |  |
| 71h:15m |  |
| 72h:00m |  |

E.Coli Garamycin 1MIC 4

| Time | EC 1MIC GARAE.5 |
| --- | --- |
| 0h:05m | 738 |
| 0h:50m | 728 |
| 1h:40m | 728 |
| 2h:25m | 730 |
| 3h:10m | 729 |
| 3h:55m | 727 |
| 4h:45m | 723 |
| 5h:30m | 724 |
| 6h:15m | 720 |
| 7h:00m | 718 |
| 7h:50m | 720 |
| 8h:35m | 719 |
| 9h:20m | 725 |
| 10h:05m | 726 |
| 10h:55m | 729 |
| 11h:40m | 744 |
| 12h:25m | 763 |
| 13h:10m | 814 |
| 14h:00m | 958 |
| 14h:45m | 1282 |
| 15h:30m | 2054 |
| 16h:15m | 4118 |
| 17h:05m | 10639 |
| 17h:50m | 30011 |
| 18h:35m | 30954 |
| 19h:20m | 30938 |
| 20h:10m | 30933 |
| 20h:55m | 30934 |
| 21h:40m | 30934 |
| 22h:25m | 30933 |
| 23h:15m | 30934 |
| 24h:00m | 30933 |
| 24h:05m |  |
| 24h:50m |  |
| 25h:40m |  |
| 26h:25m |  |
| 27h:10m |  |
| 27h:55m |  |
| 28h:45m |  |
| 29h:30m |  |
| 30h:15m |  |
| 31h:00m |  |
| 31h:50m |  |
| 32h:35m |  |
| 33h:20m |  |
| 34h:05m |  |
| 34h:55m |  |
| 35h:40m |  |
| 36h:25m |  |
| 37h:10m |  |
| 38h:00m |  |
| 38h:45m |  |
| 39h:30m |  |
| 40h:15m |  |
| 41h:05m |  |
| 41h:50m |  |
| 42h:35m |  |
| 43h:20m |  |
| 44h:10m |  |
| 44h:55m |  |
| 45h:40m |  |
| 46h:25m |  |
| 47h:15m |  |
| 48h:00m |  |
| 48h:05m |  |
| 48h:50m |  |
| 49h:40m |  |
| 50h:25m |  |
| 51h:10m |  |
| 51h:55m |  |
| 52h:45m |  |
| 53h:30m |  |
| 54h:15m |  |
| 55h:00m |  |
| 55h:50m |  |
| 56h:35m |  |
| 57h:20m |  |
| 58h:05m |  |
| 58h:55m |  |
| 59h:40m |  |
| 60h:25m |  |
| 61h:10m |  |
| 62h:00m |  |
| 62h:45m |  |
| 63h:30m |  |
| 64h:15m |  |
| 65h:05m |  |
| 65h:50m |  |
| 66h:35m |  |
| 67h:20m |  |
| 68h:10m |  |
| 68h:55m |  |
| 69h:40m |  |
| 70h:25m |  |
| 71h:15m |  |
| 72h:00m |  |

E.Coli Garamycin 1MIC 5

| Time | EC 1MIC GARAE.4 |
| --- | --- |
| 0h:05m | 793 |
| 0h:50m | 771 |
| 1h:40m | 763 |
| 2h:25m | 761 |
| 3h:10m | 766 |
| 3h:55m | 758 |
| 4h:45m | 757 |
| 5h:30m | 757 |
| 6h:15m | 756 |
| 7h:00m | 757 |
| 7h:50m | 761 |
| 8h:35m | 763 |
| 9h:20m | 778 |
| 10h:05m | 848 |
| 10h:55m | 1146 |
| 11h:40m | 2008 |
| 12h:25m | 5614 |
| 13h:10m | 19191 |
| 14h:00m | 30959 |
| 14h:45m | 30932 |
| 15h:30m | 30927 |
| 16h:15m | 30927 |
| 17h:05m | 30926 |
| 17h:50m | 30926 |
| 18h:35m | 30927 |
| 19h:20m | 30925 |
| 20h:10m | 30926 |
| 20h:55m | 30925 |
| 21h:40m | 30925 |
| 22h:25m | 30924 |
| 23h:15m | 30926 |
| 24h:00m | 30925 |
| 24h:05m |  |
| 24h:50m |  |
| 25h:40m |  |
| 26h:25m |  |
| 27h:10m |  |
| 27h:55m |  |
| 28h:45m |  |
| 29h:30m |  |
| 30h:15m |  |
| 31h:00m |  |
| 31h:50m |  |
| 32h:35m |  |
| 33h:20m |  |
| 34h:05m |  |
| 34h:55m |  |
| 35h:40m |  |
| 36h:25m |  |
| 37h:10m |  |
| 38h:00m |  |
| 38h:45m |  |
| 39h:30m |  |
| 40h:15m |  |
| 41h:05m |  |
| 41h:50m |  |
| 42h:35m |  |
| 43h:20m |  |
| 44h:10m |  |
| 44h:55m |  |
| 45h:40m |  |
| 46h:25m |  |
| 47h:15m |  |
| 48h:00m |  |
| 48h:05m |  |
| 48h:50m |  |
| 49h:40m |  |
| 50h:25m |  |
| 51h:10m |  |
| 51h:55m |  |
| 52h:45m |  |
| 53h:30m |  |
| 54h:15m |  |
| 55h:00m |  |
| 55h:50m |  |
| 56h:35m |  |
| 57h:20m |  |
| 58h:05m |  |
| 58h:55m |  |
| 59h:40m |  |
| 60h:25m |  |
| 61h:10m |  |
| 62h:00m |  |
| 62h:45m |  |
| 63h:30m |  |
| 64h:15m |  |
| 65h:05m |  |
| 65h:50m |  |
| 66h:35m |  |
| 67h:20m |  |
| 68h:10m |  |
| 68h:55m |  |
| 69h:40m |  |
| 70h:25m |  |
| 71h:15m |  |
| 72h:00m |  |

E.Coli Garamycin 1MIC 6

| Time | EC 1MIC GARAE.4 |
| --- | --- |
| 0h:05m | 651 |
| 0h:50m | 635 |
| 1h:40m | 621 |
| 2h:25m | 617 |
| 3h:10m | 614 |
| 3h:55m | 611 |
| 4h:45m | 610 |
| 5h:30m | 609 |
| 6h:15m | 609 |
| 7h:00m | 610 |
| 7h:50m | 612 |
| 8h:35m | 616 |
| 9h:20m | 633 |
| 10h:05m | 704 |
| 10h:55m | 1158 |
| 11h:40m | 2804 |
| 12h:25m | 8588 |
| 13h:10m | 28770 |
| 14h:00m | 30953 |
| 14h:45m | 30934 |
| 15h:30m | 30930 |
| 16h:15m | 30929 |
| 17h:05m | 30929 |
| 17h:50m | 30929 |
| 18h:35m | 30930 |
| 19h:20m | 30929 |
| 20h:10m | 30928 |
| 20h:55m | 30928 |
| 21h:40m | 30928 |
| 22h:25m | 30929 |
| 23h:15m | 30929 |
| 24h:00m | 30929 |
| 24h:05m |  |
| 24h:50m |  |
| 25h:40m |  |
| 26h:25m |  |
| 27h:10m |  |
| 27h:55m |  |
| 28h:45m |  |
| 29h:30m |  |
| 30h:15m |  |
| 31h:00m |  |
| 31h:50m |  |
| 32h:35m |  |
| 33h:20m |  |
| 34h:05m |  |
| 34h:55m |  |
| 35h:40m |  |
| 36h:25m |  |
| 37h:10m |  |
| 38h:00m |  |
| 38h:45m |  |
| 39h:30m |  |
| 40h:15m |  |
| 41h:05m |  |
| 41h:50m |  |
| 42h:35m |  |
| 43h:20m |  |
| 44h:10m |  |
| 44h:55m |  |
| 45h:40m |  |
| 46h:25m |  |
| 47h:15m |  |
| 48h:00m |  |
| 48h:05m |  |
| 48h:50m |  |
| 49h:40m |  |
| 50h:25m |  |
| 51h:10m |  |
| 51h:55m |  |
| 52h:45m |  |
| 53h:30m |  |
| 54h:15m |  |
| 55h:00m |  |
| 55h:50m |  |
| 56h:35m |  |
| 57h:20m |  |
| 58h:05m |  |
| 58h:55m |  |
| 59h:40m |  |
| 60h:25m |  |
| 61h:10m |  |
| 62h:00m |  |
| 62h:45m |  |
| 63h:30m |  |
| 64h:15m |  |
| 65h:05m |  |
| 65h:50m |  |
| 66h:35m |  |
| 67h:20m |  |
| 68h:10m |  |
| 68h:55m |  |
| 69h:40m |  |
| 70h:25m |  |
| 71h:15m |  |
| 72h:00m |  |

E.Coli Garamycin 1MIC 7

| Time | EC 1MIC GARAE.W |
| --- | --- |
| 0h:05m | 312 |
| 0h:50m | 306 |
| 1h:40m | 309 |
| 2h:25m | 312 |
| 3h:10m | 315 |
| 3h:55m | 316 |
| 4h:45m | 319 |
| 5h:30m | 319 |
| 6h:15m | 321 |
| 7h:00m | 323 |
| 7h:50m | 323 |
| 8h:35m | 323 |
| 9h:20m | 325 |
| 10h:05m | 325 |
| 10h:55m | 324 |
| 11h:40m | 327 |
| 12h:25m | 327 |
| 13h:10m | 330 |
| 14h:00m | 334 |
| 14h:45m | 340 |
| 15h:30m | 348 |
| 16h:15m | 382 |
| 17h:05m | 488 |
| 17h:50m | 741 |
| 18h:35m | 1357 |
| 19h:20m | 2589 |
| 20h:10m | 5441 |
| 20h:55m | 10243 |
| 21h:40m | 19867 |
| 22h:25m | 31020 |
| 23h:15m | 31020 |
| 24h:00m | 31020 |
| 24h:05m |  |
| 24h:50m |  |
| 25h:40m |  |
| 26h:25m |  |
| 27h:10m |  |
| 27h:55m |  |
| 28h:45m |  |
| 29h:30m |  |
| 30h:15m |  |
| 31h:00m |  |
| 31h:50m |  |
| 32h:35m |  |
| 33h:20m |  |
| 34h:05m |  |
| 34h:55m |  |
| 35h:40m |  |
| 36h:25m |  |
| 37h:10m |  |
| 38h:00m |  |
| 38h:45m |  |
| 39h:30m |  |
| 40h:15m |  |
| 41h:05m |  |
| 41h:50m |  |
| 42h:35m |  |
| 43h:20m |  |
| 44h:10m |  |
| 44h:55m |  |
| 45h:40m |  |
| 46h:25m |  |
| 47h:15m |  |
| 48h:00m |  |
| 48h:05m |  |
| 48h:50m |  |
| 49h:40m |  |
| 50h:25m |  |
| 51h:10m |  |
| 51h:55m |  |
| 52h:45m |  |
| 53h:30m |  |
| 54h:15m |  |
| 55h:00m |  |
| 55h:50m |  |
| 56h:35m |  |
| 57h:20m |  |
| 58h:05m |  |
| 58h:55m |  |
| 59h:40m |  |
| 60h:25m |  |
| 61h:10m |  |
| 62h:00m |  |
| 62h:45m |  |
| 63h:30m |  |
| 64h:15m |  |
| 65h:05m |  |
| 65h:50m |  |
| 66h:35m |  |
| 67h:20m |  |
| 68h:10m |  |
| 68h:55m |  |
| 69h:40m |  |
| 70h:25m |  |
| 71h:15m |  |
| 72h:00m |  |

E.Coli Garamycin 1MIC 8

| Time | EC 1MIC GARAE.W |
| --- | --- |
| 0h:05m | 349 |
| 0h:50m | 352 |
| 1h:40m | 354 |
| 2h:25m | 354 |
| 3h:10m | 356 |
| 3h:55m | 356 |
| 4h:45m | 358 |
| 5h:30m | 360 |
| 6h:15m | 362 |
| 7h:00m | 362 |
| 7h:50m | 364 |
| 8h:35m | 366 |
| 9h:20m | 368 |
| 10h:05m | 369 |
| 10h:55m | 370 |
| 11h:40m | 371 |
| 12h:25m | 372 |
| 13h:10m | 375 |
| 14h:00m | 380 |
| 14h:45m | 393 |
| 15h:30m | 416 |
| 16h:15m | 492 |
| 17h:05m | 684 |
| 17h:50m | 1305 |
| 18h:35m | 2566 |
| 19h:20m | 5289 |
| 20h:10m | 10924 |
| 20h:55m | 21909 |
| 21h:40m | 31021 |
| 22h:25m | 31001 |
| 23h:15m | 30997 |
| 24h:00m | 30998 |
| 24h:05m |  |
| 24h:50m |  |
| 25h:40m |  |
| 26h:25m |  |
| 27h:10m |  |
| 27h:55m |  |
| 28h:45m |  |
| 29h:30m |  |
| 30h:15m |  |
| 31h:00m |  |
| 31h:50m |  |
| 32h:35m |  |
| 33h:20m |  |
| 34h:05m |  |
| 34h:55m |  |
| 35h:40m |  |
| 36h:25m |  |
| 37h:10m |  |
| 38h:00m |  |
| 38h:45m |  |
| 39h:30m |  |
| 40h:15m |  |
| 41h:05m |  |
| 41h:50m |  |
| 42h:35m |  |
| 43h:20m |  |
| 44h:10m |  |
| 44h:55m |  |
| 45h:40m |  |
| 46h:25m |  |
| 47h:15m |  |
| 48h:00m |  |
| 48h:05m |  |
| 48h:50m |  |
| 49h:40m |  |
| 50h:25m |  |
| 51h:10m |  |
| 51h:55m |  |
| 52h:45m |  |
| 53h:30m |  |
| 54h:15m |  |
| 55h:00m |  |
| 55h:50m |  |
| 56h:35m |  |
| 57h:20m |  |
| 58h:05m |  |
| 58h:55m |  |
| 59h:40m |  |
| 60h:25m |  |
| 61h:10m |  |
| 62h:00m |  |
| 62h:45m |  |
| 63h:30m |  |
| 64h:15m |  |
| 65h:05m |  |
| 65h:50m |  |
| 66h:35m |  |
| 67h:20m |  |
| 68h:10m |  |
| 68h:55m |  |
| 69h:40m |  |
| 70h:25m |  |
| 71h:15m |  |
| 72h:00m |  |

E.Coli Garamycin 4MIC 1

| Time | EC 4MIC GARA E.3 |
| --- | --- |
| 0h:05m | 570 |
| 0h:50m | 579 |
| 1h:40m | 579 |
| 2h:25m | 578 |
| 3h:10m | 580 |
| 3h:55m | 578 |
| 4h:45m | 577 |
| 5h:30m | 578 |
| 6h:15m | 578 |
| 7h:00m | 577 |
| 7h:50m | 577 |
| 8h:35m | 580 |
| 9h:20m | 581 |
| 10h:05m | 578 |
| 10h:55m | 581 |
| 11h:40m | 581 |
| 12h:25m | 581 |
| 13h:10m | 585 |
| 14h:00m | 581 |
| 14h:45m | 582 |
| 15h:30m | 587 |
| 16h:15m | 583 |
| 17h:05m | 583 |
| 17h:50m | 583 |
| 18h:35m | 586 |
| 19h:20m | 589 |
| 20h:10m | 585 |
| 20h:55m | 586 |
| 21h:40m | 587 |
| 22h:25m | 586 |
| 23h:15m | 585 |
| 24h:00m | 584 |
| 24h:05m | 542 |
| 24h:50m | 541 |
| 25h:40m | 541 |
| 26h:25m | 542 |
| 27h:10m | 542 |
| 27h:55m | 543 |
| 28h:45m | 543 |
| 29h:30m | 542 |
| 30h:15m | 543 |
| 31h:00m | 542 |
| 31h:50m | 543 |
| 32h:35m | 542 |
| 33h:20m | 544 |
| 34h:05m | 542 |
| 34h:55m | 544 |
| 35h:40m | 543 |
| 36h:25m | 544 |
| 37h:10m | 543 |
| 38h:00m | 541 |
| 38h:45m | 542 |
| 39h:30m | 545 |
| 40h:15m | 543 |
| 41h:05m | 544 |
| 41h:50m | 544 |
| 42h:35m | 541 |
| 43h:20m | 541 |
| 44h:10m | 542 |
| 44h:55m | 542 |
| 45h:40m | 544 |
| 46h:25m | 541 |
| 47h:15m | 543 |
| 48h:00m | 544 |
| 48h:05m | 608 |
| 48h:50m | 610 |
| 49h:40m | 608 |
| 50h:25m | 603 |
| 51h:10m | 602 |
| 51h:55m | 606 |
| 52h:45m | 606 |
| 53h:30m | 605 |
| 54h:15m | 605 |
| 55h:00m | 608 |
| 55h:50m | 605 |
| 56h:35m | 605 |
| 57h:20m | 604 |
| 58h:05m | 603 |
| 58h:55m | 607 |
| 59h:40m | 606 |
| 60h:25m | 606 |
| 61h:10m | 603 |
| 62h:00m | 603 |
| 62h:45m | 603 |
| 63h:30m | 605 |
| 64h:15m | 604 |
| 65h:05m | 603 |
| 65h:50m | 602 |
| 66h:35m | 604 |
| 67h:20m | 605 |
| 68h:10m | 603 |
| 68h:55m | 604 |
| 69h:40m | 602 |
| 70h:25m | 602 |
| 71h:15m | 601 |
| 72h:00m | 601 |

E.Coli Garamycin 4MIC 2

| Time | EC 4MIC GARA E.3 |
| --- | --- |
| 0h:05m | 737 |
| 0h:50m | 742 |
| 1h:40m | 747 |
| 2h:25m | 745 |
| 3h:10m | 745 |
| 3h:55m | 743 |
| 4h:45m | 745 |
| 5h:30m | 744 |
| 6h:15m | 747 |
| 7h:00m | 743 |
| 7h:50m | 744 |
| 8h:35m | 745 |
| 9h:20m | 745 |
| 10h:05m | 743 |
| 10h:55m | 747 |
| 11h:40m | 747 |
| 12h:25m | 745 |
| 13h:10m | 749 |
| 14h:00m | 747 |
| 14h:45m | 748 |
| 15h:30m | 751 |
| 16h:15m | 753 |
| 17h:05m | 755 |
| 17h:50m | 760 |
| 18h:35m | 762 |
| 19h:20m | 762 |
| 20h:10m | 761 |
| 20h:55m | 766 |
| 21h:40m | 769 |
| 22h:25m | 771 |
| 23h:15m | 773 |
| 24h:00m | 774 |
| 24h:05m | 651 |
| 24h:50m | 657 |
| 25h:40m | 657 |
| 26h:25m | 656 |
| 27h:10m | 657 |
| 27h:55m | 657 |
| 28h:45m | 655 |
| 29h:30m | 656 |
| 30h:15m | 655 |
| 31h:00m | 655 |
| 31h:50m | 654 |
| 32h:35m | 655 |
| 33h:20m | 656 |
| 34h:05m | 657 |
| 34h:55m | 662 |
| 35h:40m | 668 |
| 36h:25m | 673 |
| 37h:10m | 677 |
| 38h:00m | 689 |
| 38h:45m | 707 |
| 39h:30m | 739 |
| 40h:15m | 782 |
| 41h:05m | 873 |
| 41h:50m | 1091 |
| 42h:35m | 1266 |
| 43h:20m | 1625 |
| 44h:10m | 2449 |
| 44h:55m | 3984 |
| 45h:40m | 7312 |
| 46h:25m | 18584 |
| 47h:15m | 31022 |
| 48h:00m | 31014 |
| 48h:05m |  |
| 48h:50m |  |
| 49h:40m |  |
| 50h:25m |  |
| 51h:10m |  |
| 51h:55m |  |
| 52h:45m |  |
| 53h:30m |  |
| 54h:15m |  |
| 55h:00m |  |
| 55h:50m |  |
| 56h:35m |  |
| 57h:20m |  |
| 58h:05m |  |
| 58h:55m |  |
| 59h:40m |  |
| 60h:25m |  |
| 61h:10m |  |
| 62h:00m |  |
| 62h:45m |  |
| 63h:30m |  |
| 64h:15m |  |
| 65h:05m |  |
| 65h:50m |  |
| 66h:35m |  |
| 67h:20m |  |
| 68h:10m |  |
| 68h:55m |  |
| 69h:40m |  |
| 70h:25m |  |
| 71h:15m |  |
| 72h:00m |  |

E.Coli Garamycin 4MIC 3

| Time | EC 4MIC GARA E.5 |
| --- | --- |
| 0h:05m | 468 |
| 0h:50m | 470 |
| 1h:40m | 468 |
| 2h:25m | 468 |
| 3h:10m | 469 |
| 3h:55m | 471 |
| 4h:45m | 472 |
| 5h:30m | 471 |
| 6h:15m | 474 |
| 7h:00m | 473 |
| 7h:50m | 475 |
| 8h:35m | 477 |
| 9h:20m | 477 |
| 10h:05m | 483 |
| 10h:55m | 485 |
| 11h:40m | 486 |
| 12h:25m | 487 |
| 13h:10m | 489 |
| 14h:00m | 491 |
| 14h:45m | 493 |
| 15h:30m | 494 |
| 16h:15m | 493 |
| 17h:05m | 495 |
| 17h:50m | 497 |
| 18h:35m | 496 |
| 19h:20m | 498 |
| 20h:10m | 499 |
| 20h:55m | 500 |
| 21h:40m | 500 |
| 22h:25m | 501 |
| 23h:15m | 501 |
| 24h:00m | 503 |
| 24h:05m | 503 |
| 24h:50m | 503 |
| 25h:40m | 503 |
| 26h:25m | 503 |
| 27h:10m | 503 |
| 27h:55m | 503 |
| 28h:45m | 503 |
| 29h:30m | 503 |
| 30h:15m | 503 |
| 31h:00m | 503 |
| 31h:50m | 503 |
| 32h:35m | 503 |
| 33h:20m | 503 |
| 34h:05m | 503 |
| 34h:55m | 503 |
| 35h:40m | 503 |
| 36h:25m | 503 |
| 37h:10m | 503 |
| 38h:00m | 503 |
| 38h:45m | 503 |
| 39h:30m | 503 |
| 40h:15m | 503 |
| 41h:05m | 503 |
| 41h:50m | 503 |
| 42h:35m | 503 |
| 43h:20m | 503 |
| 44h:10m | 503 |
| 44h:55m | 503 |
| 45h:40m | 503 |
| 46h:25m | 503 |
| 47h:15m | 503 |
| 48h:00m | 503 |
| 48h:05m | 512 |
| 48h:50m | 520 |
| 49h:40m | 523 |
| 50h:25m | 524 |
| 51h:10m | 526 |
| 51h:55m | 525 |
| 52h:45m | 524 |
| 53h:30m | 526 |
| 54h:15m | 527 |
| 55h:00m | 530 |
| 55h:50m | 527 |
| 56h:35m | 525 |
| 57h:20m | 526 |
| 58h:05m | 525 |
| 58h:55m | 523 |
| 59h:40m | 519 |
| 60h:25m | 521 |
| 61h:10m | 521 |
| 62h:00m | 520 |
| 62h:45m | 516 |
| 63h:30m | 518 |
| 64h:15m | 515 |
| 65h:05m | 523 |
| 65h:50m | 526 |
| 66h:35m | 528 |
| 67h:20m | 528 |
| 68h:10m | 529 |
| 68h:55m | 529 |
| 69h:40m | 529 |
| 70h:25m | 525 |
| 71h:15m | 527 |
| 72h:00m | 529 |

E.Coli Garamycin 4MIC 4

| Time | EC 4MIC GARA E.5 |
| --- | --- |
| 0h:05m | 476 |
| 0h:50m | 481 |
| 1h:40m | 477 |
| 2h:25m | 474 |
| 3h:10m | 471 |
| 3h:55m | 472 |
| 4h:45m | 472 |
| 5h:30m | 471 |
| 6h:15m | 471 |
| 7h:00m | 467 |
| 7h:50m | 468 |
| 8h:35m | 469 |
| 9h:20m | 471 |
| 10h:05m | 473 |
| 10h:55m | 471 |
| 11h:40m | 471 |
| 12h:25m | 472 |
| 13h:10m | 472 |
| 14h:00m | 470 |
| 14h:45m | 472 |
| 15h:30m | 473 |
| 16h:15m | 473 |
| 17h:05m | 473 |
| 17h:50m | 474 |
| 18h:35m | 474 |
| 19h:20m | 476 |
| 20h:10m | 476 |
| 20h:55m | 478 |
| 21h:40m | 479 |
| 22h:25m | 478 |
| 23h:15m | 483 |
| 24h:00m | 484 |
| 24h:05m | 484 |
| 24h:50m | 484 |
| 25h:40m | 484 |
| 26h:25m | 484 |
| 27h:10m | 484 |
| 27h:55m | 484 |
| 28h:45m | 484 |
| 29h:30m | 484 |
| 30h:15m | 484 |
| 31h:00m | 484 |
| 31h:50m | 484 |
| 32h:35m | 484 |
| 33h:20m | 484 |
| 34h:05m | 484 |
| 34h:55m | 484 |
| 35h:40m | 484 |
| 36h:25m | 484 |
| 37h:10m | 484 |
| 38h:00m | 484 |
| 38h:45m | 484 |
| 39h:30m | 484 |
| 40h:15m | 484 |
| 41h:05m | 484 |
| 41h:50m | 484 |
| 42h:35m | 484 |
| 43h:20m | 484 |
| 44h:10m | 484 |
| 44h:55m | 484 |
| 45h:40m | 484 |
| 46h:25m | 484 |
| 47h:15m | 484 |
| 48h:00m | 484 |
| 48h:05m | 518 |
| 48h:50m | 518 |
| 49h:40m | 515 |
| 50h:25m | 517 |
| 51h:10m | 515 |
| 51h:55m | 516 |
| 52h:45m | 519 |
| 53h:30m | 515 |
| 54h:15m | 521 |
| 55h:00m | 520 |
| 55h:50m | 519 |
| 56h:35m | 519 |
| 57h:20m | 518 |
| 58h:05m | 517 |
| 58h:55m | 519 |
| 59h:40m | 514 |
| 60h:25m | 514 |
| 61h:10m | 514 |
| 62h:00m | 514 |
| 62h:45m | 513 |
| 63h:30m | 513 |
| 64h:15m | 513 |
| 65h:05m | 518 |
| 65h:50m | 519 |
| 66h:35m | 519 |
| 67h:20m | 515 |
| 68h:10m | 515 |
| 68h:55m | 517 |
| 69h:40m | 518 |
| 70h:25m | 519 |
| 71h:15m | 517 |
| 72h:00m | 515 |

E.Coli Garamycin 4MIC 5

| Time | EC 4MIC GARA E.4 |
| --- | --- |
| 0h:05m | 796 |
| 0h:50m | 778 |
| 1h:40m | 777 |
| 2h:25m | 772 |
| 3h:10m | 767 |
| 3h:55m | 764 |
| 4h:45m | 763 |
| 5h:30m | 761 |
| 6h:15m | 759 |
| 7h:00m | 759 |
| 7h:50m | 757 |
| 8h:35m | 756 |
| 9h:20m | 755 |
| 10h:05m | 752 |
| 10h:55m | 753 |
| 11h:40m | 753 |
| 12h:25m | 755 |
| 13h:10m | 760 |
| 14h:00m | 762 |
| 14h:45m | 767 |
| 15h:30m | 768 |
| 16h:15m | 770 |
| 17h:05m | 770 |
| 17h:50m | 771 |
| 18h:35m | 773 |
| 19h:20m | 773 |
| 20h:10m | 774 |
| 20h:55m | 776 |
| 21h:40m | 778 |
| 22h:25m | 786 |
| 23h:15m | 792 |
| 24h:00m | 815 |
| 24h:05m | 1062 |
| 24h:50m | 1103 |
| 25h:40m | 1239 |
| 26h:25m | 1553 |
| 27h:10m | 2130 |
| 27h:55m | 3211 |
| 28h:45m | 6254 |
| 29h:30m | 11658 |
| 30h:15m | 21717 |
| 31h:00m | 30991 |
| 31h:50m | 30945 |
| 32h:35m | 30930 |
| 33h:20m | 30927 |
| 34h:05m | 30929 |
| 34h:55m | 30927 |
| 35h:40m | 30927 |
| 36h:25m | 30928 |
| 37h:10m | 30927 |
| 38h:00m | 30927 |
| 38h:45m | 30928 |
| 39h:30m | 30929 |
| 40h:15m | 30931 |
| 41h:05m | 30932 |
| 41h:50m | 30931 |
| 42h:35m | 30932 |
| 43h:20m | 30932 |
| 44h:10m | 30931 |
| 44h:55m | 30930 |
| 45h:40m | 30929 |
| 46h:25m | 30931 |
| 47h:15m | 30930 |
| 48h:00m | 30932 |
| 48h:05m |  |
| 48h:50m |  |
| 49h:40m |  |
| 50h:25m |  |
| 51h:10m |  |
| 51h:55m |  |
| 52h:45m |  |
| 53h:30m |  |
| 54h:15m |  |
| 55h:00m |  |
| 55h:50m |  |
| 56h:35m |  |
| 57h:20m |  |
| 58h:05m |  |
| 58h:55m |  |
| 59h:40m |  |
| 60h:25m |  |
| 61h:10m |  |
| 62h:00m |  |
| 62h:45m |  |
| 63h:30m |  |
| 64h:15m |  |
| 65h:05m |  |
| 65h:50m |  |
| 66h:35m |  |
| 67h:20m |  |
| 68h:10m |  |
| 68h:55m |  |
| 69h:40m |  |
| 70h:25m |  |
| 71h:15m |  |
| 72h:00m |  |

E.Coli Garamycin 4MIC 6

| Time | EC 4MIC GARA E.4 |
| --- | --- |
| 0h:05m | 596 |
| 0h:50m | 583 |
| 1h:40m | 577 |
| 2h:25m | 575 |
| 3h:10m | 573 |
| 3h:55m | 571 |
| 4h:45m | 570 |
| 5h:30m | 567 |
| 6h:15m | 565 |
| 7h:00m | 563 |
| 7h:50m | 563 |
| 8h:35m | 562 |
| 9h:20m | 561 |
| 10h:05m | 560 |
| 10h:55m | 559 |
| 11h:40m | 556 |
| 12h:25m | 557 |
| 13h:10m | 557 |
| 14h:00m | 555 |
| 14h:45m | 555 |
| 15h:30m | 554 |
| 16h:15m | 553 |
| 17h:05m | 552 |
| 17h:50m | 552 |
| 18h:35m | 552 |
| 19h:20m | 553 |
| 20h:10m | 551 |
| 20h:55m | 555 |
| 21h:40m | 572 |
| 22h:25m | 610 |
| 23h:15m | 724 |
| 24h:00m | 897 |
| 24h:05m | 1337 |
| 24h:50m | 2490 |
| 25h:40m | 4901 |
| 26h:25m | 8723 |
| 27h:10m | 16548 |
| 27h:55m | 27575 |
| 28h:45m | 30976 |
| 29h:30m | 30963 |
| 30h:15m | 30960 |
| 31h:00m | 30959 |
| 31h:50m | 30958 |
| 32h:35m | 30958 |
| 33h:20m | 30958 |
| 34h:05m | 30958 |
| 34h:55m | 30958 |
| 35h:40m | 30961 |
| 36h:25m | 30961 |
| 37h:10m | 30963 |
| 38h:00m | 30963 |
| 38h:45m | 30966 |
| 39h:30m | 30968 |
| 40h:15m | 30969 |
| 41h:05m | 30969 |
| 41h:50m | 30969 |
| 42h:35m | 30969 |
| 43h:20m | 30969 |
| 44h:10m | 30968 |
| 44h:55m | 30968 |
| 45h:40m | 30968 |
| 46h:25m | 30969 |
| 47h:15m | 30970 |
| 48h:00m | 30970 |
| 48h:05m |  |
| 48h:50m |  |
| 49h:40m |  |
| 50h:25m |  |
| 51h:10m |  |
| 51h:55m |  |
| 52h:45m |  |
| 53h:30m |  |
| 54h:15m |  |
| 55h:00m |  |
| 55h:50m |  |
| 56h:35m |  |
| 57h:20m |  |
| 58h:05m |  |
| 58h:55m |  |
| 59h:40m |  |
| 60h:25m |  |
| 61h:10m |  |
| 62h:00m |  |
| 62h:45m |  |
| 63h:30m |  |
| 64h:15m |  |
| 65h:05m |  |
| 65h:50m |  |
| 66h:35m |  |
| 67h:20m |  |
| 68h:10m |  |
| 68h:55m |  |
| 69h:40m |  |
| 70h:25m |  |
| 71h:15m |  |
| 72h:00m |  |

E.Coli Garamycin 4MIC 7

| Time | EC 4MIC GARA E.W |
| --- | --- |
| 0h:05m | 285 |
| 0h:50m | 283 |
| 1h:40m | 286 |
| 2h:25m | 288 |
| 3h:10m | 289 |
| 3h:55m | 292 |
| 4h:45m | 292 |
| 5h:30m | 292 |
| 6h:15m | 294 |
| 7h:00m | 294 |
| 7h:50m | 293 |
| 8h:35m | 295 |
| 9h:20m | 295 |
| 10h:05m | 295 |
| 10h:55m | 295 |
| 11h:40m | 295 |
| 12h:25m | 295 |
| 13h:10m | 294 |
| 14h:00m | 293 |
| 14h:45m | 295 |
| 15h:30m | 295 |
| 16h:15m | 296 |
| 17h:05m | 295 |
| 17h:50m | 296 |
| 18h:35m | 295 |
| 19h:20m | 298 |
| 20h:10m | 301 |
| 20h:55m | 304 |
| 21h:40m | 323 |
| 22h:25m | 356 |
| 23h:15m | 504 |
| 24h:00m | 868 |
| 24h:05m | 3550 |
| 24h:50m | 6161 |
| 25h:40m | 11408 |
| 26h:25m | 24254 |
| 27h:10m | 30975 |
| 27h:55m | 30943 |
| 28h:45m | 30937 |
| 29h:30m | 30937 |
| 30h:15m | 30936 |
| 31h:00m | 30936 |
| 31h:50m | 30936 |
| 32h:35m | 30937 |
| 33h:20m | 30938 |
| 34h:05m | 30938 |
| 34h:55m | 30938 |
| 35h:40m | 30937 |
| 36h:25m | 30938 |
| 37h:10m | 30939 |
| 38h:00m | 30939 |
| 38h:45m | 30940 |
| 39h:30m | 30938 |
| 40h:15m | 30939 |
| 41h:05m | 30940 |
| 41h:50m | 30940 |
| 42h:35m | 30940 |
| 43h:20m | 30940 |
| 44h:10m | 30940 |
| 44h:55m | 30939 |
| 45h:40m | 30940 |
| 46h:25m | 30939 |
| 47h:15m | 30944 |
| 48h:00m | 30945 |
| 48h:05m |  |
| 48h:50m |  |
| 49h:40m |  |
| 50h:25m |  |
| 51h:10m |  |
| 51h:55m |  |
| 52h:45m |  |
| 53h:30m |  |
| 54h:15m |  |
| 55h:00m |  |
| 55h:50m |  |
| 56h:35m |  |
| 57h:20m |  |
| 58h:05m |  |
| 58h:55m |  |
| 59h:40m |  |
| 60h:25m |  |
| 61h:10m |  |
| 62h:00m |  |
| 62h:45m |  |
| 63h:30m |  |
| 64h:15m |  |
| 65h:05m |  |
| 65h:50m |  |
| 66h:35m |  |
| 67h:20m |  |
| 68h:10m |  |
| 68h:55m |  |
| 69h:40m |  |
| 70h:25m |  |
| 71h:15m |  |
| 72h:00m |  |

E.Coli Garamycin 4MIC 8

| Time | EC 4MIC GARA E.W |
| --- | --- |
| 0h:05m | 972 |
| 0h:50m | 994 |
| 1h:40m | 992 |
| 2h:25m | 999 |
| 3h:10m | 997 |
| 3h:55m | 990 |
| 4h:45m | 989 |
| 5h:30m | 995 |
| 6h:15m | 995 |
| 7h:00m | 990 |
| 7h:50m | 991 |
| 8h:35m | 997 |
| 9h:20m | 1004 |
| 10h:05m | 1004 |
| 10h:55m | 1005 |
| 11h:40m | 1003 |
| 12h:25m | 1002 |
| 13h:10m | 1004 |
| 14h:00m | 1006 |
| 14h:45m | 1006 |
| 15h:30m | 1003 |
| 16h:15m | 1006 |
| 17h:05m | 1002 |
| 17h:50m | 1005 |
| 18h:35m | 1006 |
| 19h:20m | 1003 |
| 20h:10m | 1004 |
| 20h:55m | 1003 |
| 21h:40m | 1002 |
| 22h:25m | 1006 |
| 23h:15m | 1004 |
| 24h:00m | 1042 |
| 24h:05m | 672 |
| 24h:50m | 675 |
| 25h:40m | 678 |
| 26h:25m | 679 |
| 27h:10m | 678 |
| 27h:55m | 680 |
| 28h:45m | 677 |
| 29h:30m | 676 |
| 30h:15m | 675 |
| 31h:00m | 675 |
| 31h:50m | 677 |
| 32h:35m | 675 |
| 33h:20m | 676 |
| 34h:05m | 675 |
| 34h:55m | 675 |
| 35h:40m | 675 |
| 36h:25m | 675 |
| 37h:10m | 676 |
| 38h:00m | 675 |
| 38h:45m | 675 |
| 39h:30m | 674 |
| 40h:15m | 673 |
| 41h:05m | 672 |
| 41h:50m | 671 |
| 42h:35m | 673 |
| 43h:20m | 671 |
| 44h:10m | 671 |
| 44h:55m | 672 |
| 45h:40m | 672 |
| 46h:25m | 672 |
| 47h:15m | 674 |
| 48h:00m | 670 |
| 48h:05m | 365 |
| 48h:50m | 367 |
| 49h:40m | 366 |
| 50h:25m | 365 |
| 51h:10m | 367 |
| 51h:55m | 365 |
| 52h:45m | 368 |
| 53h:30m | 367 |
| 54h:15m | 367 |
| 55h:00m | 365 |
| 55h:50m | 365 |
| 56h:35m | 366 |
| 57h:20m | 366 |
| 58h:05m | 364 |
| 58h:55m | 365 |
| 59h:40m | 364 |
| 60h:25m | 363 |
| 61h:10m | 364 |
| 62h:00m | 364 |
| 62h:45m | 363 |
| 63h:30m | 362 |
| 64h:15m | 362 |
| 65h:05m | 369 |
| 65h:50m | 373 |
| 66h:35m | 376 |
| 67h:20m | 374 |
| 68h:10m | 375 |
| 68h:55m | 373 |
| 69h:40m | 374 |
| 70h:25m | 377 |
| 71h:15m | 378 |
| 72h:00m | 378 |

E.Coli Garamycin 1000MIC 1

| Time | EC 1000MIC GARA E.3 |
| --- | --- |
| 0h:05m | 600 |
| 0h:50m | 594 |
| 1h:40m | 599 |
| 2h:25m | 601 |
| 3h:10m | 604 |
| 3h:55m | 603 |
| 4h:45m | 606 |
| 5h:30m | 605 |
| 6h:15m | 604 |
| 7h:00m | 604 |
| 7h:50m | 607 |
| 8h:35m | 609 |
| 9h:20m | 606 |
| 10h:05m | 609 |
| 10h:55m | 608 |
| 11h:40m | 607 |
| 12h:25m | 610 |
| 13h:10m | 610 |
| 14h:00m | 609 |
| 14h:45m | 608 |
| 15h:30m | 611 |
| 16h:15m | 609 |
| 17h:05m | 613 |
| 17h:50m | 613 |
| 18h:35m | 615 |
| 19h:20m | 615 |
| 20h:10m | 614 |
| 20h:55m | 615 |
| 21h:40m | 615 |
| 22h:25m | 616 |
| 23h:15m | 619 |
| 24h:00m | 619 |
| 24h:05m | 601 |
| 24h:50m | 605 |
| 25h:40m | 605 |
| 26h:25m | 607 |
| 27h:10m | 606 |
| 27h:55m | 607 |
| 28h:45m | 607 |
| 29h:30m | 607 |
| 30h:15m | 607 |
| 31h:00m | 610 |
| 31h:50m | 609 |
| 32h:35m | 612 |
| 33h:20m | 612 |
| 34h:05m | 612 |
| 34h:55m | 611 |
| 35h:40m | 612 |
| 36h:25m | 612 |
| 37h:10m | 613 |
| 38h:00m | 616 |
| 38h:45m | 616 |
| 39h:30m | 617 |
| 40h:15m | 616 |
| 41h:05m | 616 |
| 41h:50m | 614 |
| 42h:35m | 616 |
| 43h:20m | 617 |
| 44h:10m | 618 |
| 44h:55m | 618 |
| 45h:40m | 617 |
| 46h:25m | 619 |
| 47h:15m | 618 |
| 48h:00m | 620 |
| 48h:05m | 689 |
| 48h:50m | 686 |
| 49h:40m | 689 |
| 50h:25m | 691 |
| 51h:10m | 688 |
| 51h:55m | 687 |
| 52h:45m | 689 |
| 53h:30m | 690 |
| 54h:15m | 689 |
| 55h:00m | 692 |
| 55h:50m | 693 |
| 56h:35m | 693 |
| 57h:20m | 698 |
| 58h:05m | 695 |
| 58h:55m | 697 |
| 59h:40m | 696 |
| 60h:25m | 699 |
| 61h:10m | 698 |
| 62h:00m | 699 |
| 62h:45m | 703 |
| 63h:30m | 699 |
| 64h:15m | 702 |
| 65h:05m | 698 |
| 65h:50m | 700 |
| 66h:35m | 701 |
| 67h:20m | 704 |
| 68h:10m | 697 |
| 68h:55m | 699 |
| 69h:40m | 701 |
| 70h:25m | 699 |
| 71h:15m | 702 |
| 72h:00m | 703 |

E.Coli Garamycin 1000MIC 2

| Time | EC 1000MIC GARA E.3 |
| --- | --- |
| 0h:05m | 951 |
| 0h:50m | 958 |
| 1h:40m | 971 |
| 2h:25m | 973 |
| 3h:10m | 971 |
| 3h:55m | 972 |
| 4h:45m | 976 |
| 5h:30m | 978 |
| 6h:15m | 979 |
| 7h:00m | 982 |
| 7h:50m | 980 |
| 8h:35m | 983 |
| 9h:20m | 981 |
| 10h:05m | 984 |
| 10h:55m | 986 |
| 11h:40m | 986 |
| 12h:25m | 986 |
| 13h:10m | 987 |
| 14h:00m | 987 |
| 14h:45m | 987 |
| 15h:30m | 988 |
| 16h:15m | 988 |
| 17h:05m | 985 |
| 17h:50m | 986 |
| 18h:35m | 985 |
| 19h:20m | 984 |
| 20h:10m | 986 |
| 20h:55m | 986 |
| 21h:40m | 987 |
| 22h:25m | 987 |
| 23h:15m | 990 |
| 24h:00m | 989 |
| 24h:05m | 623 |
| 24h:50m | 622 |
| 25h:40m | 625 |
| 26h:25m | 625 |
| 27h:10m | 624 |
| 27h:55m | 626 |
| 28h:45m | 627 |
| 29h:30m | 624 |
| 30h:15m | 624 |
| 31h:00m | 626 |
| 31h:50m | 624 |
| 32h:35m | 623 |
| 33h:20m | 624 |
| 34h:05m | 624 |
| 34h:55m | 624 |
| 35h:40m | 623 |
| 36h:25m | 623 |
| 37h:10m | 624 |
| 38h:00m | 624 |
| 38h:45m | 625 |
| 39h:30m | 625 |
| 40h:15m | 621 |
| 41h:05m | 622 |
| 41h:50m | 622 |
| 42h:35m | 624 |
| 43h:20m | 625 |
| 44h:10m | 622 |
| 44h:55m | 624 |
| 45h:40m | 623 |
| 46h:25m | 623 |
| 47h:15m | 623 |
| 48h:00m | 624 |
| 48h:05m | 664 |
| 48h:50m | 658 |
| 49h:40m | 657 |
| 50h:25m | 658 |
| 51h:10m | 656 |
| 51h:55m | 657 |
| 52h:45m | 655 |
| 53h:30m | 655 |
| 54h:15m | 656 |
| 55h:00m | 655 |
| 55h:50m | 654 |
| 56h:35m | 656 |
| 57h:20m | 655 |
| 58h:05m | 655 |
| 58h:55m | 655 |
| 59h:40m | 655 |
| 60h:25m | 653 |
| 61h:10m | 655 |
| 62h:00m | 653 |
| 62h:45m | 656 |
| 63h:30m | 654 |
| 64h:15m | 653 |
| 65h:05m | 654 |
| 65h:50m | 652 |
| 66h:35m | 657 |
| 67h:20m | 652 |
| 68h:10m | 653 |
| 68h:55m | 652 |
| 69h:40m | 651 |
| 70h:25m | 649 |
| 71h:15m | 650 |
| 72h:00m | 647 |

E.Coli Garamycin 1000MIC 3

| Time | EC 1000MIC GARA E.5 |
| --- | --- |
| 0h:05m | 491 |
| 0h:50m | 500 |
| 1h:40m | 500 |
| 2h:25m | 498 |
| 3h:10m | 499 |
| 3h:55m | 501 |
| 4h:45m | 500 |
| 5h:30m | 499 |
| 6h:15m | 499 |
| 7h:00m | 499 |
| 7h:50m | 498 |
| 8h:35m | 497 |
| 9h:20m | 498 |
| 10h:05m | 495 |
| 10h:55m | 495 |
| 11h:40m | 497 |
| 12h:25m | 495 |
| 13h:10m | 498 |
| 14h:00m | 494 |
| 14h:45m | 492 |
| 15h:30m | 496 |
| 16h:15m | 495 |
| 17h:05m | 497 |
| 17h:50m | 498 |
| 18h:35m | 498 |
| 19h:20m | 494 |
| 20h:10m | 502 |
| 20h:55m | 497 |
| 21h:40m | 497 |
| 22h:25m | 497 |
| 23h:15m | 498 |
| 24h:00m | 497 |
| 24h:05m |  |
| 24h:50m |  |
| 25h:40m |  |
| 26h:25m |  |
| 27h:10m |  |
| 27h:55m |  |
| 28h:45m |  |
| 29h:30m |  |
| 30h:15m |  |
| 31h:00m |  |
| 31h:50m |  |
| 32h:35m |  |
| 33h:20m |  |
| 34h:05m |  |
| 34h:55m |  |
| 35h:40m |  |
| 36h:25m |  |
| 37h:10m |  |
| 38h:00m |  |
| 38h:45m |  |
| 39h:30m |  |
| 40h:15m |  |
| 41h:05m |  |
| 41h:50m |  |
| 42h:35m |  |
| 43h:20m |  |
| 44h:10m |  |
| 44h:55m |  |
| 45h:40m |  |
| 46h:25m |  |
| 47h:15m |  |
| 48h:00m |  |
| 48h:05m | 494 |
| 48h:50m | 494 |
| 49h:40m | 496 |
| 50h:25m | 493 |
| 51h:10m | 493 |
| 51h:55m | 494 |
| 52h:45m | 494 |
| 53h:30m | 494 |
| 54h:15m | 496 |
| 55h:00m | 497 |
| 55h:50m | 497 |
| 56h:35m | 498 |
| 57h:20m | 497 |
| 58h:05m | 497 |
| 58h:55m | 500 |
| 59h:40m | 500 |
| 60h:25m | 500 |
| 61h:10m | 501 |
| 62h:00m | 502 |
| 62h:45m | 503 |
| 63h:30m | 502 |
| 64h:15m | 500 |
| 65h:05m | 502 |
| 65h:50m | 506 |
| 66h:35m | 506 |
| 67h:20m | 505 |
| 68h:10m | 506 |
| 68h:55m | 506 |
| 69h:40m | 506 |
| 70h:25m | 506 |
| 71h:15m | 511 |
| 72h:00m | 510 |

E.Coli Garamycin 1000MIC 4

| Time | EC 1000MIC GARA E.5 |
| --- | --- |
| 0h:05m | 556 |
| 0h:50m | 561 |
| 1h:40m | 562 |
| 2h:25m | 563 |
| 3h:10m | 567 |
| 3h:55m | 566 |
| 4h:45m | 565 |
| 5h:30m | 565 |
| 6h:15m | 564 |
| 7h:00m | 567 |
| 7h:50m | 565 |
| 8h:35m | 563 |
| 9h:20m | 563 |
| 10h:05m | 563 |
| 10h:55m | 563 |
| 11h:40m | 563 |
| 12h:25m | 562 |
| 13h:10m | 561 |
| 14h:00m | 561 |
| 14h:45m | 562 |
| 15h:30m | 560 |
| 16h:15m | 562 |
| 17h:05m | 563 |
| 17h:50m | 562 |
| 18h:35m | 561 |
| 19h:20m | 562 |
| 20h:10m | 563 |
| 20h:55m | 564 |
| 21h:40m | 562 |
| 22h:25m | 563 |
| 23h:15m | 563 |
| 24h:00m | 566 |
| 24h:05m |  |
| 24h:50m |  |
| 25h:40m |  |
| 26h:25m |  |
| 27h:10m |  |
| 27h:55m |  |
| 28h:45m |  |
| 29h:30m |  |
| 30h:15m |  |
| 31h:00m |  |
| 31h:50m |  |
| 32h:35m |  |
| 33h:20m |  |
| 34h:05m |  |
| 34h:55m |  |
| 35h:40m |  |
| 36h:25m |  |
| 37h:10m |  |
| 38h:00m |  |
| 38h:45m |  |
| 39h:30m |  |
| 40h:15m |  |
| 41h:05m |  |
| 41h:50m |  |
| 42h:35m |  |
| 43h:20m |  |
| 44h:10m |  |
| 44h:55m |  |
| 45h:40m |  |
| 46h:25m |  |
| 47h:15m |  |
| 48h:00m |  |
| 48h:05m | 588 |
| 48h:50m | 582 |
| 49h:40m | 584 |
| 50h:25m | 583 |
| 51h:10m | 584 |
| 51h:55m | 586 |
| 52h:45m | 585 |
| 53h:30m | 584 |
| 54h:15m | 586 |
| 55h:00m | 588 |
| 55h:50m | 586 |
| 56h:35m | 586 |
| 57h:20m | 590 |
| 58h:05m | 588 |
| 58h:55m | 589 |
| 59h:40m | 588 |
| 60h:25m | 589 |
| 61h:10m | 588 |
| 62h:00m | 592 |
| 62h:45m | 592 |
| 63h:30m | 589 |
| 64h:15m | 589 |
| 65h:05m | 593 |
| 65h:50m | 590 |
| 66h:35m | 594 |
| 67h:20m | 593 |
| 68h:10m | 596 |
| 68h:55m | 594 |
| 69h:40m | 595 |
| 70h:25m | 598 |
| 71h:15m | 597 |
| 72h:00m | 594 |

E.Coli Garamycin 1000MIC 5

| Time | EC 1000MIC GARA E.4 |
| --- | --- |
| 0h:05m | 913 |
| 0h:50m | 903 |
| 1h:40m | 901 |
| 2h:25m | 903 |
| 3h:10m | 901 |
| 3h:55m | 901 |
| 4h:45m | 899 |
| 5h:30m | 899 |
| 6h:15m | 901 |
| 7h:00m | 906 |
| 7h:50m | 903 |
| 8h:35m | 902 |
| 9h:20m | 903 |
| 10h:05m | 907 |
| 10h:55m | 905 |
| 11h:40m | 906 |
| 12h:25m | 906 |
| 13h:10m | 910 |
| 14h:00m | 908 |
| 14h:45m | 908 |
| 15h:30m | 914 |
| 16h:15m | 911 |
| 17h:05m | 912 |
| 17h:50m | 913 |
| 18h:35m | 912 |
| 19h:20m | 914 |
| 20h:10m | 915 |
| 20h:55m | 918 |
| 21h:40m | 919 |
| 22h:25m | 920 |
| 23h:15m | 918 |
| 24h:00m | 914 |
| 24h:05m | 1239 |
| 24h:50m | 1234 |
| 25h:40m | 1231 |
| 26h:25m | 1234 |
| 27h:10m | 1234 |
| 27h:55m | 1235 |
| 28h:45m | 1235 |
| 29h:30m | 1231 |
| 30h:15m | 1235 |
| 31h:00m | 1228 |
| 31h:50m | 1230 |
| 32h:35m | 1228 |
| 33h:20m | 1227 |
| 34h:05m | 1226 |
| 34h:55m | 1231 |
| 35h:40m | 1227 |
| 36h:25m | 1228 |
| 37h:10m | 1233 |
| 38h:00m | 1232 |
| 38h:45m | 1230 |
| 39h:30m | 1238 |
| 40h:15m | 1235 |
| 41h:05m | 1238 |
| 41h:50m | 1235 |
| 42h:35m | 1234 |
| 43h:20m | 1232 |
| 44h:10m | 1237 |
| 44h:55m | 1232 |
| 45h:40m | 1237 |
| 46h:25m | 1232 |
| 47h:15m | 1236 |
| 48h:00m | 1241 |
| 48h:05m | 733 |
| 48h:50m | 735 |
| 49h:40m | 730 |
| 50h:25m | 729 |
| 51h:10m | 735 |
| 51h:55m | 733 |
| 52h:45m | 734 |
| 53h:30m | 734 |
| 54h:15m | 732 |
| 55h:00m | 735 |
| 55h:50m | 736 |
| 56h:35m | 738 |
| 57h:20m | 738 |
| 58h:05m | 737 |
| 58h:55m | 736 |
| 59h:40m | 742 |
| 60h:25m | 738 |
| 61h:10m | 739 |
| 62h:00m | 737 |
| 62h:45m | 740 |
| 63h:30m | 744 |
| 64h:15m | 737 |
| 65h:05m | 738 |
| 65h:50m | 738 |
| 66h:35m | 741 |
| 67h:20m | 742 |
| 68h:10m | 743 |
| 68h:55m | 738 |
| 69h:40m | 744 |
| 70h:25m | 744 |
| 71h:15m | 738 |
| 72h:00m | 758 |

E.Coli Garamycin 1000MIC 6

| Time | EC 1000MIC GARA E.4 |
| --- | --- |
| 0h:05m | 577 |
| 0h:50m | 572 |
| 1h:40m | 578 |
| 2h:25m | 576 |
| 3h:10m | 580 |
| 3h:55m | 581 |
| 4h:45m | 580 |
| 5h:30m | 581 |
| 6h:15m | 582 |
| 7h:00m | 580 |
| 7h:50m | 583 |
| 8h:35m | 585 |
| 9h:20m | 585 |
| 10h:05m | 587 |
| 10h:55m | 589 |
| 11h:40m | 588 |
| 12h:25m | 590 |
| 13h:10m | 589 |
| 14h:00m | 592 |
| 14h:45m | 595 |
| 15h:30m | 594 |
| 16h:15m | 597 |
| 17h:05m | 595 |
| 17h:50m | 593 |
| 18h:35m | 597 |
| 19h:20m | 598 |
| 20h:10m | 599 |
| 20h:55m | 599 |
| 21h:40m | 597 |
| 22h:25m | 600 |
| 23h:15m | 601 |
| 24h:00m | 598 |
| 24h:05m | 736 |
| 24h:50m | 731 |
| 25h:40m | 727 |
| 26h:25m | 728 |
| 27h:10m | 725 |
| 27h:55m | 723 |
| 28h:45m | 731 |
| 29h:30m | 728 |
| 30h:15m | 726 |
| 31h:00m | 726 |
| 31h:50m | 728 |
| 32h:35m | 730 |
| 33h:20m | 726 |
| 34h:05m | 728 |
| 34h:55m | 729 |
| 35h:40m | 726 |
| 36h:25m | 729 |
| 37h:10m | 729 |
| 38h:00m | 729 |
| 38h:45m | 728 |
| 39h:30m | 728 |
| 40h:15m | 731 |
| 41h:05m | 729 |
| 41h:50m | 729 |
| 42h:35m | 725 |
| 43h:20m | 732 |
| 44h:10m | 731 |
| 44h:55m | 731 |
| 45h:40m | 730 |
| 46h:25m | 733 |
| 47h:15m | 732 |
| 48h:00m | 728 |
| 48h:05m | 757 |
| 48h:50m | 762 |
| 49h:40m | 760 |
| 50h:25m | 762 |
| 51h:10m | 759 |
| 51h:55m | 763 |
| 52h:45m | 761 |
| 53h:30m | 762 |
| 54h:15m | 763 |
| 55h:00m | 764 |
| 55h:50m | 763 |
| 56h:35m | 765 |
| 57h:20m | 764 |
| 58h:05m | 764 |
| 58h:55m | 767 |
| 59h:40m | 767 |
| 60h:25m | 766 |
| 61h:10m | 767 |
| 62h:00m | 767 |
| 62h:45m | 765 |
| 63h:30m | 767 |
| 64h:15m | 766 |
| 65h:05m | 767 |
| 65h:50m | 767 |
| 66h:35m | 767 |
| 67h:20m | 768 |
| 68h:10m | 767 |
| 68h:55m | 769 |
| 69h:40m | 768 |
| 70h:25m | 768 |
| 71h:15m | 769 |
| 72h:00m | 770 |

E.Coli Garamycin 1000MIC 7

| Time | EC 1000MIC GARA E.W |
| --- | --- |
| 0h:05m | 393 |
| 0h:50m | 407 |
| 1h:40m | 420 |
| 2h:25m | 427 |
| 3h:10m | 430 |
| 3h:55m | 432 |
| 4h:45m | 434 |
| 5h:30m | 437 |
| 6h:15m | 440 |
| 7h:00m | 445 |
| 7h:50m | 443 |
| 8h:35m | 446 |
| 9h:20m | 449 |
| 10h:05m | 448 |
| 10h:55m | 448 |
| 11h:40m | 450 |
| 12h:25m | 450 |
| 13h:10m | 452 |
| 14h:00m | 451 |
| 14h:45m | 453 |
| 15h:30m | 455 |
| 16h:15m | 454 |
| 17h:05m | 456 |
| 17h:50m | 458 |
| 18h:35m | 456 |
| 19h:20m | 457 |
| 20h:10m | 458 |
| 20h:55m | 458 |
| 21h:40m | 460 |
| 22h:25m | 458 |
| 23h:15m | 456 |
| 24h:00m | 460 |
| 24h:05m | 483 |
| 24h:50m | 485 |
| 25h:40m | 485 |
| 26h:25m | 488 |
| 27h:10m | 488 |
| 27h:55m | 487 |
| 28h:45m | 487 |
| 29h:30m | 486 |
| 30h:15m | 487 |
| 31h:00m | 489 |
| 31h:50m | 485 |
| 32h:35m | 486 |
| 33h:20m | 483 |
| 34h:05m | 485 |
| 34h:55m | 484 |
| 35h:40m | 487 |
| 36h:25m | 487 |
| 37h:10m | 489 |
| 38h:00m | 494 |
| 38h:45m | 495 |
| 39h:30m | 497 |
| 40h:15m | 499 |
| 41h:05m | 505 |
| 41h:50m | 509 |
| 42h:35m | 510 |
| 43h:20m | 511 |
| 44h:10m | 514 |
| 44h:55m | 520 |
| 45h:40m | 517 |
| 46h:25m | 518 |
| 47h:15m | 522 |
| 48h:00m | 527 |
| 48h:05m | 687 |
| 48h:50m | 719 |
| 49h:40m | 743 |
| 50h:25m | 750 |
| 51h:10m | 759 |
| 51h:55m | 772 |
| 52h:45m | 778 |
| 53h:30m | 780 |
| 54h:15m | 781 |
| 55h:00m | 783 |
| 55h:50m | 782 |
| 56h:35m | 783 |
| 57h:20m | 784 |
| 58h:05m | 780 |
| 58h:55m | 779 |
| 59h:40m | 782 |
| 60h:25m | 780 |
| 61h:10m | 777 |
| 62h:00m | 778 |
| 62h:45m | 776 |
| 63h:30m | 777 |
| 64h:15m | 776 |
| 65h:05m | 776 |
| 65h:50m | 772 |
| 66h:35m | 770 |
| 67h:20m | 768 |
| 68h:10m | 770 |
| 68h:55m | 765 |
| 69h:40m | 765 |
| 70h:25m | 762 |
| 71h:15m | 763 |
| 72h:00m | 779 |

E.Coli Garamycin 1000MIC 8

| Time | EC 1000MIC GARA E.W |
| --- | --- |
| 0h:05m | 359 |
| 0h:50m | 375 |
| 1h:40m | 381 |
| 2h:25m | 388 |
| 3h:10m | 394 |
| 3h:55m | 399 |
| 4h:45m | 401 |
| 5h:30m | 404 |
| 6h:15m | 406 |
| 7h:00m | 409 |
| 7h:50m | 410 |
| 8h:35m | 415 |
| 9h:20m | 413 |
| 10h:05m | 415 |
| 10h:55m | 414 |
| 11h:40m | 416 |
| 12h:25m | 417 |
| 13h:10m | 417 |
| 14h:00m | 418 |
| 14h:45m | 417 |
| 15h:30m | 419 |
| 16h:15m | 421 |
| 17h:05m | 421 |
| 17h:50m | 421 |
| 18h:35m | 422 |
| 19h:20m | 423 |
| 20h:10m | 422 |
| 20h:55m | 425 |
| 21h:40m | 424 |
| 22h:25m | 424 |
| 23h:15m | 430 |
| 24h:00m | 425 |
| 24h:05m | 497 |
| 24h:50m | 498 |
| 25h:40m | 496 |
| 26h:25m | 500 |
| 27h:10m | 501 |
| 27h:55m | 496 |
| 28h:45m | 502 |
| 29h:30m | 499 |
| 30h:15m | 501 |
| 31h:00m | 501 |
| 31h:50m | 500 |
| 32h:35m | 502 |
| 33h:20m | 501 |
| 34h:05m | 502 |
| 34h:55m | 502 |
| 35h:40m | 503 |
| 36h:25m | 501 |
| 37h:10m | 501 |
| 38h:00m | 502 |
| 38h:45m | 502 |
| 39h:30m | 501 |
| 40h:15m | 503 |
| 41h:05m | 501 |
| 41h:50m | 503 |
| 42h:35m | 503 |
| 43h:20m | 502 |
| 44h:10m | 502 |
| 44h:55m | 501 |
| 45h:40m | 503 |
| 46h:25m | 504 |
| 47h:15m | 502 |
| 48h:00m | 506 |
| 48h:05m | 460 |
| 48h:50m | 460 |
| 49h:40m | 462 |
| 50h:25m | 461 |
| 51h:10m | 459 |
| 51h:55m | 458 |
| 52h:45m | 460 |
| 53h:30m | 458 |
| 54h:15m | 456 |
| 55h:00m | 460 |
| 55h:50m | 458 |
| 56h:35m | 460 |
| 57h:20m | 458 |
| 58h:05m | 457 |
| 58h:55m | 459 |
| 59h:40m | 459 |
| 60h:25m | 458 |
| 61h:10m | 458 |
| 62h:00m | 457 |
| 62h:45m | 458 |
| 63h:30m | 458 |
| 64h:15m | 458 |
| 65h:05m | 455 |
| 65h:50m | 454 |
| 66h:35m | 456 |
| 67h:20m | 455 |
| 68h:10m | 453 |
| 68h:55m | 453 |
| 69h:40m | 457 |
| 70h:25m | 456 |
| 71h:15m | 454 |
| 72h:00m | 453 |

E.Coli Amikacin control 1

| Time | EC Cont E.3 |
| --- | --- |
| 0h:05m | 1128 |
| 0h:50m | 1156 |
| 1h:40m | 1344 |
| 2h:25m | 2884 |
| 3h:10m | 17318 |
| 3h:55m | 30964 |
| 4h:45m | 30934 |
| 5h:30m | 30927 |
| 6h:15m | 30926 |
| 7h:00m | 30924 |
| 7h:50m | 30925 |
| 8h:35m | 30924 |
| 9h:20m | 30925 |
| 10h:05m | 30926 |
| 10h:55m | 30926 |
| 11h:40m | 30925 |
| 12h:25m | 30926 |
| 13h:10m | 30926 |
| 14h:00m | 30926 |
| 14h:45m | 30926 |
| 15h:30m | 30926 |
| 16h:15m | 30927 |
| 17h:05m | 30926 |
| 17h:50m | 30927 |
| 18h:35m | 30926 |
| 19h:20m | 30927 |
| 20h:10m | 30929 |
| 20h:55m | 30927 |
| 21h:40m | 30927 |
| 22h:25m | 30926 |
| 23h:15m | 30925 |
| 24h:00m | 30925 |
| 24h:05m |  |
| 24h:50m |  |
| 25h:40m |  |
| 26h:25m |  |
| 27h:10m |  |
| 27h:55m |  |
| 28h:45m |  |
| 29h:30m |  |
| 30h:15m |  |
| 31h:00m |  |
| 31h:50m |  |
| 32h:35m |  |
| 33h:20m |  |
| 34h:05m |  |
| 34h:55m |  |
| 35h:40m |  |
| 36h:25m |  |
| 37h:10m |  |
| 38h:00m |  |
| 38h:45m |  |
| 39h:30m |  |
| 40h:15m |  |
| 41h:05m |  |
| 41h:50m |  |
| 42h:35m |  |
| 43h:20m |  |
| 44h:10m |  |
| 44h:55m |  |
| 45h:40m |  |
| 46h:25m |  |
| 47h:15m |  |
| 48h:00m |  |
| 48h:05m |  |
| 48h:50m |  |
| 49h:40m |  |
| 50h:25m |  |
| 51h:10m |  |
| 51h:55m |  |
| 52h:45m |  |
| 53h:30m |  |
| 54h:15m |  |
| 55h:00m |  |
| 55h:50m |  |
| 56h:35m |  |
| 57h:20m |  |
| 58h:05m |  |
| 58h:55m |  |
| 59h:40m |  |
| 60h:25m |  |
| 61h:10m |  |
| 62h:00m |  |
| 62h:45m |  |
| 63h:30m |  |
| 64h:15m |  |
| 65h:05m |  |
| 65h:50m |  |
| 66h:35m |  |
| 67h:20m |  |
| 68h:10m |  |
| 68h:55m |  |
| 69h:40m |  |
| 70h:25m |  |
| 71h:15m |  |
| 72h:00m |  |

E.Coli Amikacin control 2

| Time | EC Cont E.5 |
| --- | --- |
| 0h:05m | 609 |
| 0h:50m | 622 |
| 1h:40m | 800 |
| 2h:25m | 2209 |
| 3h:10m | 13611 |
| 3h:55m | 30968 |
| 4h:45m | 30933 |
| 5h:30m | 30925 |
| 6h:15m | 30925 |
| 7h:00m | 30927 |
| 7h:50m | 30927 |
| 8h:35m | 30927 |
| 9h:20m | 30926 |
| 10h:05m | 30925 |
| 10h:55m | 30925 |
| 11h:40m | 30925 |
| 12h:25m | 30925 |
| 13h:10m | 30926 |
| 14h:00m | 30925 |
| 14h:45m | 30925 |
| 15h:30m | 30925 |
| 16h:15m | 30926 |
| 17h:05m | 30925 |
| 17h:50m | 30925 |
| 18h:35m | 30925 |
| 19h:20m | 30925 |
| 20h:10m | 30927 |
| 20h:55m | 30926 |
| 21h:40m | 30926 |
| 22h:25m | 30926 |
| 23h:15m | 30926 |
| 24h:00m | 30925 |
| 24h:05m |  |
| 24h:50m |  |
| 25h:40m |  |
| 26h:25m |  |
| 27h:10m |  |
| 27h:55m |  |
| 28h:45m |  |
| 29h:30m |  |
| 30h:15m |  |
| 31h:00m |  |
| 31h:50m |  |
| 32h:35m |  |
| 33h:20m |  |
| 34h:05m |  |
| 34h:55m |  |
| 35h:40m |  |
| 36h:25m |  |
| 37h:10m |  |
| 38h:00m |  |
| 38h:45m |  |
| 39h:30m |  |
| 40h:15m |  |
| 41h:05m |  |
| 41h:50m |  |
| 42h:35m |  |
| 43h:20m |  |
| 44h:10m |  |
| 44h:55m |  |
| 45h:40m |  |
| 46h:25m |  |
| 47h:15m |  |
| 48h:00m |  |
| 48h:05m |  |
| 48h:50m |  |
| 49h:40m |  |
| 50h:25m |  |
| 51h:10m |  |
| 51h:55m |  |
| 52h:45m |  |
| 53h:30m |  |
| 54h:15m |  |
| 55h:00m |  |
| 55h:50m |  |
| 56h:35m |  |
| 57h:20m |  |
| 58h:05m |  |
| 58h:55m |  |
| 59h:40m |  |
| 60h:25m |  |
| 61h:10m |  |
| 62h:00m |  |
| 62h:45m |  |
| 63h:30m |  |
| 64h:15m |  |
| 65h:05m |  |
| 65h:50m |  |
| 66h:35m |  |
| 67h:20m |  |
| 68h:10m |  |
| 68h:55m |  |
| 69h:40m |  |
| 70h:25m |  |
| 71h:15m |  |
| 72h:00m |  |

E.Coli Amikacin control 3

| Time | EC Cont E.4 |
| --- | --- |
| 0h:05m | 860 |
| 0h:50m | 843 |
| 1h:40m | 960 |
| 2h:25m | 1686 |
| 3h:10m | 5806 |
| 3h:55m | 18986 |
| 4h:45m | 30967 |
| 5h:30m | 30936 |
| 6h:15m | 30929 |
| 7h:00m | 30928 |
| 7h:50m | 30928 |
| 8h:35m | 30928 |
| 9h:20m | 30928 |
| 10h:05m | 30929 |
| 10h:55m | 30929 |
| 11h:40m | 30929 |
| 12h:25m | 30928 |
| 13h:10m | 30930 |
| 14h:00m | 30930 |
| 14h:45m | 30930 |
| 15h:30m | 30930 |
| 16h:15m | 30930 |
| 17h:05m | 30930 |
| 17h:50m | 30930 |
| 18h:35m | 30931 |
| 19h:20m | 30929 |
| 20h:10m | 30928 |
| 20h:55m | 30926 |
| 21h:40m | 30926 |
| 22h:25m | 30927 |
| 23h:15m | 30927 |
| 24h:00m | 30926 |
| 24h:05m |  |
| 24h:50m |  |
| 25h:40m |  |
| 26h:25m |  |
| 27h:10m |  |
| 27h:55m |  |
| 28h:45m |  |
| 29h:30m |  |
| 30h:15m |  |
| 31h:00m |  |
| 31h:50m |  |
| 32h:35m |  |
| 33h:20m |  |
| 34h:05m |  |
| 34h:55m |  |
| 35h:40m |  |
| 36h:25m |  |
| 37h:10m |  |
| 38h:00m |  |
| 38h:45m |  |
| 39h:30m |  |
| 40h:15m |  |
| 41h:05m |  |
| 41h:50m |  |
| 42h:35m |  |
| 43h:20m |  |
| 44h:10m |  |
| 44h:55m |  |
| 45h:40m |  |
| 46h:25m |  |
| 47h:15m |  |
| 48h:00m |  |
| 48h:05m |  |
| 48h:50m |  |
| 49h:40m |  |
| 50h:25m |  |
| 51h:10m |  |
| 51h:55m |  |
| 52h:45m |  |
| 53h:30m |  |
| 54h:15m |  |
| 55h:00m |  |
| 55h:50m |  |
| 56h:35m |  |
| 57h:20m |  |
| 58h:05m |  |
| 58h:55m |  |
| 59h:40m |  |
| 60h:25m |  |
| 61h:10m |  |
| 62h:00m |  |
| 62h:45m |  |
| 63h:30m |  |
| 64h:15m |  |
| 65h:05m |  |
| 65h:50m |  |
| 66h:35m |  |
| 67h:20m |  |
| 68h:10m |  |
| 68h:55m |  |
| 69h:40m |  |
| 70h:25m |  |
| 71h:15m |  |
| 72h:00m |  |

E.Coli Amikacin 1MIC 1

| Time | EC 1MIC AMIK E.3 |
| --- | --- |
| 0h:05m | 630 |
| 0h:50m | 640 |
| 1h:40m | 645 |
| 2h:25m | 647 |
| 3h:10m | 644 |
| 3h:55m | 644 |
| 4h:45m | 644 |
| 5h:30m | 643 |
| 6h:15m | 643 |
| 7h:00m | 643 |
| 7h:50m | 642 |
| 8h:35m | 648 |
| 9h:20m | 653 |
| 10h:05m | 667 |
| 10h:55m | 710 |
| 11h:40m | 843 |
| 12h:25m | 1234 |
| 13h:10m | 2460 |
| 14h:00m | 8012 |
| 14h:45m | 28968 |
| 15h:30m | 30941 |
| 16h:15m | 30926 |
| 17h:05m | 30921 |
| 17h:50m | 30919 |
| 18h:35m | 30918 |
| 19h:20m | 30920 |
| 20h:10m | 30921 |
| 20h:55m | 30922 |
| 21h:40m | 30921 |
| 22h:25m | 30920 |
| 23h:15m | 30918 |
| 24h:00m | 30919 |
| 24h:05m |  |
| 24h:50m |  |
| 25h:40m |  |
| 26h:25m |  |
| 27h:10m |  |
| 27h:55m |  |
| 28h:45m |  |
| 29h:30m |  |
| 30h:15m |  |
| 31h:00m |  |
| 31h:50m |  |
| 32h:35m |  |
| 33h:20m |  |
| 34h:05m |  |
| 34h:55m |  |
| 35h:40m |  |
| 36h:25m |  |
| 37h:10m |  |
| 38h:00m |  |
| 38h:45m |  |
| 39h:30m |  |
| 40h:15m |  |
| 41h:05m |  |
| 41h:50m |  |
| 42h:35m |  |
| 43h:20m |  |
| 44h:10m |  |
| 44h:55m |  |
| 45h:40m |  |
| 46h:25m |  |
| 47h:15m |  |
| 48h:00m |  |
| 48h:05m |  |
| 48h:50m |  |
| 49h:40m |  |
| 50h:25m |  |
| 51h:10m |  |
| 51h:55m |  |
| 52h:45m |  |
| 53h:30m |  |
| 54h:15m |  |
| 55h:00m |  |
| 55h:50m |  |
| 56h:35m |  |
| 57h:20m |  |
| 58h:05m |  |
| 58h:55m |  |
| 59h:40m |  |
| 60h:25m |  |
| 61h:10m |  |
| 62h:00m |  |
| 62h:45m |  |
| 63h:30m |  |
| 64h:15m |  |
| 65h:05m |  |
| 65h:50m |  |
| 66h:35m |  |
| 67h:20m |  |
| 68h:10m |  |
| 68h:55m |  |
| 69h:40m |  |
| 70h:25m |  |
| 71h:15m |  |
| 72h:00m |  |

E.Coli Amikacin 1MIC 2

| Time | EC 1MIC AMIK E.3 |
| --- | --- |
| 0h:05m | 627 |
| 0h:50m | 625 |
| 1h:40m | 621 |
| 2h:25m | 619 |
| 3h:10m | 614 |
| 3h:55m | 611 |
| 4h:45m | 609 |
| 5h:30m | 609 |
| 6h:15m | 608 |
| 7h:00m | 608 |
| 7h:50m | 608 |
| 8h:35m | 609 |
| 9h:20m | 611 |
| 10h:05m | 611 |
| 10h:55m | 617 |
| 11h:40m | 636 |
| 12h:25m | 680 |
| 13h:10m | 802 |
| 14h:00m | 1170 |
| 14h:45m | 2098 |
| 15h:30m | 4578 |
| 16h:15m | 12321 |
| 17h:05m | 30985 |
| 17h:50m | 30932 |
| 18h:35m | 30925 |
| 19h:20m | 30921 |
| 20h:10m | 30920 |
| 20h:55m | 30919 |
| 21h:40m | 30920 |
| 22h:25m | 30918 |
| 23h:15m | 30918 |
| 24h:00m | 30918 |
| 24h:05m |  |
| 24h:50m |  |
| 25h:40m |  |
| 26h:25m |  |
| 27h:10m |  |
| 27h:55m |  |
| 28h:45m |  |
| 29h:30m |  |
| 30h:15m |  |
| 31h:00m |  |
| 31h:50m |  |
| 32h:35m |  |
| 33h:20m |  |
| 34h:05m |  |
| 34h:55m |  |
| 35h:40m |  |
| 36h:25m |  |
| 37h:10m |  |
| 38h:00m |  |
| 38h:45m |  |
| 39h:30m |  |
| 40h:15m |  |
| 41h:05m |  |
| 41h:50m |  |
| 42h:35m |  |
| 43h:20m |  |
| 44h:10m |  |
| 44h:55m |  |
| 45h:40m |  |
| 46h:25m |  |
| 47h:15m |  |
| 48h:00m |  |
| 48h:05m |  |
| 48h:50m |  |
| 49h:40m |  |
| 50h:25m |  |
| 51h:10m |  |
| 51h:55m |  |
| 52h:45m |  |
| 53h:30m |  |
| 54h:15m |  |
| 55h:00m |  |
| 55h:50m |  |
| 56h:35m |  |
| 57h:20m |  |
| 58h:05m |  |
| 58h:55m |  |
| 59h:40m |  |
| 60h:25m |  |
| 61h:10m |  |
| 62h:00m |  |
| 62h:45m |  |
| 63h:30m |  |
| 64h:15m |  |
| 65h:05m |  |
| 65h:50m |  |
| 66h:35m |  |
| 67h:20m |  |
| 68h:10m |  |
| 68h:55m |  |
| 69h:40m |  |
| 70h:25m |  |
| 71h:15m |  |
| 72h:00m |  |

E.Coli Amikacin 1MIC 3

| Time | EC 1MIC AMIK E.5 |
| --- | --- |
| 0h:05m | 598 |
| 0h:50m | 592 |
| 1h:40m | 592 |
| 2h:25m | 592 |
| 3h:10m | 590 |
| 3h:55m | 589 |
| 4h:45m | 586 |
| 5h:30m | 587 |
| 6h:15m | 586 |
| 7h:00m | 587 |
| 7h:50m | 587 |
| 8h:35m | 591 |
| 9h:20m | 600 |
| 10h:05m | 638 |
| 10h:55m | 765 |
| 11h:40m | 1183 |
| 12h:25m | 2637 |
| 13h:10m | 8259 |
| 14h:00m | 30987 |
| 14h:45m | 30955 |
| 15h:30m | 30941 |
| 16h:15m | 30932 |
| 17h:05m | 30929 |
| 17h:50m | 30930 |
| 18h:35m | 30931 |
| 19h:20m | 30932 |
| 20h:10m | 30934 |
| 20h:55m | 30937 |
| 21h:40m | 30938 |
| 22h:25m | 30938 |
| 23h:15m | 30939 |
| 24h:00m | 30939 |
| 24h:05m |  |
| 24h:50m |  |
| 25h:40m |  |
| 26h:25m |  |
| 27h:10m |  |
| 27h:55m |  |
| 28h:45m |  |
| 29h:30m |  |
| 30h:15m |  |
| 31h:00m |  |
| 31h:50m |  |
| 32h:35m |  |
| 33h:20m |  |
| 34h:05m |  |
| 34h:55m |  |
| 35h:40m |  |
| 36h:25m |  |
| 37h:10m |  |
| 38h:00m |  |
| 38h:45m |  |
| 39h:30m |  |
| 40h:15m |  |
| 41h:05m |  |
| 41h:50m |  |
| 42h:35m |  |
| 43h:20m |  |
| 44h:10m |  |
| 44h:55m |  |
| 45h:40m |  |
| 46h:25m |  |
| 47h:15m |  |
| 48h:00m |  |
| 48h:05m |  |
| 48h:50m |  |
| 49h:40m |  |
| 50h:25m |  |
| 51h:10m |  |
| 51h:55m |  |
| 52h:45m |  |
| 53h:30m |  |
| 54h:15m |  |
| 55h:00m |  |
| 55h:50m |  |
| 56h:35m |  |
| 57h:20m |  |
| 58h:05m |  |
| 58h:55m |  |
| 59h:40m |  |
| 60h:25m |  |
| 61h:10m |  |
| 62h:00m |  |
| 62h:45m |  |
| 63h:30m |  |
| 64h:15m |  |
| 65h:05m |  |
| 65h:50m |  |
| 66h:35m |  |
| 67h:20m |  |
| 68h:10m |  |
| 68h:55m |  |
| 69h:40m |  |
| 70h:25m |  |
| 71h:15m |  |
| 72h:00m |  |

E.Coli Amikacin 1MIC 4

| Time | EC 1MIC AMIK E.4 |
| --- | --- |
| 0h:05m | 900 |
| 0h:50m | 878 |
| 1h:40m | 880 |
| 2h:25m | 876 |
| 3h:10m | 874 |
| 3h:55m | 874 |
| 4h:45m | 873 |
| 5h:30m | 874 |
| 6h:15m | 876 |
| 7h:00m | 882 |
| 7h:50m | 907 |
| 8h:35m | 996 |
| 9h:20m | 1459 |
| 10h:05m | 3590 |
| 10h:55m | 13105 |
| 11h:40m | 30903 |
| 12h:25m | 30969 |
| 13h:10m | 30941 |
| 14h:00m | 30931 |
| 14h:45m | 30929 |
| 15h:30m | 30928 |
| 16h:15m | 30927 |
| 17h:05m | 30926 |
| 17h:50m | 30928 |
| 18h:35m | 30928 |
| 19h:20m | 30927 |
| 20h:10m | 30926 |
| 20h:55m | 30926 |
| 21h:40m | 30925 |
| 22h:25m | 30926 |
| 23h:15m | 30928 |
| 24h:00m | 30926 |
| 24h:05m |  |
| 24h:50m |  |
| 25h:40m |  |
| 26h:25m |  |
| 27h:10m |  |
| 27h:55m |  |
| 28h:45m |  |
| 29h:30m |  |
| 30h:15m |  |
| 31h:00m |  |
| 31h:50m |  |
| 32h:35m |  |
| 33h:20m |  |
| 34h:05m |  |
| 34h:55m |  |
| 35h:40m |  |
| 36h:25m |  |
| 37h:10m |  |
| 38h:00m |  |
| 38h:45m |  |
| 39h:30m |  |
| 40h:15m |  |
| 41h:05m |  |
| 41h:50m |  |
| 42h:35m |  |
| 43h:20m |  |
| 44h:10m |  |
| 44h:55m |  |
| 45h:40m |  |
| 46h:25m |  |
| 47h:15m |  |
| 48h:00m |  |
| 48h:05m |  |
| 48h:50m |  |
| 49h:40m |  |
| 50h:25m |  |
| 51h:10m |  |
| 51h:55m |  |
| 52h:45m |  |
| 53h:30m |  |
| 54h:15m |  |
| 55h:00m |  |
| 55h:50m |  |
| 56h:35m |  |
| 57h:20m |  |
| 58h:05m |  |
| 58h:55m |  |
| 59h:40m |  |
| 60h:25m |  |
| 61h:10m |  |
| 62h:00m |  |
| 62h:45m |  |
| 63h:30m |  |
| 64h:15m |  |
| 65h:05m |  |
| 65h:50m |  |
| 66h:35m |  |
| 67h:20m |  |
| 68h:10m |  |
| 68h:55m |  |
| 69h:40m |  |
| 70h:25m |  |
| 71h:15m |  |
| 72h:00m |  |

E.Coli Amikacin 1MIC 5

| Time | EC 1MIC AMIK E.4 |
| --- | --- |
| 0h:05m | 829 |
| 0h:50m | 807 |
| 1h:40m | 803 |
| 2h:25m | 801 |
| 3h:10m | 803 |
| 3h:55m | 803 |
| 4h:45m | 800 |
| 5h:30m | 801 |
| 6h:15m | 800 |
| 7h:00m | 804 |
| 7h:50m | 808 |
| 8h:35m | 818 |
| 9h:20m | 854 |
| 10h:05m | 1057 |
| 10h:55m | 1809 |
| 11h:40m | 4466 |
| 12h:25m | 14591 |
| 13h:10m | 30977 |
| 14h:00m | 30931 |
| 14h:45m | 30925 |
| 15h:30m | 30924 |
| 16h:15m | 30923 |
| 17h:05m | 30923 |
| 17h:50m | 30924 |
| 18h:35m | 30924 |
| 19h:20m |  |
| 20h:10m |  |
| 20h:55m |  |
| 21h:40m |  |
| 22h:25m |  |
| 23h:15m |  |
| 24h:00m |  |
| 24h:05m |  |
| 24h:50m |  |
| 25h:40m |  |
| 26h:25m |  |
| 27h:10m |  |
| 27h:55m |  |
| 28h:45m |  |
| 29h:30m |  |
| 30h:15m |  |
| 31h:00m |  |
| 31h:50m |  |
| 32h:35m |  |
| 33h:20m |  |
| 34h:05m |  |
| 34h:55m |  |
| 35h:40m |  |
| 36h:25m |  |
| 37h:10m |  |
| 38h:00m |  |
| 38h:45m |  |
| 39h:30m |  |
| 40h:15m |  |
| 41h:05m |  |
| 41h:50m |  |
| 42h:35m |  |
| 43h:20m |  |
| 44h:10m |  |
| 44h:55m |  |
| 45h:40m |  |
| 46h:25m |  |
| 47h:15m |  |
| 48h:00m |  |
| 48h:05m |  |
| 48h:50m |  |
| 49h:40m |  |
| 50h:25m |  |
| 51h:10m |  |
| 51h:55m |  |
| 52h:45m |  |
| 53h:30m |  |
| 54h:15m |  |
| 55h:00m |  |
| 55h:50m |  |
| 56h:35m |  |
| 57h:20m |  |
| 58h:05m |  |
| 58h:55m |  |
| 59h:40m |  |
| 60h:25m |  |
| 61h:10m |  |
| 62h:00m |  |
| 62h:45m |  |
| 63h:30m |  |
| 64h:15m |  |
| 65h:05m |  |
| 65h:50m |  |
| 66h:35m |  |
| 67h:20m |  |
| 68h:10m |  |
| 68h:55m |  |
| 69h:40m |  |
| 70h:25m |  |
| 71h:15m |  |
| 72h:00m |  |

E.Coli Amikacin 4MIC 1

| Time | EC 4MIC AMIK E.3 |
| --- | --- |
| 0h:05m | 689 |
| 0h:50m | 675 |
| 1h:40m | 667 |
| 2h:25m | 663 |
| 3h:10m | 659 |
| 3h:55m | 657 |
| 4h:45m | 657 |
| 5h:30m | 657 |
| 6h:15m | 655 |
| 7h:00m | 654 |
| 7h:50m | 652 |
| 8h:35m | 652 |
| 9h:20m | 654 |
| 10h:05m | 654 |
| 10h:55m | 653 |
| 11h:40m | 654 |
| 12h:25m | 652 |
| 13h:10m | 654 |
| 14h:00m | 654 |
| 14h:45m | 653 |
| 15h:30m | 653 |
| 16h:15m | 651 |
| 17h:05m | 655 |
| 17h:50m | 653 |
| 18h:35m | 654 |
| 19h:20m | 655 |
| 20h:10m | 657 |
| 20h:55m | 657 |
| 21h:40m | 658 |
| 22h:25m | 657 |
| 23h:15m | 658 |
| 24h:00m | 661 |
| 24h:05m | 529 |
| 24h:50m | 529 |
| 25h:40m | 529 |
| 26h:25m | 533 |
| 27h:10m | 531 |
| 27h:55m | 532 |
| 28h:45m | 531 |
| 29h:30m | 531 |
| 30h:15m | 531 |
| 31h:00m | 531 |
| 31h:50m | 531 |
| 32h:35m | 531 |
| 33h:20m | 532 |
| 34h:05m | 532 |
| 34h:55m | 533 |
| 35h:40m | 531 |
| 36h:25m | 532 |
| 37h:10m | 533 |
| 38h:00m | 534 |
| 38h:45m | 534 |
| 39h:30m | 533 |
| 40h:15m | 532 |
| 41h:05m | 536 |
| 41h:50m | 533 |
| 42h:35m | 531 |
| 43h:20m | 534 |
| 44h:10m | 533 |
| 44h:55m | 534 |
| 45h:40m | 533 |
| 46h:25m | 534 |
| 47h:15m | 532 |
| 48h:00m | 531 |
| 48h:05m | 575 |
| 48h:50m | 571 |
| 49h:40m | 571 |
| 50h:25m | 571 |
| 51h:10m | 573 |
| 51h:55m | 572 |
| 52h:45m | 573 |
| 53h:30m | 572 |
| 54h:15m | 574 |
| 55h:00m | 572 |
| 55h:50m | 573 |
| 56h:35m | 575 |
| 57h:20m | 573 |
| 58h:05m | 572 |
| 58h:55m | 574 |
| 59h:40m | 573 |
| 60h:25m | 574 |
| 61h:10m | 574 |
| 62h:00m | 574 |
| 62h:45m | 574 |
| 63h:30m | 574 |
| 64h:15m | 575 |
| 65h:05m | 573 |
| 65h:50m | 573 |
| 66h:35m | 575 |
| 67h:20m | 574 |
| 68h:10m | 576 |
| 68h:55m | 576 |
| 69h:40m | 574 |
| 70h:25m | 574 |
| 71h:15m | 575 |
| 72h:00m | 576 |

E.Coli Amikacin 4MIC 2

| Time | EC 4MIC AMIK E.3 |
| --- | --- |
| 0h:05m | 802 |
| 0h:50m | 804 |
| 1h:40m | 798 |
| 2h:25m | 794 |
| 3h:10m | 791 |
| 3h:55m | 788 |
| 4h:45m | 790 |
| 5h:30m | 787 |
| 6h:15m | 792 |
| 7h:00m | 790 |
| 7h:50m | 793 |
| 8h:35m | 795 |
| 9h:20m | 792 |
| 10h:05m | 792 |
| 10h:55m | 794 |
| 11h:40m | 794 |
| 12h:25m | 796 |
| 13h:10m | 800 |
| 14h:00m | 805 |
| 14h:45m | 801 |
| 15h:30m | 804 |
| 16h:15m | 808 |
| 17h:05m | 806 |
| 17h:50m | 811 |
| 18h:35m | 813 |
| 19h:20m | 813 |
| 20h:10m | 817 |
| 20h:55m | 819 |
| 21h:40m | 817 |
| 22h:25m | 819 |
| 23h:15m | 823 |
| 24h:00m | 842 |
| 24h:05m | 606 |
| 24h:50m | 617 |
| 25h:40m | 619 |
| 26h:25m | 614 |
| 27h:10m | 615 |
| 27h:55m | 613 |
| 28h:45m | 611 |
| 29h:30m | 609 |
| 30h:15m | 608 |
| 31h:00m | 606 |
| 31h:50m | 606 |
| 32h:35m | 607 |
| 33h:20m | 606 |
| 34h:05m | 607 |
| 34h:55m | 605 |
| 35h:40m | 599 |
| 36h:25m | 598 |
| 37h:10m | 604 |
| 38h:00m | 602 |
| 38h:45m | 604 |
| 39h:30m | 603 |
| 40h:15m | 603 |
| 41h:05m | 601 |
| 41h:50m | 598 |
| 42h:35m | 598 |
| 43h:20m | 598 |
| 44h:10m | 599 |
| 44h:55m | 597 |
| 45h:40m | 598 |
| 46h:25m | 597 |
| 47h:15m | 599 |
| 48h:00m | 600 |
| 48h:05m | 859 |
| 48h:50m | 868 |
| 49h:40m | 871 |
| 50h:25m | 871 |
| 51h:10m | 867 |
| 51h:55m | 864 |
| 52h:45m | 864 |
| 53h:30m | 855 |
| 54h:15m | 859 |
| 55h:00m | 857 |
| 55h:50m | 857 |
| 56h:35m | 852 |
| 57h:20m | 850 |
| 58h:05m | 849 |
| 58h:55m | 851 |
| 59h:40m | 849 |
| 60h:25m | 847 |
| 61h:10m | 849 |
| 62h:00m | 846 |
| 62h:45m | 845 |
| 63h:30m | 847 |
| 64h:15m | 843 |
| 65h:05m | 844 |
| 65h:50m | 840 |
| 66h:35m | 843 |
| 67h:20m | 840 |
| 68h:10m | 839 |
| 68h:55m | 841 |
| 69h:40m | 837 |
| 70h:25m | 840 |
| 71h:15m | 844 |
| 72h:00m | 851 |

E.Coli Amikacin 4MIC 3

| Time | EC 4MIC AMIK E.5 |
| --- | --- |
| 0h:05m | 472 |
| 0h:50m | 474 |
| 1h:40m | 473 |
| 2h:25m | 470 |
| 3h:10m | 468 |
| 3h:55m | 469 |
| 4h:45m | 468 |
| 5h:30m | 467 |
| 6h:15m | 466 |
| 7h:00m | 466 |
| 7h:50m | 467 |
| 8h:35m | 465 |
| 9h:20m | 467 |
| 10h:05m | 466 |
| 10h:55m | 467 |
| 11h:40m | 468 |
| 12h:25m | 467 |
| 13h:10m | 466 |
| 14h:00m | 466 |
| 14h:45m | 468 |
| 15h:30m | 467 |
| 16h:15m | 469 |
| 17h:05m | 467 |
| 17h:50m | 469 |
| 18h:35m | 471 |
| 19h:20m | 471 |
| 20h:10m | 476 |
| 20h:55m | 478 |
| 21h:40m | 483 |
| 22h:25m | 488 |
| 23h:15m | 490 |
| 24h:00m | 493 |
| 24h:05m |  |
| 24h:50m |  |
| 25h:40m |  |
| 26h:25m |  |
| 27h:10m |  |
| 27h:55m |  |
| 28h:45m |  |
| 29h:30m |  |
| 30h:15m |  |
| 31h:00m |  |
| 31h:50m |  |
| 32h:35m |  |
| 33h:20m |  |
| 34h:05m |  |
| 34h:55m |  |
| 35h:40m |  |
| 36h:25m |  |
| 37h:10m |  |
| 38h:00m |  |
| 38h:45m |  |
| 39h:30m |  |
| 40h:15m |  |
| 41h:05m |  |
| 41h:50m |  |
| 42h:35m |  |
| 43h:20m |  |
| 44h:10m |  |
| 44h:55m |  |
| 45h:40m |  |
| 46h:25m |  |
| 47h:15m |  |
| 48h:00m |  |
| 48h:05m | 499 |
| 48h:50m | 500 |
| 49h:40m | 500 |
| 50h:25m | 499 |
| 51h:10m | 499 |
| 51h:55m | 502 |
| 52h:45m | 499 |
| 53h:30m | 499 |
| 54h:15m | 500 |
| 55h:00m | 500 |
| 55h:50m | 497 |
| 56h:35m | 498 |
| 57h:20m | 498 |
| 58h:05m | 499 |
| 58h:55m | 499 |
| 59h:40m | 499 |
| 60h:25m | 498 |
| 61h:10m | 499 |
| 62h:00m | 498 |
| 62h:45m | 497 |
| 63h:30m | 497 |
| 64h:15m | 498 |
| 65h:05m | 500 |
| 65h:50m | 496 |
| 66h:35m | 497 |
| 67h:20m | 496 |
| 68h:10m | 497 |
| 68h:55m | 496 |
| 69h:40m | 496 |
| 70h:25m | 496 |
| 71h:15m | 495 |
| 72h:00m | 493 |

E.Coli Amikacin 4MIC 4

| Time | EC 4MIC AMIK E.5 |
| --- | --- |
| 0h:05m | 480 |
| 0h:50m | 490 |
| 1h:40m | 487 |
| 2h:25m | 484 |
| 3h:10m | 483 |
| 3h:55m | 482 |
| 4h:45m | 483 |
| 5h:30m | 481 |
| 6h:15m | 485 |
| 7h:00m | 481 |
| 7h:50m | 481 |
| 8h:35m | 481 |
| 9h:20m | 481 |
| 10h:05m | 483 |
| 10h:55m | 483 |
| 11h:40m | 483 |
| 12h:25m | 483 |
| 13h:10m | 483 |
| 14h:00m | 483 |
| 14h:45m | 483 |
| 15h:30m | 483 |
| 16h:15m | 483 |
| 17h:05m | 484 |
| 17h:50m | 484 |
| 18h:35m | 485 |
| 19h:20m | 486 |
| 20h:10m | 484 |
| 20h:55m | 485 |
| 21h:40m | 485 |
| 22h:25m | 484 |
| 23h:15m | 485 |
| 24h:00m | 488 |
| 24h:05m | 806 |
| 24h:50m | 806 |
| 25h:40m | 796 |
| 26h:25m | 791 |
| 27h:10m | 789 |
| 27h:55m | 789 |
| 28h:45m | 784 |
| 29h:30m | 783 |
| 30h:15m | 779 |
| 31h:00m | 782 |
| 31h:50m | 779 |
| 32h:35m | 779 |
| 33h:20m | 779 |
| 34h:05m | 778 |
| 34h:55m | 776 |
| 35h:40m | 779 |
| 36h:25m | 778 |
| 37h:10m | 778 |
| 38h:00m | 777 |
| 38h:45m | 776 |
| 39h:30m | 780 |
| 40h:15m | 779 |
| 41h:05m | 782 |
| 41h:50m | 783 |
| 42h:35m | 780 |
| 43h:20m | 778 |
| 44h:10m | 780 |
| 44h:55m | 781 |
| 45h:40m | 782 |
| 46h:25m | 780 |
| 47h:15m | 780 |
| 48h:00m | 779 |
| 48h:05m | 457 |
| 48h:50m | 458 |
| 49h:40m | 456 |
| 50h:25m | 457 |
| 51h:10m | 456 |
| 51h:55m | 456 |
| 52h:45m | 455 |
| 53h:30m | 452 |
| 54h:15m | 454 |
| 55h:00m | 456 |
| 55h:50m | 453 |
| 56h:35m | 453 |
| 57h:20m | 454 |
| 58h:05m | 452 |
| 58h:55m | 450 |
| 59h:40m | 452 |
| 60h:25m | 453 |
| 61h:10m | 452 |
| 62h:00m | 451 |
| 62h:45m | 452 |
| 63h:30m | 452 |
| 64h:15m | 452 |
| 65h:05m | 450 |
| 65h:50m | 452 |
| 66h:35m | 452 |
| 67h:20m | 450 |
| 68h:10m | 451 |
| 68h:55m | 451 |
| 69h:40m | 451 |
| 70h:25m | 452 |
| 71h:15m | 452 |
| 72h:00m | 449 |

E.Coli Amikacin 4MIC 5

| Time | **EC 4MIC AMIK E.4** |
| --- | --- |
| 0h:05m | 710 |
| 0h:50m | 698 |
| 1h:40m | 696 |
| 2h:25m | 695 |
| 3h:10m | 693 |
| 3h:55m | 690 |
| 4h:45m | 689 |
| 5h:30m | 687 |
| 6h:15m | 684 |
| 7h:00m | 684 |
| 7h:50m | 683 |
| 8h:35m | 683 |
| 9h:20m | 681 |
| 10h:05m | 680 |
| 10h:55m | 681 |
| 11h:40m | 685 |
| 12h:25m | 692 |
| 13h:10m | 699 |
| 14h:00m | 709 |
| 14h:45m | 717 |
| 15h:30m | 726 |
| 16h:15m | 732 |
| 17h:05m | 739 |
| 17h:50m | 750 |
| 18h:35m | 808 |
| 19h:20m | 992 |
| 20h:10m | 1600 |
| 20h:55m | 3167 |
| 21h:40m | 7099 |
| 22h:25m | 14829 |
| 23h:15m | 30987 |
| 24h:00m | 30961 |
| 24h:05m |  |
| 24h:50m |  |
| 25h:40m |  |
| 26h:25m |  |
| 27h:10m |  |
| 27h:55m |  |
| 28h:45m |  |
| 29h:30m |  |
| 30h:15m |  |
| 31h:00m |  |
| 31h:50m |  |
| 32h:35m |  |
| 33h:20m |  |
| 34h:05m |  |
| 34h:55m |  |
| 35h:40m |  |
| 36h:25m |  |
| 37h:10m |  |
| 38h:00m |  |
| 38h:45m |  |
| 39h:30m |  |
| 40h:15m |  |
| 41h:05m |  |
| 41h:50m |  |
| 42h:35m |  |
| 43h:20m |  |
| 44h:10m |  |
| 44h:55m |  |
| 45h:40m |  |
| 46h:25m |  |
| 47h:15m |  |
| 48h:00m |  |
| 48h:05m |  |
| 48h:50m |  |
| 49h:40m |  |
| 50h:25m |  |
| 51h:10m |  |
| 51h:55m |  |
| 52h:45m |  |
| 53h:30m |  |
| 54h:15m |  |
| 55h:00m |  |
| 55h:50m |  |
| 56h:35m |  |
| 57h:20m |  |
| 58h:05m |  |
| 58h:55m |  |
| 59h:40m |  |
| 60h:25m |  |
| 61h:10m |  |
| 62h:00m |  |
| 62h:45m |  |
| 63h:30m |  |
| 64h:15m |  |
| 65h:05m |  |
| 65h:50m |  |
| 66h:35m |  |
| 67h:20m |  |
| 68h:10m |  |
| 68h:55m |  |
| 69h:40m |  |
| 70h:25m |  |
| 71h:15m |  |
| 72h:00m |  |

E.Coli Amikacin 4MIC 6

| Time | EC 4MIC AMIK E.4 |
| --- | --- |
| 0h:05m | 657 |
| 0h:50m | 647 |
| 1h:40m | 646 |
| 2h:25m | 644 |
| 3h:10m | 641 |
| 3h:55m | 639 |
| 4h:45m | 637 |
| 5h:30m | 635 |
| 6h:15m | 634 |
| 7h:00m | 634 |
| 7h:50m | 633 |
| 8h:35m | 632 |
| 9h:20m | 630 |
| 10h:05m | 629 |
| 10h:55m | 628 |
| 11h:40m | 627 |
| 12h:25m | 628 |
| 13h:10m | 627 |
| 14h:00m | 627 |
| 14h:45m | 627 |
| 15h:30m | 629 |
| 16h:15m | 629 |
| 17h:05m | 631 |
| 17h:50m | 634 |
| 18h:35m | 634 |
| 19h:20m | 635 |
| 20h:10m | 635 |
| 20h:55m | 638 |
| 21h:40m | 640 |
| 22h:25m | 641 |
| 23h:15m | 645 |
| 24h:00m | 651 |
| 24h:05m | 633 |
| 24h:50m | 652 |
| 25h:40m | 709 |
| 26h:25m | 784 |
| 27h:10m | 949 |
| 27h:55m | 1117 |
| 28h:45m | 1585 |
| 29h:30m | 2445 |
| 30h:15m | 4719 |
| 31h:00m | 7672 |
| 31h:50m | 18619 |
| 32h:35m | 30902 |
| 33h:20m | 30953 |
| 34h:05m | 30929 |
| 34h:55m | 30930 |
| 35h:40m | 30930 |
| 36h:25m | 30930 |
| 37h:10m | 30929 |
| 38h:00m | 30930 |
| 38h:45m | 30932 |
| 39h:30m | 30933 |
| 40h:15m | 30934 |
| 41h:05m | 30934 |
| 41h:50m | 30934 |
| 42h:35m | 30934 |
| 43h:20m | 30934 |
| 44h:10m | 30932 |
| 44h:55m | 30933 |
| 45h:40m | 30933 |
| 46h:25m | 30933 |
| 47h:15m | 30934 |
| 48h:00m | 30933 |
| 48h:05m |  |
| 48h:50m |  |
| 49h:40m |  |
| 50h:25m |  |
| 51h:10m |  |
| 51h:55m |  |
| 52h:45m |  |
| 53h:30m |  |
| 54h:15m |  |
| 55h:00m |  |
| 55h:50m |  |
| 56h:35m |  |
| 57h:20m |  |
| 58h:05m |  |
| 58h:55m |  |
| 59h:40m |  |
| 60h:25m |  |
| 61h:10m |  |
| 62h:00m |  |
| 62h:45m |  |
| 63h:30m |  |
| 64h:15m |  |
| 65h:05m |  |
| 65h:50m |  |
| 66h:35m |  |
| 67h:20m |  |
| 68h:10m |  |
| 68h:55m |  |
| 69h:40m |  |
| 70h:25m |  |
| 71h:15m |  |
| 72h:00m |  |

E.Coli Amikacin 1000MIC 1

| Time | EC 1000MIC AMIK E.3 |
| --- | --- |
| 0h:05m | 1004 |
| 0h:50m | 1018 |
| 1h:40m | 1033 |
| 2h:25m | 1038 |
| 3h:10m | 1042 |
| 3h:55m | 1045 |
| 4h:45m | 1045 |
| 5h:30m | 1045 |
| 6h:15m | 1048 |
| 7h:00m | 1044 |
| 7h:50m | 1045 |
| 8h:35m | 1045 |
| 9h:20m | 1047 |
| 10h:05m | 1045 |
| 10h:55m | 1044 |
| 11h:40m | 1041 |
| 12h:25m | 1042 |
| 13h:10m | 1042 |
| 14h:00m | 1039 |
| 14h:45m | 1039 |
| 15h:30m | 1039 |
| 16h:15m | 1039 |
| 17h:05m | 1036 |
| 17h:50m | 1038 |
| 18h:35m | 1038 |
| 19h:20m | 1038 |
| 20h:10m | 1038 |
| 20h:55m | 1041 |
| 21h:40m | 1036 |
| 22h:25m | 1038 |
| 23h:15m | 1035 |
| 24h:00m | 1042 |
| 24h:05m | 604 |
| 24h:50m | 605 |
| 25h:40m | 606 |
| 26h:25m | 607 |
| 27h:10m | 606 |
| 27h:55m | 607 |
| 28h:45m | 604 |
| 29h:30m | 605 |
| 30h:15m | 608 |
| 31h:00m | 605 |
| 31h:50m | 606 |
| 32h:35m | 606 |
| 33h:20m | 606 |
| 34h:05m | 606 |
| 34h:55m | 606 |
| 35h:40m | 606 |
| 36h:25m | 606 |
| 37h:10m | 604 |
| 38h:00m | 606 |
| 38h:45m | 605 |
| 39h:30m | 607 |
| 40h:15m | 605 |
| 41h:05m | 605 |
| 41h:50m | 606 |
| 42h:35m | 606 |
| 43h:20m | 604 |
| 44h:10m | 606 |
| 44h:55m | 606 |
| 45h:40m | 606 |
| 46h:25m | 607 |
| 47h:15m | 607 |
| 48h:00m | 605 |
| 48h:05m | 895 |
| 48h:50m | 892 |
| 49h:40m | 891 |
| 50h:25m | 891 |
| 51h:10m | 893 |
| 51h:55m | 891 |
| 52h:45m | 891 |
| 53h:30m | 892 |
| 54h:15m | 891 |
| 55h:00m | 891 |
| 55h:50m | 894 |
| 56h:35m | 893 |
| 57h:20m | 892 |
| 58h:05m | 893 |
| 58h:55m | 890 |
| 59h:40m | 893 |
| 60h:25m | 888 |
| 61h:10m | 890 |
| 62h:00m | 889 |
| 62h:45m | 891 |
| 63h:30m | 888 |
| 64h:15m | 891 |
| 65h:05m | 889 |
| 65h:50m | 890 |
| 66h:35m | 887 |
| 67h:20m | 889 |
| 68h:10m | 886 |
| 68h:55m | 887 |
| 69h:40m | 886 |
| 70h:25m | 887 |
| 71h:15m | 887 |
| 72h:00m | 892 |

E.Coli Amikacin 1000MIC 2

| Time | EC 1000MIC AMIK E.3 |
| --- | --- |
| 0h:05m | 642 |
| 0h:50m | 665 |
| 1h:40m | 675 |
| 2h:25m | 677 |
| 3h:10m | 680 |
| 3h:55m | 676 |
| 4h:45m | 683 |
| 5h:30m | 685 |
| 6h:15m | 681 |
| 7h:00m | 677 |
| 7h:50m | 678 |
| 8h:35m | 681 |
| 9h:20m | 677 |
| 10h:05m | 680 |
| 10h:55m | 679 |
| 11h:40m | 677 |
| 12h:25m | 677 |
| 13h:10m | 674 |
| 14h:00m | 671 |
| 14h:45m | 672 |
| 15h:30m | 672 |
| 16h:15m | 671 |
| 17h:05m | 674 |
| 17h:50m | 671 |
| 18h:35m | 668 |
| 19h:20m | 667 |
| 20h:10m | 669 |
| 20h:55m | 670 |
| 21h:40m | 671 |
| 22h:25m | 669 |
| 23h:15m | 673 |
| 24h:00m | 674 |
| 24h:05m | 1009 |
| 24h:50m | 1013 |
| 25h:40m | 1011 |
| 26h:25m | 1010 |
| 27h:10m | 1014 |
| 27h:55m | 1020 |
| 28h:45m | 1018 |
| 29h:30m | 1022 |
| 30h:15m | 1019 |
| 31h:00m | 1017 |
| 31h:50m | 1036 |
| 32h:35m | 1038 |
| 33h:20m | 1040 |
| 34h:05m | 1040 |
| 34h:55m | 1038 |
| 35h:40m | 1038 |
| 36h:25m | 1041 |
| 37h:10m | 1040 |
| 38h:00m | 1037 |
| 38h:45m | 1036 |
| 39h:30m | 1036 |
| 40h:15m | 1036 |
| 41h:05m | 1036 |
| 41h:50m | 1044 |
| 42h:35m | 1046 |
| 43h:20m | 1037 |
| 44h:10m | 1043 |
| 44h:55m | 1038 |
| 45h:40m | 1050 |
| 46h:25m | 1046 |
| 47h:15m | 1043 |
| 48h:00m | 1039 |
| 48h:05m | 724 |
| 48h:50m | 731 |
| 49h:40m | 725 |
| 50h:25m | 725 |
| 51h:10m | 728 |
| 51h:55m | 725 |
| 52h:45m | 728 |
| 53h:30m | 727 |
| 54h:15m | 725 |
| 55h:00m | 726 |
| 55h:50m | 726 |
| 56h:35m | 724 |
| 57h:20m | 725 |
| 58h:05m | 722 |
| 58h:55m | 725 |
| 59h:40m | 726 |
| 60h:25m | 723 |
| 61h:10m | 722 |
| 62h:00m | 724 |
| 62h:45m | 723 |
| 63h:30m | 723 |
| 64h:15m | 722 |
| 65h:05m | 721 |
| 65h:50m | 723 |
| 66h:35m | 722 |
| 67h:20m | 719 |
| 68h:10m | 719 |
| 68h:55m | 719 |
| 69h:40m | 719 |
| 70h:25m | 723 |
| 71h:15m | 720 |
| 72h:00m | 722 |

E.Coli Amikacin 1000MIC 3

| Time | EC 1000MIC AMIK E.5 |
| --- | --- |
| 0h:05m | 513 |
| 0h:50m | 528 |
| 1h:40m | 547 |
| 2h:25m | 555 |
| 3h:10m | 557 |
| 3h:55m | 560 |
| 4h:45m | 559 |
| 5h:30m | 563 |
| 6h:15m | 562 |
| 7h:00m | 561 |
| 7h:50m | 563 |
| 8h:35m | 561 |
| 9h:20m | 562 |
| 10h:05m | 560 |
| 10h:55m | 565 |
| 11h:40m | 563 |
| 12h:25m | 561 |
| 13h:10m | 560 |
| 14h:00m | 559 |
| 14h:45m | 560 |
| 15h:30m | 561 |
| 16h:15m | 559 |
| 17h:05m | 557 |
| 17h:50m | 559 |
| 18h:35m | 557 |
| 19h:20m | 558 |
| 20h:10m | 558 |
| 20h:55m | 557 |
| 21h:40m | 559 |
| 22h:25m | 560 |
| 23h:15m | 561 |
| 24h:00m | 560 |
| 24h:05m |  |
| 24h:50m |  |
| 25h:40m |  |
| 26h:25m |  |
| 27h:10m |  |
| 27h:55m |  |
| 28h:45m |  |
| 29h:30m |  |
| 30h:15m |  |
| 31h:00m |  |
| 31h:50m |  |
| 32h:35m |  |
| 33h:20m |  |
| 34h:05m |  |
| 34h:55m |  |
| 35h:40m |  |
| 36h:25m |  |
| 37h:10m |  |
| 38h:00m |  |
| 38h:45m |  |
| 39h:30m |  |
| 40h:15m |  |
| 41h:05m |  |
| 41h:50m |  |
| 42h:35m |  |
| 43h:20m |  |
| 44h:10m |  |
| 44h:55m |  |
| 45h:40m |  |
| 46h:25m |  |
| 47h:15m |  |
| 48h:00m |  |
| 48h:05m | 587 |
| 48h:50m | 584 |
| 49h:40m | 582 |
| 50h:25m | 584 |
| 51h:10m | 584 |
| 51h:55m | 583 |
| 52h:45m | 585 |
| 53h:30m | 581 |
| 54h:15m | 582 |
| 55h:00m | 581 |
| 55h:50m | 581 |
| 56h:35m | 581 |
| 57h:20m | 581 |
| 58h:05m | 581 |
| 58h:55m | 582 |
| 59h:40m | 581 |
| 60h:25m | 582 |
| 61h:10m | 581 |
| 62h:00m | 581 |
| 62h:45m | 582 |
| 63h:30m | 583 |
| 64h:15m | 584 |
| 65h:05m | 583 |
| 65h:50m | 584 |
| 66h:35m | 584 |
| 67h:20m | 586 |
| 68h:10m | 587 |
| 68h:55m | 584 |
| 69h:40m | 581 |
| 70h:25m | 586 |
| 71h:15m | 585 |
| 72h:00m | 582 |

E.Coli Amikacin 1000MIC 4

| Time | EC 1000MIC AMIK E.5 |
| --- | --- |
| 0h:05m | 518 |
| 0h:50m | 538 |
| 1h:40m | 558 |
| 2h:25m | 565 |
| 3h:10m | 567 |
| 3h:55m | 569 |
| 4h:45m | 571 |
| 5h:30m | 571 |
| 6h:15m | 570 |
| 7h:00m | 570 |
| 7h:50m | 570 |
| 8h:35m | 571 |
| 9h:20m | 569 |
| 10h:05m | 571 |
| 10h:55m | 570 |
| 11h:40m | 570 |
| 12h:25m | 569 |
| 13h:10m | 569 |
| 14h:00m | 570 |
| 14h:45m | 569 |
| 15h:30m | 571 |
| 16h:15m | 569 |
| 17h:05m | 572 |
| 17h:50m | 569 |
| 18h:35m | 567 |
| 19h:20m | 569 |
| 20h:10m | 569 |
| 20h:55m | 567 |
| 21h:40m | 568 |
| 22h:25m | 570 |
| 23h:15m | 572 |
| 24h:00m | 571 |
| 24h:05m |  |
| 24h:50m |  |
| 25h:40m |  |
| 26h:25m |  |
| 27h:10m |  |
| 27h:55m |  |
| 28h:45m |  |
| 29h:30m |  |
| 30h:15m |  |
| 31h:00m |  |
| 31h:50m |  |
| 32h:35m |  |
| 33h:20m |  |
| 34h:05m |  |
| 34h:55m |  |
| 35h:40m |  |
| 36h:25m |  |
| 37h:10m |  |
| 38h:00m |  |
| 38h:45m |  |
| 39h:30m |  |
| 40h:15m |  |
| 41h:05m |  |
| 41h:50m |  |
| 42h:35m |  |
| 43h:20m |  |
| 44h:10m |  |
| 44h:55m |  |
| 45h:40m |  |
| 46h:25m |  |
| 47h:15m |  |
| 48h:00m |  |
| 48h:05m | 623 |
| 48h:50m | 614 |
| 49h:40m | 612 |
| 50h:25m | 612 |
| 51h:10m | 613 |
| 51h:55m | 610 |
| 52h:45m | 610 |
| 53h:30m | 612 |
| 54h:15m | 610 |
| 55h:00m | 608 |
| 55h:50m | 608 |
| 56h:35m | 611 |
| 57h:20m | 608 |
| 58h:05m | 609 |
| 58h:55m | 612 |
| 59h:40m | 606 |
| 60h:25m | 611 |
| 61h:10m | 610 |
| 62h:00m | 608 |
| 62h:45m | 608 |
| 63h:30m | 608 |
| 64h:15m | 609 |
| 65h:05m | 609 |
| 65h:50m | 606 |
| 66h:35m | 611 |
| 67h:20m | 609 |
| 68h:10m | 608 |
| 68h:55m | 608 |
| 69h:40m | 610 |
| 70h:25m | 608 |
| 71h:15m | 610 |
| 72h:00m | 609 |

E.Coli Amikacin 1000MIC 5

| Time | EC 1000MIC AMIK E.4 |
| --- | --- |
| 0h:05m | 672 |
| 0h:50m | 681 |
| 1h:40m | 686 |
| 2h:25m | 687 |
| 3h:10m | 689 |
| 3h:55m | 690 |
| 4h:45m | 691 |
| 5h:30m | 691 |
| 6h:15m | 692 |
| 7h:00m | 689 |
| 7h:50m | 691 |
| 8h:35m | 690 |
| 9h:20m | 690 |
| 10h:05m | 688 |
| 10h:55m | 689 |
| 11h:40m | 694 |
| 12h:25m | 694 |
| 13h:10m | 691 |
| 14h:00m | 695 |
| 14h:45m | 692 |
| 15h:30m | 691 |
| 16h:15m | 694 |
| 17h:05m | 690 |
| 17h:50m | 691 |
| 18h:35m | 691 |
| 19h:20m | 692 |
| 20h:10m | 692 |
| 20h:55m | 692 |
| 21h:40m | 692 |
| 22h:25m | 690 |
| 23h:15m | 694 |
| 24h:00m | 691 |
| 24h:05m | 806 |
| 24h:50m | 806 |
| 25h:40m | 796 |
| 26h:25m | 791 |
| 27h:10m | 789 |
| 27h:55m | 789 |
| 28h:45m | 784 |
| 29h:30m | 783 |
| 30h:15m | 779 |
| 31h:00m | 782 |
| 31h:50m | 779 |
| 32h:35m | 779 |
| 33h:20m | 779 |
| 34h:05m | 778 |
| 34h:55m | 776 |
| 35h:40m | 779 |
| 36h:25m | 778 |
| 37h:10m | 778 |
| 38h:00m | 777 |
| 38h:45m | 776 |
| 39h:30m | 780 |
| 40h:15m | 779 |
| 41h:05m | 782 |
| 41h:50m | 783 |
| 42h:35m | 780 |
| 43h:20m | 778 |
| 44h:10m | 780 |
| 44h:55m | 781 |
| 45h:40m | 782 |
| 46h:25m | 780 |
| 47h:15m | 780 |
| 48h:00m | 779 |
| 48h:05m | 620 |
| 48h:50m | 621 |
| 49h:40m | 622 |
| 50h:25m | 623 |
| 51h:10m | 622 |
| 51h:55m | 622 |
| 52h:45m | 622 |
| 53h:30m | 624 |
| 54h:15m | 622 |
| 55h:00m | 622 |
| 55h:50m | 624 |
| 56h:35m | 622 |
| 57h:20m | 625 |
| 58h:05m | 625 |
| 58h:55m | 622 |
| 59h:40m | 623 |
| 60h:25m | 625 |
| 61h:10m | 623 |
| 62h:00m | 625 |
| 62h:45m | 625 |
| 63h:30m | 624 |
| 64h:15m | 624 |
| 65h:05m | 628 |
| 65h:50m | 623 |
| 66h:35m | 625 |
| 67h:20m | 627 |
| 68h:10m | 625 |
| 68h:55m | 626 |
| 69h:40m | 624 |
| 70h:25m | 623 |
| 71h:15m | 622 |
| 72h:00m | 622 |

E.Coli Amikacin 1000MIC 6

| Time | EC 1000MIC AMIK E.4 |
| --- | --- |
| 0h:05m | 950 |
| 0h:50m | 950 |
| 1h:40m | 952 |
| 2h:25m | 953 |
| 3h:10m | 953 |
| 3h:55m | 956 |
| 4h:45m | 955 |
| 5h:30m | 960 |
| 6h:15m | 959 |
| 7h:00m | 960 |
| 7h:50m | 958 |
| 8h:35m | 960 |
| 9h:20m | 960 |
| 10h:05m | 961 |
| 10h:55m | 964 |
| 11h:40m | 961 |
| 12h:25m | 962 |
| 13h:10m | 961 |
| 14h:00m | 961 |
| 14h:45m | 961 |
| 15h:30m | 961 |
| 16h:15m | 963 |
| 17h:05m | 963 |
| 17h:50m | 962 |
| 18h:35m | 961 |
| 19h:20m | 958 |
| 20h:10m | 956 |
| 20h:55m | 960 |
| 21h:40m | 956 |
| 22h:25m | 958 |
| 23h:15m | 959 |
| 24h:00m | 933 |
| 24h:05m | 1277 |
| 24h:50m | 1276 |
| 25h:40m | 1274 |
| 26h:25m | 1267 |
| 27h:10m | 1274 |
| 27h:55m | 1275 |
| 28h:45m | 1273 |
| 29h:30m | 1275 |
| 30h:15m | 1276 |
| 31h:00m | 1275 |
| 31h:50m | 1277 |
| 32h:35m | 1274 |
| 33h:20m | 1275 |
| 34h:05m | 1277 |
| 34h:55m | 1273 |
| 35h:40m | 1269 |
| 36h:25m | 1272 |
| 37h:10m | 1275 |
| 38h:00m | 1272 |
| 38h:45m | 1275 |
| 39h:30m | 1282 |
| 40h:15m | 1281 |
| 41h:05m | 1280 |
| 41h:50m | 1269 |
| 42h:35m | 1266 |
| 43h:20m | 1271 |
| 44h:10m | 1273 |
| 44h:55m | 1275 |
| 45h:40m | 1276 |
| 46h:25m | 1272 |
| 47h:15m | 1272 |
| 48h:00m | 1270 |
| 48h:05m | 701 |
| 48h:50m | 701 |
| 49h:40m | 702 |
| 50h:25m | 703 |
| 51h:10m | 703 |
| 51h:55m | 702 |
| 52h:45m | 703 |
| 53h:30m | 704 |
| 54h:15m | 704 |
| 55h:00m | 703 |
| 55h:50m | 704 |
| 56h:35m | 704 |
| 57h:20m | 705 |
| 58h:05m | 705 |
| 58h:55m | 706 |
| 59h:40m | 706 |
| 60h:25m | 706 |
| 61h:10m | 706 |
| 62h:00m | 706 |
| 62h:45m | 703 |
| 63h:30m | 709 |
| 64h:15m | 706 |
| 65h:05m | 704 |
| 65h:50m | 707 |
| 66h:35m | 707 |
| 67h:20m | 705 |
| 68h:10m | 705 |
| 68h:55m | 701 |
| 69h:40m | 705 |
| 70h:25m | 705 |
| 71h:15m | 708 |
| 72h:00m | 703 |

Staph Aureus Vancomycin control 1

| Time | SA Cont |
| --- | --- |
| 0h:05m | 588 |
| 0h:50m | 606 |
| 1h:40m | 706 |
| 2h:25m | 1155 |
| 3h:10m | 3904 |
| 3h:55m | 12311 |
| 4h:45m | 30985 |
| 5h:30m | 30933 |
| 6h:15m | 30919 |
| 7h:00m | 30919 |
| 7h:50m | 30923 |
| 8h:35m | 30923 |
| 9h:20m | 30924 |
| 10h:05m | 30924 |
| 10h:55m | 30923 |
| 11h:40m | 30923 |
| 12h:25m | 30923 |
| 13h:10m | 30923 |
| 14h:00m | 30923 |
| 14h:45m | 30923 |
| 15h:30m | 30923 |
| 16h:15m | 30923 |
| 17h:05m | 30924 |
| 17h:50m | 30924 |
| 18h:35m | 30923 |
| 19h:20m | 30924 |
| 20h:10m | 30924 |
| 20h:55m | 30924 |
| 21h:40m | 30923 |
| 22h:25m | 30923 |
| 23h:15m | 30921 |
| 24h:00m | 30920 |
| 24h:05m | |
| 24h:50m | |
| 25h:40m | |
| 26h:25m | |
| 27h:10m | |
| 27h:55m | |
| 28h:45m | |
| 29h:30m | |
| 30h:15m | |
| 31h:00m | |
| 31h:50m | |
| 32h:35m | |
| 33h:20m | |
| 34h:05m | |
| 34h:55m | |
| 35h:40m | |
| 36h:25m | |
| 37h:10m | |
| 38h:00m | |
| 38h:45m | |
| 39h:30m | |
| 40h:15m | |
| 41h:05m | |
| 41h:50m | |
| 42h:35m | |
| 43h:20m | |
| 44h:10m | |
| 44h:55m | |
| 45h:40m | |
| 46h:25m | |
| 47h:15m | |
| 48h:00m | |
| 48h:05m |  |
| 48h:50m | |
| 49h:40m | |
| 50h:25m | |
| 51h:10m | |
| 51h:55m | |
| 52h:45m | |
| 53h:30m | |
| 54h:15m | |
| 55h:00m | |
| 55h:50m | |
| 56h:35m | |
| 57h:20m | |
| 58h:05m | |
| 58h:55m | |
| 59h:40m | |
| 60h:25m | |
| 61h:10m | |
| 62h:00m | |
| 62h:45m | |
| 63h:30m | |
| 64h:15m | |
| 65h:05m | |
| 65h:50m | |
| 66h:35m | |
| 67h:20m | |
| 68h:10m | |
| 68h:55m | |
| 69h:40m | |
| 70h:25m | |
| 71h:15m | |
| 72h:00m | |

Staph Aureus Vancomycin control 2

| Time | SA Cont EXP.4 |
| --- | --- |
| 0h:05m | 892 |
| 0h:50m | 922 |
| 1h:40m | 1042 |
| 2h:25m | 1971 |
| 3h:10m | 10017 |
| 3h:55m | 30977 |
| 4h:45m | 30922 |
| 5h:30m | 30923 |
| 6h:15m | 30924 |
| 7h:00m | 30925 |
| 7h:50m | 30926 |
| 8h:35m | 30926 |
| 9h:20m | 30927 |
| 10h:05m | 30928 |
| 10h:55m | 30930 |
| 11h:40m | 30930 |
| 12h:25m | 30931 |
| 13h:10m | 30931 |
| 14h:00m | 30931 |
| 14h:45m | 30930 |
| 15h:30m | 30930 |
| 16h:15m | 30930 |
| 17h:05m | 30930 |
| 17h:50m | 30930 |
| 18h:35m | 30930 |
| 19h:20m | 30929 |
| 20h:10m | 30927 |
| 20h:55m | 30927 |
| 21h:40m | 30926 |
| 22h:25m | 30926 |
| 23h:15m | 30926 |
| 24h:00m | 30926 |
| 24h:05m |  |
| 24h:50m |  |
| 25h:40m |  |
| 26h:25m |  |
| 27h:10m |  |
| 27h:55m |  |
| 28h:45m |  |
| 29h:30m |  |
| 30h:15m |  |
| 31h:00m |  |
| 31h:50m |  |
| 32h:35m |  |
| 33h:20m |  |
| 34h:05m |  |
| 34h:55m |  |
| 35h:40m |  |
| 36h:25m |  |
| 37h:10m |  |
| 38h:00m |  |
| 38h:45m |  |
| 39h:30m |  |
| 40h:15m |  |
| 41h:05m |  |
| 41h:50m |  |
| 42h:35m |  |
| 43h:20m |  |
| 44h:10m |  |
| 44h:55m |  |
| 45h:40m |  |
| 46h:25m |  |
| 47h:15m |  |
| 48h:00m |  |
| 48h:05m |  |
| 48h:50m |  |
| 49h:40m |  |
| 50h:25m |  |
| 51h:10m |  |
| 51h:55m |  |
| 52h:45m |  |
| 53h:30m |  |
| 54h:15m |  |
| 55h:00m |  |
| 55h:50m |  |
| 56h:35m |  |
| 57h:20m |  |
| 58h:05m |  |
| 58h:55m |  |
| 59h:40m |  |
| 60h:25m |  |
| 61h:10m |  |
| 62h:00m |  |
| 62h:45m |  |
| 63h:30m |  |
| 64h:15m |  |
| 65h:05m |  |
| 65h:50m |  |
| 66h:35m |  |
| 67h:20m |  |
| 68h:10m |  |
| 68h:55m |  |
| 69h:40m |  |
| 70h:25m |  |
| 71h:15m |  |
| 72h:00m |  |

Staph Aureus Vancomycin control 3

| Time | SA Cont EXP.8 |
| --- | --- |
| 0h:05m | 496 |
| 0h:50m | 490 |
| 1h:40m | 541 |
| 2h:25m | 1104 |
| 3h:10m | 4624 |
| 3h:55m | 25873 |
| 4h:45m | 30985 |
| 5h:30m | 30975 |
| 6h:15m | 30975 |
| 7h:00m | 30975 |
| 7h:50m | 30974 |
| 8h:35m | 30975 |
| 9h:20m | 30975 |
| 10h:05m | 30975 |
| 10h:55m | 30976 |
| 11h:40m | 30977 |
| 12h:25m | 30978 |
| 13h:10m | 30979 |
| 14h:00m | 30979 |
| 14h:45m | 30979 |
| 15h:30m | 30980 |
| 16h:15m | 30980 |
| 17h:05m | 30982 |
| 17h:50m | 30982 |
| 18h:35m | 30980 |
| 19h:20m | 30977 |
| 20h:10m | 30977 |
| 20h:55m | 30976 |
| 21h:40m | 30975 |
| 22h:25m | 30975 |
| 23h:15m | 30974 |
| 24h:00m | 30974 |
| 24h:05m |  |
| 24h:50m |  |
| 25h:40m |  |
| 26h:25m |  |
| 27h:10m |  |
| 27h:55m |  |
| 28h:45m |  |
| 29h:30m |  |
| 30h:15m |  |
| 31h:00m |  |
| 31h:50m |  |
| 32h:35m |  |
| 33h:20m |  |
| 34h:05m |  |
| 34h:55m |  |
| 35h:40m |  |
| 36h:25m |  |
| 37h:10m |  |
| 38h:00m |  |
| 38h:45m |  |
| 39h:30m |  |
| 40h:15m |  |
| 41h:05m |  |
| 41h:50m |  |
| 42h:35m |  |
| 43h:20m |  |
| 44h:10m |  |
| 44h:55m |  |
| 45h:40m |  |
| 46h:25m |  |
| 47h:15m |  |
| 48h:00m |  |
| 48h:05m |  |
| 48h:50m |  |
| 49h:40m |  |
| 50h:25m |  |
| 51h:10m |  |
| 51h:55m |  |
| 52h:45m |  |
| 53h:30m |  |
| 54h:15m |  |
| 55h:00m |  |
| 55h:50m |  |
| 56h:35m |  |
| 57h:20m |  |
| 58h:05m |  |
| 58h:55m |  |
| 59h:40m |  |
| 60h:25m |  |
| 61h:10m |  |
| 62h:00m |  |
| 62h:45m |  |
| 63h:30m |  |
| 64h:15m |  |
| 65h:05m |  |
| 65h:50m |  |
| 66h:35m |  |
| 67h:20m |  |
| 68h:10m |  |
| 68h:55m |  |
| 69h:40m |  |
| 70h:25m |  |
| 71h:15m |  |
| 72h:00m |  |

Staph Aureus Vancomycin control 4

| Time | SA Cont EXP.W |
| --- | --- |
| 0h:05m | 305 |
| 0h:50m | 286 |
| 1h:40m | 336 |
| 2h:25m | 524 |
| 3h:10m | 1712 |
| 3h:55m | 7299 |
| 4h:45m | 26441 |
| 5h:30m | 30949 |
| 6h:15m | 30918 |
| 7h:00m | 30915 |
| 7h:50m | 30921 |
| 8h:35m | 30920 |
| 9h:20m | 30918 |
| 10h:05m | 30918 |
| 10h:55m | 30919 |
| 11h:40m | 30917 |
| 12h:25m | 30916 |
| 13h:10m | 30918 |
| 14h:00m | 30918 |
| 14h:45m | 30919 |
| 15h:30m | 30919 |
| 16h:15m | 30918 |
| 17h:05m | 30917 |
| 17h:50m | 30917 |
| 18h:35m | 30917 |
| 19h:20m | 30918 |
| 20h:10m | 30918 |
| 20h:55m | 30920 |
| 21h:40m | 30917 |
| 22h:25m | 30917 |
| 23h:15m | 30920 |
| 24h:00m | 30918 |
| 24h:05m |  |
| 24h:50m |  |
| 25h:40m |  |
| 26h:25m |  |
| 27h:10m |  |
| 27h:55m |  |
| 28h:45m |  |
| 29h:30m |  |
| 30h:15m |  |
| 31h:00m |  |
| 31h:50m |  |
| 32h:35m |  |
| 33h:20m |  |
| 34h:05m |  |
| 34h:55m |  |
| 35h:40m |  |
| 36h:25m |  |
| 37h:10m |  |
| 38h:00m |  |
| 38h:45m |  |
| 39h:30m |  |
| 40h:15m |  |
| 41h:05m |  |
| 41h:50m |  |
| 42h:35m |  |
| 43h:20m |  |
| 44h:10m |  |
| 44h:55m |  |
| 45h:40m |  |
| 46h:25m |  |
| 47h:15m |  |
| 48h:00m |  |
| 48h:05m |  |
| 48h:50m |  |
| 49h:40m |  |
| 50h:25m |  |
| 51h:10m |  |
| 51h:55m |  |
| 52h:45m |  |
| 53h:30m |  |
| 54h:15m |  |
| 55h:00m |  |
| 55h:50m |  |
| 56h:35m |  |
| 57h:20m |  |
| 58h:05m |  |
| 58h:55m |  |
| 59h:40m |  |
| 60h:25m |  |
| 61h:10m |  |
| 62h:00m |  |
| 62h:45m |  |
| 63h:30m |  |
| 64h:15m |  |
| 65h:05m |  |
| 65h:50m |  |
| 66h:35m |  |
| 67h:20m |  |
| 68h:10m |  |
| 68h:55m |  |
| 69h:40m |  |
| 70h:25m |  |
| 71h:15m |  |
| 72h:00m |  |

Staph Aureus Vancomycin control 5

| Time | Control | |
| --- | --- | --- |
| 00:05 | 405 |  |
| 02:25 | 994 |  |
| 04:45 | 30941 |  |
| 07:05 | 30926 |  |
| 09:20 | 30931 |  |
| 11:40 | 30928 |  |
| 14:00 | 30936 |  |
| 16:20 | 30938 |  |
| 18:40 | 30938 |  |
| 21:00 | 30938 |  |
| 23:15 | 30931 |  |
| 25:35 | 30929 |  |
| 27:55 | 30930 |  |
| 30:15 | 30930 |  |
| 32:35 | 30930 |  |
| 34:55 | 30928 |  |
| 37:10 | 30928 |  |
| 39:30 | 30929 |  |
| 41:50 | 30930 |  |
| 44:10 | 30931 |  |
| 46:30 | 30932 |  |
| 48:50 | 30933 |  |
| 51:05 | 30936 |  |
| 53:25 | 30936 |  |
| 55:45 | 30940 |  |
| 58:05 | 30936 |  |
| 60:25 | 30941 |  |
| 62:45 | 30942 |  |
| 65:00 | 30944 |  |
| 67:20 | 30945 |  |
| 69:40 | 30949 |  |
| 72:00 | 30943 |  |

Staph Aureus Vancomycin 1MIC 1

| Time | SA 1MIC VANCO |
| --- | --- |
| 0h:05m | 645 |
| 0h:50m | 654 |
| 1h:40m | 656 |
| 2h:25m | 657 |
| 3h:10m | 656 |
| 3h:55m | 653 |
| 4h:45m | 653 |
| 5h:30m | 652 |
| 6h:15m | 653 |
| 7h:00m | 650 |
| 7h:50m | 650 |
| 8h:35m | 649 |
| 9h:20m | 649 |
| 10h:05m | 648 |
| 10h:55m | 649 |
| 11h:40m | 648 |
| 12h:25m | 650 |
| 13h:10m | 652 |
| 14h:00m | 651 |
| 14h:45m | 658 |
| 15h:30m | 683 |
| 16h:15m | 705 |
| 17h:05m | 718 |
| 17h:50m | 729 |
| 18h:35m | 741 |
| 19h:20m | 756 |
| 20h:10m | 778 |
| 20h:55m | 800 |
| 21h:40m | 828 |
| 22h:25m | 866 |
| 23h:15m | 934 |
| 24h:00m | 1038 |
| 24h:05m |  |
| 24h:50m |  |
| 25h:40m |  |
| 26h:25m |  |
| 27h:10m |  |
| 27h:55m |  |
| 28h:45m |  |
| 29h:30m |  |
| 30h:15m |  |
| 31h:00m |  |
| 31h:50m |  |
| 32h:35m |  |
| 33h:20m |  |
| 34h:05m |  |
| 34h:55m |  |
| 35h:40m |  |
| 36h:25m |  |
| 37h:10m |  |
| 38h:00m |  |
| 38h:45m |  |
| 39h:30m |  |
| 40h:15m |  |
| 41h:05m |  |
| 41h:50m |  |
| 42h:35m |  |
| 43h:20m |  |
| 44h:10m |  |
| 44h:55m |  |
| 45h:40m |  |
| 46h:25m |  |
| 47h:15m |  |
| 48h:00m |  |
| 48h:05m |  |
| 48h:50m |  |
| 49h:40m |  |
| 50h:25m |  |
| 51h:10m |  |
| 51h:55m |  |
| 52h:45m |  |
| 53h:30m |  |
| 54h:15m |  |
| 55h:00m |  |
| 55h:50m |  |
| 56h:35m |  |
| 57h:20m |  |
| 58h:05m |  |
| 58h:55m |  |
| 59h:40m |  |
| 60h:25m |  |
| 61h:10m |  |
| 62h:00m |  |
| 62h:45m |  |
| 63h:30m |  |
| 64h:15m |  |
| 65h:05m |  |
| 65h:50m |  |
| 66h:35m |  |
| 67h:20m |  |
| 68h:10m |  |
| 68h:55m |  |
| 69h:40m |  |
| 70h:25m |  |
| 71h:15m |  |
| 72h:00m |  |

Staph Aureus Vancomycin 1MIC 2

| Time | SA 1MIC VANCO |
| --- | --- |
| 0h:05m | 583 |
| 0h:50m | 596 |
| 1h:40m | 602 |
| 2h:25m | 604 |
| 3h:10m | 604 |
| 3h:55m | 602 |
| 4h:45m | 603 |
| 5h:30m | 601 |
| 6h:15m | 602 |
| 7h:00m | 600 |
| 7h:50m | 600 |
| 8h:35m | 601 |
| 9h:20m | 602 |
| 10h:05m | 607 |
| 10h:55m | 618 |
| 11h:40m | 630 |
| 12h:25m | 646 |
| 13h:10m | 656 |
| 14h:00m | 667 |
| 14h:45m | 679 |
| 15h:30m | 697 |
| 16h:15m | 714 |
| 17h:05m | 745 |
| 17h:50m | 776 |
| 18h:35m | 815 |
| 19h:20m | 873 |
| 20h:10m | 928 |
| 20h:55m | 988 |
| 21h:40m | 1056 |
| 22h:25m | 1139 |
| 23h:15m | 1291 |
| 24h:00m | 1565 |
| 24h:05m |  |
| 24h:50m |  |
| 25h:40m |  |
| 26h:25m |  |
| 27h:10m |  |
| 27h:55m |  |
| 28h:45m |  |
| 29h:30m |  |
| 30h:15m |  |
| 31h:00m |  |
| 31h:50m |  |
| 32h:35m |  |
| 33h:20m |  |
| 34h:05m |  |
| 34h:55m |  |
| 35h:40m |  |
| 36h:25m |  |
| 37h:10m |  |
| 38h:00m |  |
| 38h:45m |  |
| 39h:30m |  |
| 40h:15m |  |
| 41h:05m |  |
| 41h:50m |  |
| 42h:35m |  |
| 43h:20m |  |
| 44h:10m |  |
| 44h:55m |  |
| 45h:40m |  |
| 46h:25m |  |
| 47h:15m |  |
| 48h:00m |  |
| 48h:05m |  |
| 48h:50m |  |
| 49h:40m |  |
| 50h:25m |  |
| 51h:10m |  |
| 51h:55m |  |
| 52h:45m |  |
| 53h:30m |  |
| 54h:15m |  |
| 55h:00m |  |
| 55h:50m |  |
| 56h:35m |  |
| 57h:20m |  |
| 58h:05m |  |
| 58h:55m |  |
| 59h:40m |  |
| 60h:25m |  |
| 61h:10m |  |
| 62h:00m |  |
| 62h:45m |  |
| 63h:30m |  |
| 64h:15m |  |
| 65h:05m |  |
| 65h:50m |  |
| 66h:35m |  |
| 67h:20m |  |
| 68h:10m |  |
| 68h:55m |  |
| 69h:40m |  |
| 70h:25m |  |
| 71h:15m |  |
| 72h:00m |  |

Staph Aureus Vancomycin 1MIC 3

| Time | SA 1MIC VANCO EXP 4 |
| --- | --- |
| 0h:05m | 686 |
| 0h:50m | 703 |
| 1h:40m | 726 |
| 2h:25m | 728 |
| 3h:10m | 724 |
| 3h:55m | 726 |
| 4h:45m | 724 |
| 5h:30m | 726 |
| 6h:15m | 725 |
| 7h:00m | 726 |
| 7h:50m | 733 |
| 8h:35m | 755 |
| 9h:20m | 816 |
| 10h:05m | 915 |
| 10h:55m | 1063 |
| 11h:40m | 1196 |
| 12h:25m | 1355 |
| 13h:10m | 1725 |
| 14h:00m | 2373 |
| 14h:45m | 3136 |
| 15h:30m | 4373 |
| 16h:15m | 6750 |
| 17h:05m | 15228 |
| 17h:50m | 30985 |
| 18h:35m | 30928 |
| 19h:20m | 30925 |
| 20h:10m | 30925 |
| 20h:55m | 30926 |
| 21h:40m | 30927 |
| 22h:25m | 30930 |
| 23h:15m | 30931 |
| 24h:00m | 30932 |
| 24h:05m |  |
| 24h:50m |  |
| 25h:40m |  |
| 26h:25m |  |
| 27h:10m |  |
| 27h:55m |  |
| 28h:45m |  |
| 29h:30m |  |
| 30h:15m |  |
| 31h:00m |  |
| 31h:50m |  |
| 32h:35m |  |
| 33h:20m |  |
| 34h:05m |  |
| 34h:55m |  |
| 35h:40m |  |
| 36h:25m |  |
| 37h:10m |  |
| 38h:00m |  |
| 38h:45m |  |
| 39h:30m |  |
| 40h:15m |  |
| 41h:05m |  |
| 41h:50m |  |
| 42h:35m |  |
| 43h:20m |  |
| 44h:10m |  |
| 44h:55m |  |
| 45h:40m |  |
| 46h:25m |  |
| 47h:15m |  |
| 48h:00m |  |
| 48h:05m |  |
| 48h:50m |  |
| 49h:40m |  |
| 50h:25m |  |
| 51h:10m |  |
| 51h:55m |  |
| 52h:45m |  |
| 53h:30m |  |
| 54h:15m |  |
| 55h:00m |  |
| 55h:50m |  |
| 56h:35m |  |
| 57h:20m |  |
| 58h:05m |  |
| 58h:55m |  |
| 59h:40m |  |
| 60h:25m |  |
| 61h:10m |  |
| 62h:00m |  |
| 62h:45m |  |
| 63h:30m |  |
| 64h:15m |  |
| 65h:05m |  |
| 65h:50m |  |
| 66h:35m |  |
| 67h:20m |  |
| 68h:10m |  |
| 68h:55m |  |
| 69h:40m |  |
| 70h:25m |  |
| 71h:15m |  |
| 72h:00m |  |

Staph Aureus Vancomycin 1MIC 4

| Time | SA 1MIC VANCO EXP 4 |
| --- | --- |
| 0h:05m | 695 |
| 0h:50m | 711 |
| 1h:40m | 732 |
| 2h:25m | 734 |
| 3h:10m | 729 |
| 3h:55m | 725 |
| 4h:45m | 724 |
| 5h:30m | 723 |
| 6h:15m | 722 |
| 7h:00m | 719 |
| 7h:50m | 722 |
| 8h:35m | 727 |
| 9h:20m | 736 |
| 10h:05m | 750 |
| 10h:55m | 793 |
| 11h:40m | 900 |
| 12h:25m | 1089 |
| 13h:10m | 1345 |
| 14h:00m | 1774 |
| 14h:45m | 2311 |
| 15h:30m | 2922 |
| 16h:15m | 3698 |
| 17h:05m | 5078 |
| 17h:50m | 7833 |
| 18h:35m | 18328 |
| 19h:20m | 30966 |
| 20h:10m | 30916 |
| 20h:55m | 30919 |
| 21h:40m | 30920 |
| 22h:25m | 30922 |
| 23h:15m | 30924 |
| 24h:00m | 30927 |
| 24h:05m |  |
| 24h:50m |  |
| 25h:40m |  |
| 26h:25m |  |
| 27h:10m |  |
| 27h:55m |  |
| 28h:45m |  |
| 29h:30m |  |
| 30h:15m |  |
| 31h:00m |  |
| 31h:50m |  |
| 32h:35m |  |
| 33h:20m |  |
| 34h:05m |  |
| 34h:55m |  |
| 35h:40m |  |
| 36h:25m |  |
| 37h:10m |  |
| 38h:00m |  |
| 38h:45m |  |
| 39h:30m |  |
| 40h:15m |  |
| 41h:05m |  |
| 41h:50m |  |
| 42h:35m |  |
| 43h:20m |  |
| 44h:10m |  |
| 44h:55m |  |
| 45h:40m |  |
| 46h:25m |  |
| 47h:15m |  |
| 48h:00m |  |
| 48h:05m |  |
| 48h:50m |  |
| 49h:40m |  |
| 50h:25m |  |
| 51h:10m |  |
| 51h:55m |  |
| 52h:45m |  |
| 53h:30m |  |
| 54h:15m |  |
| 55h:00m |  |
| 55h:50m |  |
| 56h:35m |  |
| 57h:20m |  |
| 58h:05m |  |
| 58h:55m |  |
| 59h:40m |  |
| 60h:25m |  |
| 61h:10m |  |
| 62h:00m |  |
| 62h:45m |  |
| 63h:30m |  |
| 64h:15m |  |
| 65h:05m |  |
| 65h:50m |  |
| 66h:35m |  |
| 67h:20m |  |
| 68h:10m |  |
| 68h:55m |  |
| 69h:40m |  |
| 70h:25m |  |
| 71h:15m |  |
| 72h:00m |  |

Staph Aureus Vancomycin 1MIC 5

| Time | SA 1MIC VANCO EXP 8 |
| --- | --- |
| 0h:05m | 513 |
| 0h:50m | 527 |
| 1h:40m | 536 |
| 2h:25m | 536 |
| 3h:10m | 533 |
| 3h:55m | 532 |
| 4h:45m | 532 |
| 5h:30m | 533 |
| 6h:15m | 532 |
| 7h:00m | 534 |
| 7h:50m | 532 |
| 8h:35m | 533 |
| 9h:20m | 535 |
| 10h:05m | 538 |
| 10h:55m | 540 |
| 11h:40m | 544 |
| 12h:25m | 549 |
| 13h:10m | 557 |
| 14h:00m | 569 |
| 14h:45m | 578 |
| 15h:30m | 591 |
| 16h:15m | 616 |
| 17h:05m | 668 |
| 17h:50m | 778 |
| 18h:35m | 906 |
| 19h:20m | 1025 |
| 20h:10m | 1172 |
| 20h:55m | 1305 |
| 21h:40m | 1448 |
| 22h:25m | 1592 |
| 23h:15m | 1738 |
| 24h:00m | 1851 |
| 24h:05m |  |
| 24h:50m |  |
| 25h:40m |  |
| 26h:25m |  |
| 27h:10m |  |
| 27h:55m |  |
| 28h:45m |  |
| 29h:30m |  |
| 30h:15m |  |
| 31h:00m |  |
| 31h:50m |  |
| 32h:35m |  |
| 33h:20m |  |
| 34h:05m |  |
| 34h:55m |  |
| 35h:40m |  |
| 36h:25m |  |
| 37h:10m |  |
| 38h:00m |  |
| 38h:45m |  |
| 39h:30m |  |
| 40h:15m |  |
| 41h:05m |  |
| 41h:50m |  |
| 42h:35m |  |
| 43h:20m |  |
| 44h:10m |  |
| 44h:55m |  |
| 45h:40m |  |
| 46h:25m |  |
| 47h:15m |  |
| 48h:00m |  |
| 48h:05m |  |
| 48h:50m |  |
| 49h:40m |  |
| 50h:25m |  |
| 51h:10m |  |
| 51h:55m |  |
| 52h:45m |  |
| 53h:30m |  |
| 54h:15m |  |
| 55h:00m |  |
| 55h:50m |  |
| 56h:35m |  |
| 57h:20m |  |
| 58h:05m |  |
| 58h:55m |  |
| 59h:40m |  |
| 60h:25m |  |
| 61h:10m |  |
| 62h:00m |  |
| 62h:45m |  |
| 63h:30m |  |
| 64h:15m |  |
| 65h:05m |  |
| 65h:50m |  |
| 66h:35m |  |
| 67h:20m |  |
| 68h:10m |  |
| 68h:55m |  |
| 69h:40m |  |
| 70h:25m |  |
| 71h:15m |  |
| 72h:00m |  |

Staph Aureus Vancomycin 1MIC 6

| Time | SA 1MIC VANCO EXP 8 |
| --- | --- |
| 0h:05m | 648 |
| 0h:50m | 635 |
| 1h:40m | 627 |
| 2h:25m | 615 |
| 3h:10m | 610 |
| 3h:55m | 607 |
| 4h:45m | 606 |
| 5h:30m | 603 |
| 6h:15m | 600 |
| 7h:00m | 600 |
| 7h:50m | 602 |
| 8h:35m | 601 |
| 9h:20m | 602 |
| 10h:05m | 604 |
| 10h:55m | 612 |
| 11h:40m | 627 |
| 12h:25m | 645 |
| 13h:10m | 668 |
| 14h:00m | 697 |
| 14h:45m | 717 |
| 15h:30m | 739 |
| 16h:15m | 765 |
| 17h:05m | 795 |
| 17h:50m | 821 |
| 18h:35m | 848 |
| 19h:20m | 880 |
| 20h:10m | 911 |
| 20h:55m | 944 |
| 21h:40m | 997 |
| 22h:25m | 1060 |
| 23h:15m | 1150 |
| 24h:00m | 1217 |
| 24h:05m |  |
| 24h:50m |  |
| 25h:40m |  |
| 26h:25m |  |
| 27h:10m |  |
| 27h:55m |  |
| 28h:45m |  |
| 29h:30m |  |
| 30h:15m |  |
| 31h:00m |  |
| 31h:50m |  |
| 32h:35m |  |
| 33h:20m |  |
| 34h:05m |  |
| 34h:55m |  |
| 35h:40m |  |
| 36h:25m |  |
| 37h:10m |  |
| 38h:00m |  |
| 38h:45m |  |
| 39h:30m |  |
| 40h:15m |  |
| 41h:05m |  |
| 41h:50m |  |
| 42h:35m |  |
| 43h:20m |  |
| 44h:10m |  |
| 44h:55m |  |
| 45h:40m |  |
| 46h:25m |  |
| 47h:15m |  |
| 48h:00m |  |
| 48h:05m |  |
| 48h:50m |  |
| 49h:40m |  |
| 50h:25m |  |
| 51h:10m |  |
| 51h:55m |  |
| 52h:45m |  |
| 53h:30m |  |
| 54h:15m |  |
| 55h:00m |  |
| 55h:50m |  |
| 56h:35m |  |
| 57h:20m |  |
| 58h:05m |  |
| 58h:55m |  |
| 59h:40m |  |
| 60h:25m |  |
| 61h:10m |  |
| 62h:00m |  |
| 62h:45m |  |
| 63h:30m |  |
| 64h:15m |  |
| 65h:05m |  |
| 65h:50m |  |
| 66h:35m |  |
| 67h:20m |  |
| 68h:10m |  |
| 68h:55m |  |
| 69h:40m |  |
| 70h:25m |  |
| 71h:15m |  |
| 72h:00m |  |

Staph Aureus Vancomycin 1MIC 7

| Time | SA 1MIC VANCO EXP W |
| --- | --- |
| 0h:05m | 625 |
| 0h:50m | 616 |
| 1h:40m | 616 |
| 2h:25m | 617 |
| 3h:10m | 620 |
| 3h:55m | 621 |
| 4h:45m | 622 |
| 5h:30m | 622 |
| 6h:15m | 625 |
| 7h:00m | 622 |
| 7h:50m | 624 |
| 8h:35m | 625 |
| 9h:20m | 626 |
| 10h:05m | 627 |
| 10h:55m | 626 |
| 11h:40m | 627 |
| 12h:25m | 627 |
| 13h:10m | 626 |
| 14h:00m | 627 |
| 14h:45m | 628 |
| 15h:30m | 629 |
| 16h:15m | 628 |
| 17h:05m | 630 |
| 17h:50m | 636 |
| 18h:35m | 638 |
| 19h:20m | 641 |
| 20h:10m | 643 |
| 20h:55m | 648 |
| 21h:40m | 653 |
| 22h:25m | 662 |
| 23h:15m | 683 |
| 24h:00m | 1029 |
| 24h:05m | 1237 |
| 24h:50m | 1282 |
| 25h:40m | 1330 |
| 26h:25m | 1308 |
| 27h:10m | 1284 |
| 27h:55m | 1257 |
| 28h:45m | 1234 |
| 29h:30m | 1216 |
| 30h:15m | 1216 |
| 31h:00m | 1231 |
| 31h:50m | 1252 |
| 32h:35m | 1276 |
| 33h:20m | 1299 |
| 34h:05m | 1317 |
| 34h:55m | 1355 |
| 35h:40m | 1460 |
| 36h:25m | 1684 |
| 37h:10m | 1964 |
| 38h:00m | 2410 |
| 38h:45m | 3084 |
| 39h:30m | 4065 |
| 40h:15m | 5587 |
| 41h:05m | 7830 |
| 41h:50m | 10383 |
| 42h:35m | 13032 |
| 43h:20m | 15585 |
| 44h:10m | 19511 |
| 44h:55m | 25222 |
| 45h:40m | 30894 |
| 46h:25m | 30967 |
| 47h:15m | 30940 |
| 48h:00m | 30937 |
| 48h:05m |  |
| 48h:50m |  |
| 49h:40m |  |
| 50h:25m |  |
| 51h:10m |  |
| 51h:55m |  |
| 52h:45m |  |
| 53h:30m |  |
| 54h:15m |  |
| 55h:00m |  |
| 55h:50m |  |
| 56h:35m |  |
| 57h:20m |  |
| 58h:05m |  |
| 58h:55m |  |
| 59h:40m |  |
| 60h:25m |  |
| 61h:10m |  |
| 62h:00m |  |
| 62h:45m |  |
| 63h:30m |  |
| 64h:15m |  |
| 65h:05m |  |
| 65h:50m |  |
| 66h:35m |  |
| 67h:20m |  |
| 68h:10m |  |
| 68h:55m |  |
| 69h:40m |  |
| 70h:25m |  |
| 71h:15m |  |
| 72h:00m |  |

Staph Aureus Vancomycin 1MIC 8

| Time | SA 1MIC VANCO EXP W |
| --- | --- |
| 0h:05m | 497 |
| 0h:50m | 492 |
| 1h:40m | 497 |
| 2h:25m | 501 |
| 3h:10m | 502 |
| 3h:55m | 503 |
| 4h:45m | 505 |
| 5h:30m | 505 |
| 6h:15m | 509 |
| 7h:00m | 510 |
| 7h:50m | 510 |
| 8h:35m | 512 |
| 9h:20m | 512 |
| 10h:05m | 514 |
| 10h:55m | 513 |
| 11h:40m | 516 |
| 12h:25m | 518 |
| 13h:10m | 518 |
| 14h:00m | 519 |
| 14h:45m | 519 |
| 15h:30m | 521 |
| 16h:15m | 524 |
| 17h:05m | 523 |
| 17h:50m | 524 |
| 18h:35m | 525 |
| 19h:20m | 530 |
| 20h:10m | 529 |
| 20h:55m | 530 |
| 21h:40m | 530 |
| 22h:25m | 531 |
| 23h:15m | 531 |
| 24h:00m | 513 |
| 24h:05m | 356 |
| 24h:50m | 361 |
| 25h:40m | 362 |
| 26h:25m | 359 |
| 27h:10m | 366 |
| 27h:55m | 362 |
| 28h:45m | 365 |
| 29h:30m | 365 |
| 30h:15m | 365 |
| 31h:00m | 365 |
| 31h:50m | 368 |
| 32h:35m | 368 |
| 33h:20m | 366 |
| 34h:05m | 366 |
| 34h:55m | 367 |
| 35h:40m | 371 |
| 36h:25m | 366 |
| 37h:10m | 369 |
| 38h:00m | 371 |
| 38h:45m | 369 |
| 39h:30m | 374 |
| 40h:15m | 377 |
| 41h:05m | 394 |
| 41h:50m | 420 |
| 42h:35m | 467 |
| 43h:20m | 523 |
| 44h:10m | 610 |
| 44h:55m | 695 |
| 45h:40m | 833 |
| 46h:25m | 1029 |
| 47h:15m | 1583 |
| 48h:00m | 2184 |
| 48h:05m |  |
| 48h:50m |  |
| 49h:40m |  |
| 50h:25m |  |
| 51h:10m |  |
| 51h:55m |  |
| 52h:45m |  |
| 53h:30m |  |
| 54h:15m |  |
| 55h:00m |  |
| 55h:50m |  |
| 56h:35m |  |
| 57h:20m |  |
| 58h:05m |  |
| 58h:55m |  |
| 59h:40m |  |
| 60h:25m |  |
| 61h:10m |  |
| 62h:00m |  |
| 62h:45m |  |
| 63h:30m |  |
| 64h:15m |  |
| 65h:05m |  |
| 65h:50m |  |
| 66h:35m |  |
| 67h:20m |  |
| 68h:10m |  |
| 68h:55m |  |
| 69h:40m |  |
| 70h:25m |  |
| 71h:15m |  |
| 72h:00m |  |

Staph Aureus Vancomycin 1MIC 9

| Time | 1 MIC |
| --- | --- |
| 00:05 | 379 |
| 02:25 | 406 |
| 04:45 | 412 |
| 07:05 | 412 |
| 09:20 | 415 |
| 11:40 | 417 |
| 14:00 | 426 |
| 16:20 | 450 |
| 18:40 | 500 |
| 21:00 | 631 |
| 23:15 | 1185 |
| 25:35 | 2122 |
| 27:55 | 3228 |
| 30:15 | 5631 |
| 32:35 | 9977 |
| 34:55 | 15777 |
| 37:10 | 30909 |
| 39:30 | 30929 |
| 41:50 | 30927 |
| 44:10 | 30926 |
| 46:30 | 30927 |
| 48:50 | 30927 |
| 51:05 | 30929 |
| 53:25 | 30929 |
| 55:45 | 30933 |
| 58:05 | 30929 |
| 60:25 | 30936 |
| 62:45 | 30938 |
| 65:00 | 30939 |
| 67:20 | 30940 |
| 69:40 | 30944 |
| 72:00 | 30937 |

Staph Aureus Vancomycin 1MIC 10

| Time | 1 MIC |
| --- | --- |
| 00:05 | 585 |
| 02:25 | 602 |
| 04:45 | 599 |
| 07:05 | 596 |
| 09:20 | 593 |
| 11:40 | 595 |
| 14:00 | 589 |
| 16:20 | 592 |
| 18:40 | 593 |
| 21:00 | 596 |
| 23:15 | 596 |
| 25:35 | 599 |
| 27:55 | 599 |
| 30:15 | 600 |
| 32:35 | 602 |
| 34:55 | 599 |
| 37:10 | 600 |
| 39:30 | 603 |
| 41:50 | 605 |
| 44:10 | 646 |
| 46:30 | 821 |
| 48:50 | 2564 |
| 51:05 | 2373 |
| 53:25 | 2413 |
| 55:45 | 2563 |
| 58:05 | 3123 |
| 60:25 | 3545 |
| 62:45 | 3851 |
| 65:00 | 8319 |
| 67:20 | 14017 |
| 69:40 | 24106 |
| 72:00 | 30925 |

Staph Aureus Vancomycin 4MIC 1

| Time | SA 4MIC VANCO |
| --- | --- |
| 0h:05m | 599 |
| 0h:50m | 603 |
| 1h:40m | 609 |
| 2h:25m | 605 |
| 3h:10m | 603 |
| 3h:55m | 605 |
| 4h:45m | 605 |
| 5h:30m | 604 |
| 6h:15m | 601 |
| 7h:00m | 602 |
| 7h:50m | 600 |
| 8h:35m | 601 |
| 9h:20m | 602 |
| 10h:05m | 607 |
| 10h:55m | 621 |
| 11h:40m | 648 |
| 12h:25m | 679 |
| 13h:10m | 704 |
| 14h:00m | 728 |
| 14h:45m | 750 |
| 15h:30m | 773 |
| 16h:15m | 796 |
| 17h:05m | 832 |
| 17h:50m | 871 |
| 18h:35m | 914 |
| 19h:20m | 970 |
| 20h:10m | 1038 |
| 20h:55m | 1116 |
| 21h:40m | 1198 |
| 22h:25m | 1280 |
| 23h:15m | 1375 |
| 24h:00m | 1480 |
| 24h:05m | 2982 |
| 24h:50m | 3358 |
| 25h:40m | 3503 |
| 26h:25m | 3431 |
| 27h:10m | 3348 |
| 27h:55m | 3246 |
| 28h:45m | 3158 |
| 29h:30m | 3088 |
| 30h:15m | 3021 |
| 31h:00m | 2971 |
| 31h:50m | 2926 |
| 32h:35m | 2896 |
| 33h:20m | 2866 |
| 34h:05m | 2847 |
| 34h:55m | 2832 |
| 35h:40m | 2823 |
| 36h:25m | 2825 |
| 37h:10m | 2823 |
| 38h:00m | 2824 |
| 38h:45m | 2829 |
| 39h:30m | 2840 |
| 40h:15m | 2859 |
| 41h:05m | 2879 |
| 41h:50m | 2937 |
| 42h:35m | 2980 |
| 43h:20m | 3037 |
| 44h:10m | 3085 |
| 44h:55m | 3138 |
| 45h:40m | 3200 |
| 46h:25m | 3263 |
| 47h:15m | 3351 |
| 48h:00m | 3465 |
| 48h:05m |  |
| 48h:50m |  |
| 49h:40m |  |
| 50h:25m |  |
| 51h:10m |  |
| 51h:55m |  |
| 52h:45m |  |
| 53h:30m |  |
| 54h:15m |  |
| 55h:00m |  |
| 55h:50m |  |
| 56h:35m |  |
| 57h:20m |  |
| 58h:05m |  |
| 58h:55m |  |
| 59h:40m |  |
| 60h:25m |  |
| 61h:10m |  |
| 62h:00m |  |
| 62h:45m |  |
| 63h:30m |  |
| 64h:15m |  |
| 65h:05m |  |
| 65h:50m |  |
| 66h:35m |  |
| 67h:20m |  |
| 68h:10m |  |
| 68h:55m |  |
| 69h:40m |  |
| 70h:25m |  |
| 71h:15m |  |
| 72h:00m |  |

Staph Aureus Vancomycin 4MIC 2

| Time | SA 4MIC VANCO EXP 4 |
| --- | --- |
| 0h:05m | 633 |
| 0h:50m | 652 |
| 1h:40m | 659 |
| 2h:25m | 658 |
| 3h:10m | 653 |
| 3h:55m | 654 |
| 4h:45m | 652 |
| 5h:30m | 649 |
| 6h:15m | 652 |
| 7h:00m | 650 |
| 7h:50m | 652 |
| 8h:35m | 661 |
| 9h:20m | 673 |
| 10h:05m | 692 |
| 10h:55m | 722 |
| 11h:40m | 753 |
| 12h:25m | 787 |
| 13h:10m | 817 |
| 14h:00m | 853 |
| 14h:45m | 886 |
| 15h:30m | 928 |
| 16h:15m | 1007 |
| 17h:05m | 1134 |
| 17h:50m | 1327 |
| 18h:35m | 1474 |
| 19h:20m | 1660 |
| 20h:10m | 2000 |
| 20h:55m | 2479 |
| 21h:40m | 3202 |
| 22h:25m | 4018 |
| 23h:15m | 4904 |
| 24h:00m | 5697 |
| 24h:05m |  |
| 24h:50m |  |
| 25h:40m |  |
| 26h:25m |  |
| 27h:10m |  |
| 27h:55m |  |
| 28h:45m |  |
| 29h:30m |  |
| 30h:15m |  |
| 31h:00m |  |
| 31h:50m |  |
| 32h:35m |  |
| 33h:20m |  |
| 34h:05m |  |
| 34h:55m |  |
| 35h:40m |  |
| 36h:25m |  |
| 37h:10m |  |
| 38h:00m |  |
| 38h:45m |  |
| 39h:30m |  |
| 40h:15m |  |
| 41h:05m |  |
| 41h:50m |  |
| 42h:35m |  |
| 43h:20m |  |
| 44h:10m |  |
| 44h:55m |  |
| 45h:40m |  |
| 46h:25m |  |
| 47h:15m |  |
| 48h:00m |  |
| 48h:05m |  |
| 48h:50m |  |
| 49h:40m |  |
| 50h:25m |  |
| 51h:10m |  |
| 51h:55m |  |
| 52h:45m |  |
| 53h:30m |  |
| 54h:15m |  |
| 55h:00m |  |
| 55h:50m |  |
| 56h:35m |  |
| 57h:20m |  |
| 58h:05m |  |
| 58h:55m |  |
| 59h:40m |  |
| 60h:25m |  |
| 61h:10m |  |
| 62h:00m |  |
| 62h:45m |  |
| 63h:30m |  |
| 64h:15m |  |
| 65h:05m |  |
| 65h:50m |  |
| 66h:35m |  |
| 67h:20m |  |
| 68h:10m |  |
| 68h:55m |  |
| 69h:40m |  |
| 70h:25m |  |
| 71h:15m |  |
| 72h:00m |  |

Staph Aureus Vancomycin 4MIC 3

| Time | SA 4MIC VANCO EXP 8 |
| --- | --- |
| 0h:05m | 513 |
| 0h:50m | 516 |
| 1h:40m | 520 |
| 2h:25m | 519 |
| 3h:10m | 519 |
| 3h:55m | 519 |
| 4h:45m | 519 |
| 5h:30m | 519 |
| 6h:15m | 516 |
| 7h:00m | 517 |
| 7h:50m | 521 |
| 8h:35m | 524 |
| 9h:20m | 527 |
| 10h:05m | 534 |
| 10h:55m | 542 |
| 11h:40m | 548 |
| 12h:25m | 555 |
| 13h:10m | 561 |
| 14h:00m | 567 |
| 14h:45m | 574 |
| 15h:30m | 589 |
| 16h:15m | 612 |
| 17h:05m | 652 |
| 17h:50m | 694 |
| 18h:35m | 739 |
| 19h:20m | 802 |
| 20h:10m | 861 |
| 20h:55m | 914 |
| 21h:40m | 972 |
| 22h:25m | 1039 |
| 23h:15m | 1119 |
| 24h:00m | 1188 |
| 24h:05m |  |
| 24h:50m |  |
| 25h:40m |  |
| 26h:25m |  |
| 27h:10m |  |
| 27h:55m |  |
| 28h:45m |  |
| 29h:30m |  |
| 30h:15m |  |
| 31h:00m |  |
| 31h:50m |  |
| 32h:35m |  |
| 33h:20m |  |
| 34h:05m |  |
| 34h:55m |  |
| 35h:40m |  |
| 36h:25m |  |
| 37h:10m |  |
| 38h:00m |  |
| 38h:45m |  |
| 39h:30m |  |
| 40h:15m |  |
| 41h:05m |  |
| 41h:50m |  |
| 42h:35m |  |
| 43h:20m |  |
| 44h:10m |  |
| 44h:55m |  |
| 45h:40m |  |
| 46h:25m |  |
| 47h:15m |  |
| 48h:00m |  |
| 48h:05m |  |
| 48h:50m |  |
| 49h:40m |  |
| 50h:25m |  |
| 51h:10m |  |
| 51h:55m |  |
| 52h:45m |  |
| 53h:30m |  |
| 54h:15m |  |
| 55h:00m |  |
| 55h:50m |  |
| 56h:35m |  |
| 57h:20m |  |
| 58h:05m |  |
| 58h:55m |  |
| 59h:40m |  |
| 60h:25m |  |
| 61h:10m |  |
| 62h:00m |  |
| 62h:45m |  |
| 63h:30m |  |
| 64h:15m |  |
| 65h:05m |  |
| 65h:50m |  |
| 66h:35m |  |
| 67h:20m |  |
| 68h:10m |  |
| 68h:55m |  |
| 69h:40m |  |
| 70h:25m |  |
| 71h:15m |  |
| 72h:00m |  |

Staph Aureus Vancomycin 4MIC 4

| Time | SA 4MIC VANCO EXP 8 |
| --- | --- |
| 0h:05m | 497 |
| 0h:50m | 503 |
| 1h:40m | 507 |
| 2h:25m | 507 |
| 3h:10m | 508 |
| 3h:55m | 509 |
| 4h:45m | 509 |
| 5h:30m | 506 |
| 6h:15m | 507 |
| 7h:00m | 508 |
| 7h:50m | 508 |
| 8h:35m | 512 |
| 9h:20m | 516 |
| 10h:05m | 522 |
| 10h:55m | 530 |
| 11h:40m | 538 |
| 12h:25m | 550 |
| 13h:10m | 569 |
| 14h:00m | 591 |
| 14h:45m | 618 |
| 15h:30m | 653 |
| 16h:15m | 694 |
| 17h:05m | 758 |
| 17h:50m | 823 |
| 18h:35m | 885 |
| 19h:20m | 954 |
| 20h:10m | 1030 |
| 20h:55m | 1097 |
| 21h:40m | 1174 |
| 22h:25m | 1244 |
| 23h:15m | 1346 |
| 24h:00m | 1430 |
| 24h:05m |  |
| 24h:50m |  |
| 25h:40m |  |
| 26h:25m |  |
| 27h:10m |  |
| 27h:55m |  |
| 28h:45m |  |
| 29h:30m |  |
| 30h:15m |  |
| 31h:00m |  |
| 31h:50m |  |
| 32h:35m |  |
| 33h:20m |  |
| 34h:05m |  |
| 34h:55m |  |
| 35h:40m |  |
| 36h:25m |  |
| 37h:10m |  |
| 38h:00m |  |
| 38h:45m |  |
| 39h:30m |  |
| 40h:15m |  |
| 41h:05m |  |
| 41h:50m |  |
| 42h:35m |  |
| 43h:20m |  |
| 44h:10m |  |
| 44h:55m |  |
| 45h:40m |  |
| 46h:25m |  |
| 47h:15m |  |
| 48h:00m |  |
| 48h:05m |  |
| 48h:50m |  |
| 49h:40m |  |
| 50h:25m |  |
| 51h:10m |  |
| 51h:55m |  |
| 52h:45m |  |
| 53h:30m |  |
| 54h:15m |  |
| 55h:00m |  |
| 55h:50m |  |
| 56h:35m |  |
| 57h:20m |  |
| 58h:05m |  |
| 58h:55m |  |
| 59h:40m |  |
| 60h:25m |  |
| 61h:10m |  |
| 62h:00m |  |
| 62h:45m |  |
| 63h:30m |  |
| 64h:15m |  |
| 65h:05m |  |
| 65h:50m |  |
| 66h:35m |  |
| 67h:20m |  |
| 68h:10m |  |
| 68h:55m |  |
| 69h:40m |  |
| 70h:25m |  |
| 71h:15m |  |
| 72h:00m |  |

Staph Aureus Vancomycin 4MIC 5

| Time | SA 4MIC VANCO EXP W |
| --- | --- |
| 0h:05m | 380 |
| 0h:50m | 372 |
| 1h:40m | 376 |
| 2h:25m | 379 |
| 3h:10m | 380 |
| 3h:55m | 382 |
| 4h:45m | 385 |
| 5h:30m | 386 |
| 6h:15m | 387 |
| 7h:00m | 390 |
| 7h:50m | 393 |
| 8h:35m | 396 |
| 9h:20m | 398 |
| 10h:05m | 400 |
| 10h:55m | 400 |
| 11h:40m | 403 |
| 12h:25m | 405 |
| 13h:10m | 406 |
| 14h:00m | 406 |
| 14h:45m | 408 |
| 15h:30m | 411 |
| 16h:15m | 412 |
| 17h:05m | 413 |
| 17h:50m | 416 |
| 18h:35m | 416 |
| 19h:20m | 417 |
| 20h:10m | 418 |
| 20h:55m | 421 |
| 21h:40m | 421 |
| 22h:25m | 422 |
| 23h:15m | 423 |
| 24h:00m | 429 |
| 24h:05m | 687 |
| 24h:50m | 719 |
| 25h:40m | 734 |
| 26h:25m | 731 |
| 27h:10m | 728 |
| 27h:55m | 724 |
| 28h:45m | 714 |
| 29h:30m | 713 |
| 30h:15m | 709 |
| 31h:00m | 709 |
| 31h:50m | 708 |
| 32h:35m | 707 |
| 33h:20m | 707 |
| 34h:05m | 708 |
| 34h:55m | 705 |
| 35h:40m | 707 |
| 36h:25m | 706 |
| 37h:10m | 713 |
| 38h:00m | 730 |
| 38h:45m | 753 |
| 39h:30m | 775 |
| 40h:15m | 804 |
| 41h:05m | 853 |
| 41h:50m | 908 |
| 42h:35m | 1022 |
| 43h:20m | 1139 |
| 44h:10m | 1261 |
| 44h:55m | 1431 |
| 45h:40m | 1637 |
| 46h:25m | 1830 |
| 47h:15m | 2179 |
| 48h:00m | 2664 |
| 48h:05m |  |
| 48h:50m |  |
| 49h:40m |  |
| 50h:25m |  |
| 51h:10m |  |
| 51h:55m |  |
| 52h:45m |  |
| 53h:30m |  |
| 54h:15m |  |
| 55h:00m |  |
| 55h:50m |  |
| 56h:35m |  |
| 57h:20m |  |
| 58h:05m |  |
| 58h:55m |  |
| 59h:40m |  |
| 60h:25m |  |
| 61h:10m |  |
| 62h:00m |  |
| 62h:45m |  |
| 63h:30m |  |
| 64h:15m |  |
| 65h:05m |  |
| 65h:50m |  |
| 66h:35m |  |
| 67h:20m |  |
| 68h:10m |  |
| 68h:55m |  |
| 69h:40m |  |
| 70h:25m |  |
| 71h:15m |  |
| 72h:00m |  |

Staph Aureus Vancomycin 4MIC 6

| Time | SA 4MIC VANCO EXP W |
| --- | --- |
| 0h:05m | 374 |
| 0h:50m | 369 |
| 1h:40m | 370 |
| 2h:25m | 371 |
| 3h:10m | 373 |
| 3h:55m | 373 |
| 4h:45m | 374 |
| 5h:30m | 375 |
| 6h:15m | 374 |
| 7h:00m | 377 |
| 7h:50m | 377 |
| 8h:35m | 378 |
| 9h:20m | 380 |
| 10h:05m | 381 |
| 10h:55m | 381 |
| 11h:40m | 383 |
| 12h:25m | 383 |
| 13h:10m | 383 |
| 14h:00m | 383 |
| 14h:45m | 385 |
| 15h:30m | 385 |
| 16h:15m | 385 |
| 17h:05m | 386 |
| 17h:50m | 387 |
| 18h:35m | 387 |
| 19h:20m | 388 |
| 20h:10m | 389 |
| 20h:55m | 393 |
| 21h:40m | 391 |
| 22h:25m | 394 |
| 23h:15m | 397 |
| 24h:00m | 427 |
| 24h:05m | 825 |
| 24h:50m | 894 |
| 25h:40m | 952 |
| 26h:25m | 946 |
| 27h:10m | 928 |
| 27h:55m | 906 |
| 28h:45m | 891 |
| 29h:30m | 878 |
| 30h:15m | 868 |
| 31h:00m | 855 |
| 31h:50m | 843 |
| 32h:35m | 843 |
| 33h:20m | 838 |
| 34h:05m | 832 |
| 34h:55m | 829 |
| 35h:40m | 827 |
| 36h:25m | 827 |
| 37h:10m | 827 |
| 38h:00m | 831 |
| 38h:45m | 853 |
| 39h:30m | 897 |
| 40h:15m | 954 |
| 41h:05m | 1022 |
| 41h:50m | 1073 |
| 42h:35m | 1131 |
| 43h:20m | 1184 |
| 44h:10m | 1308 |
| 44h:55m | 1397 |
| 45h:40m | 1557 |
| 46h:25m | 1874 |
| 47h:15m | 2714 |
| 48h:00m | 3134 |
| 48h:05m |  |
| 48h:50m |  |
| 49h:40m |  |
| 50h:25m |  |
| 51h:10m |  |
| 51h:55m |  |
| 52h:45m |  |
| 53h:30m |  |
| 54h:15m |  |
| 55h:00m |  |
| 55h:50m |  |
| 56h:35m |  |
| 57h:20m |  |
| 58h:05m |  |
| 58h:55m |  |
| 59h:40m |  |
| 60h:25m |  |
| 61h:10m |  |
| 62h:00m |  |
| 62h:45m |  |
| 63h:30m |  |
| 64h:15m |  |
| 65h:05m |  |
| 65h:50m |  |
| 66h:35m |  |
| 67h:20m |  |
| 68h:10m |  |
| 68h:55m |  |
| 69h:40m |  |
| 70h:25m |  |
| 71h:15m |  |
| 72h:00m |  |

Staph Aureus Vancomycin 4MIC 7

| Time | SA 4MIC VANCO EXP W |
| --- | --- |
| 00:05 | 669 |
| 02:25 | 707 |
| 04:45 | 712 |
| 07:05 | 712 |
| 09:20 | 725 |
| 11:40 | 740 |
| 14:00 | 746 |
| 16:20 | 790 |
| 18:40 | 838 |
| 21:00 | 866 |
| 23:15 | 899 |
| 25:35 | 923 |
| 27:55 | 950 |
| 30:15 | 968 |
| 32:35 | 976 |
| 34:55 | 997 |
| 37:10 | 994 |
| 39:30 | 1003 |
| 41:50 | 1008 |
| 44:10 | 1012 |
| 46:30 | 1039 |
| 48:50 | 1049 |
| 51:05 | 1067 |
| 53:25 | 1089 |
| 55:45 | 1090 |
| 58:05 | 1122 |
| 60:25 | 1126 |
| 62:45 | 1147 |
| 65:00 | 1162 |
| 67:20 | 1191 |
| 69:40 | 1225 |
| 72:00 | 1270 |

Staph Aureus Vancomycin 4MIC 8

| Time | SA 4MIC VANCO EXP W |
| --- | --- |
| 00:05 | 365 |
| 02:25 | 384 |
| 04:45 | 388 |
| 07:05 | 382 |
| 09:20 | 382 |
| 11:40 | 382 |
| 14:00 | 381 |
| 16:20 | 380 |
| 18:40 | 380 |
| 21:00 | 379 |
| 23:15 | 380 |
| 25:35 | 380 |
| 27:55 | 380 |
| 30:15 | 378 |
| 32:35 | 380 |
| 34:55 | 380 |
| 37:10 | 380 |
| 39:30 | 380 |
| 41:50 | 379 |
| 44:10 | 382 |
| 46:30 | 382 |
| 48:50 | 382 |
| 51:05 | 382 |
| 53:25 | 382 |
| 55:45 | 383 |
| 58:05 | 383 |
| 60:25 | 384 |
| 62:45 | 396 |
| 65:00 | 411 |
| 67:20 | 494 |
| 69:40 | 693 |
| 72:00 | 1082 |

Staph Aureus Vancomycin 1000MIC 1

| Time | SA 1000MIC VANCO |
| --- | --- |
| 0h:05m | 661 |
| 0h:50m | 658 |
| 1h:40m | 660 |
| 2h:25m | 662 |
| 3h:10m | 663 |
| 3h:55m | 666 |
| 4h:45m | 664 |
| 5h:30m | 664 |
| 6h:15m | 668 |
| 7h:00m | 668 |
| 7h:50m | 668 |
| 8h:35m | 669 |
| 9h:20m | 669 |
| 10h:05m | 670 |
| 10h:55m | 668 |
| 11h:40m | 669 |
| 12h:25m | 671 |
| 13h:10m | 671 |
| 14h:00m | 670 |
| 14h:45m | 672 |
| 15h:30m | 671 |
| 16h:15m | 674 |
| 17h:05m | 676 |
| 17h:50m | 673 |
| 18h:35m | 674 |
| 19h:20m | 676 |
| 20h:10m | 677 |
| 20h:55m | 677 |
| 21h:40m | 677 |
| 22h:25m | 680 |
| 23h:15m | 678 |
| 24h:00m | 678 |
| 24h:05m | 744 |
| 24h:50m | 744 |
| 25h:40m | 742 |
| 26h:25m | 742 |
| 27h:10m | 740 |
| 27h:55m | 740 |
| 28h:45m | 743 |
| 29h:30m | 740 |
| 30h:15m | 741 |
| 31h:00m | 741 |
| 31h:50m | 740 |
| 32h:35m | 739 |
| 33h:20m | 742 |
| 34h:05m | 740 |
| 34h:55m | 739 |
| 35h:40m | 740 |
| 36h:25m | 743 |
| 37h:10m | 742 |
| 38h:00m | 741 |
| 38h:45m | 740 |
| 39h:30m | 742 |
| 40h:15m | 742 |
| 41h:05m | 739 |
| 41h:50m | 737 |
| 42h:35m | 735 |
| 43h:20m | 736 |
| 44h:10m | 740 |
| 44h:55m | 739 |
| 45h:40m | 739 |
| 46h:25m | 741 |
| 47h:15m | 743 |
| 48h:00m | 738 |
| 48h:05m | 782 |
| 48h:50m | 775 |
| 49h:40m | 774 |
| 50h:25m | 775 |
| 51h:10m | 777 |
| 51h:55m | 775 |
| 52h:45m | 778 |
| 53h:30m | 778 |
| 54h:15m | 776 |
| 55h:00m | 777 |
| 55h:50m | 777 |
| 56h:35m | 775 |
| 57h:20m | 776 |
| 58h:05m | 778 |
| 58h:55m | 778 |
| 59h:40m | 778 |
| 60h:25m | 779 |
| 61h:10m | 775 |
| 62h:00m | 775 |
| 62h:45m | 774 |
| 63h:30m | 776 |
| 64h:15m | 776 |
| 65h:05m | 777 |
| 65h:50m | 775 |
| 66h:35m | 777 |
| 67h:20m | 775 |
| 68h:10m | 778 |
| 68h:55m | 776 |
| 69h:40m | 779 |
| 70h:25m | 778 |
| 71h:15m | 778 |
| 72h:00m | 776 |

Staph Aureus Vancomycin 1000MIC 2

| Time | SA 1000MIC VANCO |
| --- | --- |
| 0h:05m | 824 |
| 0h:50m | 825 |
| 1h:40m | 831 |
| 2h:25m | 832 |
| 3h:10m | 836 |
| 3h:55m | 839 |
| 4h:45m | 842 |
| 5h:30m | 841 |
| 6h:15m | 844 |
| 7h:00m | 846 |
| 7h:50m | 845 |
| 8h:35m | 850 |
| 9h:20m | 849 |
| 10h:05m | 854 |
| 10h:55m | 858 |
| 11h:40m | 860 |
| 12h:25m | 864 |
| 13h:10m | 870 |
| 14h:00m | 876 |
| 14h:45m | 876 |
| 15h:30m | 880 |
| 16h:15m | 881 |
| 17h:05m | 887 |
| 17h:50m | 888 |
| 18h:35m | 891 |
| 19h:20m | 897 |
| 20h:10m | 903 |
| 20h:55m | 905 |
| 21h:40m | 912 |
| 22h:25m | 914 |
| 23h:15m | 919 |
| 24h:00m | 923 |
| 24h:05m | 1371 |
| 24h:50m | 1406 |
| 25h:40m | 1436 |
| 26h:25m | 1442 |
| 27h:10m | 1445 |
| 27h:55m | 1451 |
| 28h:45m | 1451 |
| 29h:30m | 1449 |
| 30h:15m | 1453 |
| 31h:00m | 1459 |
| 31h:50m | 1465 |
| 32h:35m | 1466 |
| 33h:20m | 1469 |
| 34h:05m | 1474 |
| 34h:55m | 1481 |
| 35h:40m | 1485 |
| 36h:25m | 1494 |
| 37h:10m | 1495 |
| 38h:00m | 1498 |
| 38h:45m | 1500 |
| 39h:30m | 1507 |
| 40h:15m | 1511 |
| 41h:05m | 1509 |
| 41h:50m | 1506 |
| 42h:35m | 1513 |
| 43h:20m | 1518 |
| 44h:10m | 1519 |
| 44h:55m | 1521 |
| 45h:40m | 1524 |
| 46h:25m | 1526 |
| 47h:15m | 1525 |
| 48h:00m | 1526 |
| 48h:05m | 1377 |
| 48h:50m | 1400 |
| 49h:40m | 1414 |
| 50h:25m | 1421 |
| 51h:10m | 1426 |
| 51h:55m | 1426 |
| 52h:45m | 1432 |
| 53h:30m | 1436 |
| 54h:15m | 1433 |
| 55h:00m | 1441 |
| 55h:50m | 1444 |
| 56h:35m | 1438 |
| 57h:20m | 1444 |
| 58h:05m | 1446 |
| 58h:55m | 1450 |
| 59h:40m | 1449 |
| 60h:25m | 1449 |
| 61h:10m | 1448 |
| 62h:00m | 1454 |
| 62h:45m | 1456 |
| 63h:30m | 1455 |
| 64h:15m | 1455 |
| 65h:05m | 1460 |
| 65h:50m | 1460 |
| 66h:35m | 1461 |
| 67h:20m | 1465 |
| 68h:10m | 1459 |
| 68h:55m | 1462 |
| 69h:40m | 1458 |
| 70h:25m | 1464 |
| 71h:15m | 1463 |
| 72h:00m | 1466 |

Staph Aureus Vancomycin 1000MIC 3

| Time | SA 1000MIC VANCO EXP 4 |
| --- | --- |
| 0h:05m | 1496 |
| 0h:50m | 1522 |
| 1h:40m | 1568 |
| 2h:25m | 1590 |
| 3h:10m | 1601 |
| 3h:55m | 1619 |
| 4h:45m | 1633 |
| 5h:30m | 1644 |
| 6h:15m | 1648 |
| 7h:00m | 1665 |
| 7h:50m | 1671 |
| 8h:35m | 1675 |
| 9h:20m | 1687 |
| 10h:05m | 1700 |
| 10h:55m | 1710 |
| 11h:40m | 1709 |
| 12h:25m | 1712 |
| 13h:10m | 1721 |
| 14h:00m | 1729 |
| 14h:45m | 1732 |
| 15h:30m | 1738 |
| 16h:15m | 1743 |
| 17h:05m | 1747 |
| 17h:50m | 1747 |
| 18h:35m | 1755 |
| 19h:20m | 1756 |
| 20h:10m | 1755 |
| 20h:55m | 1764 |
| 21h:40m | 1775 |
| 22h:25m | 1765 |
| 23h:15m | 1774 |
| 24h:00m | 1762 |
| 24h:05m | 1691 |
| 24h:50m | 1703 |
| 25h:40m | 1699 |
| 26h:25m | 1702 |
| 27h:10m | 1698 |
| 27h:55m | 1704 |
| 28h:45m | 1704 |
| 29h:30m | 1706 |
| 30h:15m | 1708 |
| 31h:00m | 1709 |
| 31h:50m | 1707 |
| 32h:35m | 1701 |
| 33h:20m | 1713 |
| 34h:05m | 1708 |
| 34h:55m | 1709 |
| 35h:40m | 1710 |
| 36h:25m | 1712 |
| 37h:10m | 1720 |
| 38h:00m | 1721 |
| 38h:45m | 1715 |
| 39h:30m | 1726 |
| 40h:15m | 1727 |
| 41h:05m | 1721 |
| 41h:50m | 1726 |
| 42h:35m | 1729 |
| 43h:20m | 1729 |
| 44h:10m | 1722 |
| 44h:55m | 1712 |
| 45h:40m | 1713 |
| 46h:25m | 1712 |
| 47h:15m | 1715 |
| 48h:00m | 1714 |
| 48h:05m | 1447 |
| 48h:50m | 1446 |
| 49h:40m | 1442 |
| 50h:25m | 1441 |
| 51h:10m | 1444 |
| 51h:55m | 1445 |
| 52h:45m | 1444 |
| 53h:30m | 1440 |
| 54h:15m | 1438 |
| 55h:00m | 1444 |
| 55h:50m | 1444 |
| 56h:35m | 1443 |
| 57h:20m | 1446 |
| 58h:05m | 1449 |
| 58h:55m | 1449 |
| 59h:40m | 1446 |
| 60h:25m | 1443 |
| 61h:10m | 1445 |
| 62h:00m | 1436 |
| 62h:45m | 1450 |
| 63h:30m | 1442 |
| 64h:15m | 1440 |
| 65h:05m | 1443 |
| 65h:50m | 1436 |
| 66h:35m | 1433 |
| 67h:20m | 1442 |
| 68h:10m | 1444 |
| 68h:55m | 1444 |
| 69h:40m | 1446 |
| 70h:25m | 1442 |
| 71h:15m | 1441 |
| 72h:00m | 1440 |

Staph Aureus Vancomycin 1000MIC 4

| Time | SA 1000MIC VANCO EXP 4 |
| --- | --- |
| 0h:05m | 1586 |
| 0h:50m | 1600 |
| 1h:40m | 1636 |
| 2h:25m | 1657 |
| 3h:10m | 1674 |
| 3h:55m | 1683 |
| 4h:45m | 1693 |
| 5h:30m | 1704 |
| 6h:15m | 1711 |
| 7h:00m | 1724 |
| 7h:50m | 1723 |
| 8h:35m | 1731 |
| 9h:20m | 1737 |
| 10h:05m | 1744 |
| 10h:55m | 1749 |
| 11h:40m | 1754 |
| 12h:25m | 1758 |
| 13h:10m | 1755 |
| 14h:00m | 1765 |
| 14h:45m | 1765 |
| 15h:30m | 1769 |
| 16h:15m | 1776 |
| 17h:05m | 1778 |
| 17h:50m | 1778 |
| 18h:35m | 1782 |
| 19h:20m | 1783 |
| 20h:10m | 1780 |
| 20h:55m | 1781 |
| 21h:40m | 1786 |
| 22h:25m | 1786 |
| 23h:15m | 1787 |
| 24h:00m | 1789 |
| 24h:05m | 1439 |
| 24h:50m | 1435 |
| 25h:40m | 1435 |
| 26h:25m | 1434 |
| 27h:10m | 1437 |
| 27h:55m | 1436 |
| 28h:45m | 1442 |
| 29h:30m | 1441 |
| 30h:15m | 1439 |
| 31h:00m | 1444 |
| 31h:50m | 1437 |
| 32h:35m | 1438 |
| 33h:20m | 1443 |
| 34h:05m | 1436 |
| 34h:55m | 1445 |
| 35h:40m | 1442 |
| 36h:25m | 1441 |
| 37h:10m | 1434 |
| 38h:00m | 1439 |
| 38h:45m | 1435 |
| 39h:30m | 1437 |
| 40h:15m | 1439 |
| 41h:05m | 1432 |
| 41h:50m | 1439 |
| 42h:35m | 1438 |
| 43h:20m | 1436 |
| 44h:10m | 1434 |
| 44h:55m | 1436 |
| 45h:40m | 1440 |
| 46h:25m | 1434 |
| 47h:15m | 1433 |
| 48h:00m | 1438 |
| 48h:05m | 1424 |
| 48h:50m | 1423 |
| 49h:40m | 1420 |
| 50h:25m | 1420 |
| 51h:10m | 1422 |
| 51h:55m | 1416 |
| 52h:45m | 1417 |
| 53h:30m | 1419 |
| 54h:15m | 1423 |
| 55h:00m | 1418 |
| 55h:50m | 1419 |
| 56h:35m | 1420 |
| 57h:20m | 1420 |
| 58h:05m | 1422 |
| 58h:55m | 1418 |
| 59h:40m | 1421 |
| 60h:25m | 1421 |
| 61h:10m | 1423 |
| 62h:00m | 1417 |
| 62h:45m | 1419 |
| 63h:30m | 1411 |
| 64h:15m | 1413 |
| 65h:05m | 1412 |
| 65h:50m | 1411 |
| 66h:35m | 1408 |
| 67h:20m | 1410 |
| 68h:10m | 1409 |
| 68h:55m | 1406 |
| 69h:40m | 1406 |
| 70h:25m | 1405 |
| 71h:15m | 1402 |
| 72h:00m | 1404 |

Staph Aureus Vancomycin 1000MIC 5

| Time | SA 1000MIC VANCO EXP 8 |
| --- | --- |
| 0h:05m | 1228 |
| 0h:50m | 1113 |
| 1h:40m | 1140 |
| 2h:25m | 1150 |
| 3h:10m | 1160 |
| 3h:55m | 1171 |
| 4h:45m | 1174 |
| 5h:30m | 1176 |
| 6h:15m | 1177 |
| 7h:00m | 1182 |
| 7h:50m | 1188 |
| 8h:35m | 1195 |
| 9h:20m | 1199 |
| 10h:05m | 1204 |
| 10h:55m | 1209 |
| 11h:40m | 1214 |
| 12h:25m | 1213 |
| 13h:10m | 1221 |
| 14h:00m | 1225 |
| 14h:45m | 1227 |
| 15h:30m | 1235 |
| 16h:15m | 1233 |
| 17h:05m | 1233 |
| 17h:50m | 1238 |
| 18h:35m | 1233 |
| 19h:20m | 1241 |
| 20h:10m | 1244 |
| 20h:55m | 1245 |
| 21h:40m | 1242 |
| 22h:25m | 1247 |
| 23h:15m | 1247 |
| 24h:00m | 1248 |
| 24h:05m | 1228 |
| 24h:50m | 1231 |
| 25h:40m | 1235 |
| 26h:25m | 1235 |
| 27h:10m | 1243 |
| 27h:55m | 1246 |
| 28h:45m | 1243 |
| 29h:30m | 1245 |
| 30h:15m | 1250 |
| 31h:00m | 1249 |
| 31h:50m | 1251 |
| 32h:35m | 1250 |
| 33h:20m | 1252 |
| 34h:05m | 1250 |
| 34h:55m | 1250 |
| 35h:40m | 1250 |
| 36h:25m | 1250 |
| 37h:10m | 1251 |
| 38h:00m | 1253 |
| 38h:45m | 1250 |
| 39h:30m | 1248 |
| 40h:15m | 1253 |
| 41h:05m | 1249 |
| 41h:50m | 1250 |
| 42h:35m | 1249 |
| 43h:20m | 1250 |
| 44h:10m | 1248 |
| 44h:55m | 1250 |
| 45h:40m | 1250 |
| 46h:25m | 1247 |
| 47h:15m | 1247 |
| 48h:00m | 1256 |
| 48h:05m |  |
| 48h:50m |  |
| 49h:40m |  |
| 50h:25m |  |
| 51h:10m |  |
| 51h:55m |  |
| 52h:45m |  |
| 53h:30m |  |
| 54h:15m |  |
| 55h:00m |  |
| 55h:50m |  |
| 56h:35m |  |
| 57h:20m |  |
| 58h:05m |  |
| 58h:55m |  |
| 59h:40m |  |
| 60h:25m |  |
| 61h:10m |  |
| 62h:00m |  |
| 62h:45m |  |
| 63h:30m |  |
| 64h:15m |  |
| 65h:05m |  |
| 65h:50m |  |
| 66h:35m |  |
| 67h:20m |  |
| 68h:10m |  |
| 68h:55m |  |
| 69h:40m |  |
| 70h:25m |  |
| 71h:15m |  |
| 72h:00m |  |

Staph Aureus Vancomycin 1000MIC 6

| Time | SA 1000MIC VANCO EXP 8 |
| --- | --- |
| 0h:05m | 1125 |
| 0h:50m | 1134 |
| 1h:40m | 1157 |
| 2h:25m | 1171 |
| 3h:10m | 1179 |
| 3h:55m | 1186 |
| 4h:45m | 1194 |
| 5h:30m | 1201 |
| 6h:15m | 1200 |
| 7h:00m | 1203 |
| 7h:50m | 1212 |
| 8h:35m | 1218 |
| 9h:20m | 1220 |
| 10h:05m | 1228 |
| 10h:55m | 1231 |
| 11h:40m | 1238 |
| 12h:25m | 1244 |
| 13h:10m | 1247 |
| 14h:00m | 1247 |
| 14h:45m | 1252 |
| 15h:30m | 1257 |
| 16h:15m | 1260 |
| 17h:05m | 1264 |
| 17h:50m | 1265 |
| 18h:35m | 1272 |
| 19h:20m | 1273 |
| 20h:10m | 1276 |
| 20h:55m | 1278 |
| 21h:40m | 1278 |
| 22h:25m | 1280 |
| 23h:15m | 1280 |
| 24h:00m | 1285 |
| 24h:05m | 1264 |
| 24h:50m | 1264 |
| 25h:40m | 1265 |
| 26h:25m | 1265 |
| 27h:10m | 1267 |
| 27h:55m | 1266 |
| 28h:45m | 1269 |
| 29h:30m | 1268 |
| 30h:15m | 1268 |
| 31h:00m | 1267 |
| 31h:50m | 1269 |
| 32h:35m | 1269 |
| 33h:20m | 1266 |
| 34h:05m | 1268 |
| 34h:55m | 1268 |
| 35h:40m | 1269 |
| 36h:25m | 1268 |
| 37h:10m | 1270 |
| 38h:00m | 1267 |
| 38h:45m | 1269 |
| 39h:30m | 1269 |
| 40h:15m | 1268 |
| 41h:05m | 1269 |
| 41h:50m | 1270 |
| 42h:35m | 1268 |
| 43h:20m | 1267 |
| 44h:10m | 1267 |
| 44h:55m | 1266 |
| 45h:40m | 1267 |
| 46h:25m | 1265 |
| 47h:15m | 1264 |
| 48h:00m | 1269 |
| 48h:05m |  |
| 48h:50m |  |
| 49h:40m |  |
| 50h:25m |  |
| 51h:10m |  |
| 51h:55m |  |
| 52h:45m |  |
| 53h:30m |  |
| 54h:15m |  |
| 55h:00m |  |
| 55h:50m |  |
| 56h:35m |  |
| 57h:20m |  |
| 58h:05m |  |
| 58h:55m |  |
| 59h:40m |  |
| 60h:25m |  |
| 61h:10m |  |
| 62h:00m |  |
| 62h:45m |  |
| 63h:30m |  |
| 64h:15m |  |
| 65h:05m |  |
| 65h:50m |  |
| 66h:35m |  |
| 67h:20m |  |
| 68h:10m |  |
| 68h:55m |  |
| 69h:40m |  |
| 70h:25m |  |
| 71h:15m |  |
| 72h:00m |  |

Staph Aureus Vancomycin 1000MIC 7

| Time | SA 1000MIC VANCO EXP W |
| --- | --- |
| 0h:05m | 876 |
| 0h:50m | 854 |
| 1h:40m | 873 |
| 2h:25m | 883 |
| 3h:10m | 892 |
| 3h:55m | 897 |
| 4h:45m | 902 |
| 5h:30m | 906 |
| 6h:15m | 908 |
| 7h:00m | 909 |
| 7h:50m | 910 |
| 8h:35m | 919 |
| 9h:20m | 918 |
| 10h:05m | 920 |
| 10h:55m | 921 |
| 11h:40m | 921 |
| 12h:25m | 926 |
| 13h:10m | 925 |
| 14h:00m | 925 |
| 14h:45m | 929 |
| 15h:30m | 927 |
| 16h:15m | 930 |
| 17h:05m | 929 |
| 17h:50m | 931 |
| 18h:35m | 930 |
| 19h:20m | 929 |
| 20h:10m | 932 |
| 20h:55m | 932 |
| 21h:40m | 929 |
| 22h:25m | 931 |
| 23h:15m | 933 |
| 24h:00m | 927 |
| 24h:05m | 991 |
| 24h:50m | 986 |
| 25h:40m | 986 |
| 26h:25m | 984 |
| 27h:10m | 985 |
| 27h:55m | 984 |
| 28h:45m | 986 |
| 29h:30m | 978 |
| 30h:15m | 983 |
| 31h:00m | 980 |
| 31h:50m | 981 |
| 32h:35m | 982 |
| 33h:20m | 979 |
| 34h:05m | 977 |
| 34h:55m | 980 |
| 35h:40m | 978 |
| 36h:25m | 975 |
| 37h:10m | 976 |
| 38h:00m | 974 |
| 38h:45m | 977 |
| 39h:30m | 974 |
| 40h:15m | 976 |
| 41h:05m | 968 |
| 41h:50m | 963 |
| 42h:35m | 965 |
| 43h:20m | 966 |
| 44h:10m | 967 |
| 44h:55m | 965 |
| 45h:40m | 964 |
| 46h:25m | 966 |
| 47h:15m | 968 |
| 48h:00m | 966 |
| 48h:05m | 1038 |
| 48h:50m | 1042 |
| 49h:40m | 1039 |
| 50h:25m | 1042 |
| 51h:10m | 1040 |
| 51h:55m | 1040 |
| 52h:45m | 1042 |
| 53h:30m | 1039 |
| 54h:15m | 1040 |
| 55h:00m | 1044 |
| 55h:50m | 1042 |
| 56h:35m | 1043 |
| 57h:20m | 1041 |
| 58h:05m | 1042 |
| 58h:55m | 1043 |
| 59h:40m | 1039 |
| 60h:25m | 1041 |
| 61h:10m | 1039 |
| 62h:00m | 1037 |
| 62h:45m | 1042 |
| 63h:30m | 1038 |
| 64h:15m | 1038 |
| 65h:05m | 1023 |
| 65h:50m | 1027 |
| 66h:35m | 1028 |
| 67h:20m | 1027 |
| 68h:10m | 1023 |
| 68h:55m | 1025 |
| 69h:40m | 1025 |
| 70h:25m | 1024 |
| 71h:15m | 1018 |
| 72h:00m | 1020 |

Staph Aureus Vancomycin 1000MIC 8

| Time | SA 1000MIC VANCO EXP W |
| --- | --- |
| 0h:05m | 885 |
| 0h:50m | 853 |
| 1h:40m | 867 |
| 2h:25m | 876 |
| 3h:10m | 885 |
| 3h:55m | 889 |
| 4h:45m | 891 |
| 5h:30m | 895 |
| 6h:15m | 898 |
| 7h:00m | 899 |
| 7h:50m | 903 |
| 8h:35m | 903 |
| 9h:20m | 904 |
| 10h:05m | 904 |
| 10h:55m | 908 |
| 11h:40m | 908 |
| 12h:25m | 910 |
| 13h:10m | 908 |
| 14h:00m | 910 |
| 14h:45m | 912 |
| 15h:30m | 915 |
| 16h:15m | 914 |
| 17h:05m | 919 |
| 17h:50m | 915 |
| 18h:35m | 917 |
| 19h:20m | 916 |
| 20h:10m | 918 |
| 20h:55m | 918 |
| 21h:40m | 916 |
| 22h:25m | 920 |
| 23h:15m | 921 |
| 24h:00m | 921 |
| 24h:05m | 1191 |
| 24h:50m | 1174 |
| 25h:40m | 1171 |
| 26h:25m | 1170 |
| 27h:10m | 1164 |
| 27h:55m | 1166 |
| 28h:45m | 1162 |
| 29h:30m | 1160 |
| 30h:15m | 1161 |
| 31h:00m | 1160 |
| 31h:50m | 1159 |
| 32h:35m | 1158 |
| 33h:20m | 1156 |
| 34h:05m | 1156 |
| 34h:55m | 1150 |
| 35h:40m | 1155 |
| 36h:25m | 1151 |
| 37h:10m | 1153 |
| 38h:00m | 1153 |
| 38h:45m | 1152 |
| 39h:30m | 1154 |
| 40h:15m | 1153 |
| 41h:05m | 1152 |
| 41h:50m | 1150 |
| 42h:35m | 1151 |
| 43h:20m | 1148 |
| 44h:10m | 1146 |
| 44h:55m | 1150 |
| 45h:40m | 1146 |
| 46h:25m | 1144 |
| 47h:15m | 1149 |
| 48h:00m | 1147 |
| 48h:05m | 1072 |
| 48h:50m | 1070 |
| 49h:40m | 1076 |
| 50h:25m | 1070 |
| 51h:10m | 1072 |
| 51h:55m | 1073 |
| 52h:45m | 1074 |
| 53h:30m | 1075 |
| 54h:15m | 1075 |
| 55h:00m | 1073 |
| 55h:50m | 1074 |
| 56h:35m | 1075 |
| 57h:20m | 1074 |
| 58h:05m | 1075 |
| 58h:55m | 1070 |
| 59h:40m | 1071 |
| 60h:25m | 1071 |
| 61h:10m | 1070 |
| 62h:00m | 1071 |
| 62h:45m | 1068 |
| 63h:30m | 1068 |
| 64h:15m | 1068 |
| 65h:05m | 1072 |
| 65h:50m | 1069 |
| 66h:35m | 1066 |
| 67h:20m | 1064 |
| 68h:10m | 1066 |
| 68h:55m | 1065 |
| 69h:40m | 1064 |
| 70h:25m | 1064 |
| 71h:15m | 1061 |
| 72h:00m | 1061 |

Staph Aureus Vancomycin 1000MIC 9

| Time | SA 1000MIC VANCO EXP W |
| --- | --- |
| 00:05 | 1383 |
| 02:25 | 1437 |
| 04:45 | 1449 |
| 07:05 | 1450 |
| 09:20 | 1466 |
| 11:40 | 1469 |
| 14:00 | 1482 |
| 16:20 | 1491 |
| 18:40 | 1491 |
| 21:00 | 1493 |
| 23:15 | 1494 |
| 25:35 | 1489 |
| 27:55 | 1491 |
| 30:15 | 1487 |
| 32:35 | 1488 |
| 34:55 | 1484 |
| 37:10 | 1478 |
| 39:30 | 1479 |
| 41:50 | 1467 |
| 44:10 | 1466 |
| 46:30 | 1466 |
| 48:50 | 1461 |
| 51:05 | 1465 |
| 53:25 | 1456 |
| 55:45 | 1453 |
| 58:05 | 1451 |
| 60:25 | 1447 |
| 62:45 | 1445 |
| 65:00 | 1440 |
| 67:20 | 1438 |
| 69:40 | 1432 |
| 72:00 | 1430 |

Staph Aureus Vancomycin 1000MIC 10

| Time | SA 1000MIC VANCO EXP W |
| --- | --- |
| 00:05 | 1074 |
| 02:25 | 1125 |
| 04:45 | 1139 |
| 07:05 | 1147 |
| 09:20 | 1166 |
| 11:40 | 1177 |
| 14:00 | 1188 |
| 16:20 | 1192 |
| 18:40 | 1194 |
| 21:00 | 1199 |
| 23:15 | 1198 |
| 25:35 | 1200 |
| 27:55 | 1200 |
| 30:15 | 1195 |
| 32:35 | 1192 |
| 34:55 | 1194 |
| 37:10 | 1195 |
| 39:30 | 1189 |
| 41:50 | 1187 |
| 44:10 | 1184 |
| 46:30 | 1182 |
| 48:50 | 1177 |
| 51:05 | 1178 |
| 53:25 | 1175 |
| 55:45 | 1171 |
| 58:05 | 1169 |
| 60:25 | 1161 |
| 62:45 | 1166 |
| 65:00 | 1165 |
| 67:20 | 1161 |
| 69:40 | 1160 |
| 72:00 | 1151 |
